# Agroclimatic Metrics for the Main Stone Fruit Producing Areas in Spain in Current and Future Climate Change Scenarios: Implications From an Adaptive Point of View

**DOI:** 10.3389/fpls.2022.842628

**Published:** 2022-06-08

**Authors:** Jose A. Egea, Manuel Caro, Jesús García-Brunton, Jesús Gambín, José Egea, David Ruiz

**Affiliations:** ^1^Fruit Breeding Group, Department of Plant Breeding, CEBAS-CSIC, Murcia, Spain; ^2^Murcia Institute of Agri-Food Research and Development, Murcia, Spain; ^3^ENAE Business School, University of Murcia, Murcia, Spain

**Keywords:** *Prunus*, stone fruit, adaptation, chill accumulation, phenology, frost risk, varietal choice, agroclimatic metrics

## Abstract

Stone fruit production has enormous economic importance in Spain. Cultivation locations for these fruit species (i.e., peach, apricot, plum, and sweet cherry) cover wide and climatically diverse geographical areas within the country. Climate change is already producing an increase in average temperatures with special intensity in certain areas like the Mediterranean ones. These changes lead to a decrease in the accumulated chill, which can have a profound impact on the phenology of *Prunus* species like stone fruits due to, e.g., difficulties to cover the chilling requirements to break endodormancy, the occurrence of late frost events, or abnormal early high temperatures. All these factors can severely affect fruit production and quality and therefore provoke very negative consequences from the socio-economic point of view in the incumbent regions. Thus, characterization of current cultivation areas in terms of agroclimatic variables (e.g., chill and heat accumulation and probabilities of frost and early abnormal heat events), based on data from 270 weather stations for the past 20 years, is carried out in this work to produce an informative picture of the current situation. Besides, future climatic projections from different global climate models (data retrieved from the Meteorological State Agency of Spain—AEMET) up to 2065 for two Representative Concentration Pathway scenarios (i.e., RCP4.5 and RCP8.5) are also analyzed. Using the current situation as a baseline and considering the future scenarios, information on the current and future adaptive suitability of the different species/cultivars to the different growing areas can be inferred. This information could be the basis of a decision support tool to help the different stakeholders to take optimal decisions regarding current and future stone fruit or other temperate species cultivation in Spain.

## Introduction

**FIGURE 1:**
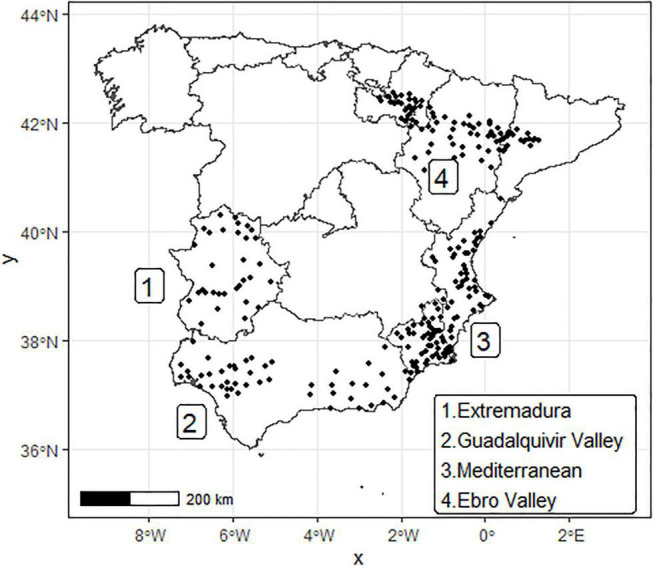
Location of the 270 weather stations (black dots) used for this study.

Spain is one of the main world producers of stone fruits (i.e., peach, apricot, plum, and sweet cherry) with an average annual production of around 2 million tons. Cultivation of these fruits has a very important economic role in the country, covering around 140,260 ha (FAOSTAT, 2019). The main growing areas in Spain for these cultivars are located in areas with different agroclimatic characteristics: from warm areas like Guadalquivir Valley and a large part of the Mediterranean area to cold areas like northern Extremadura, Ebro valley, and some interior locations of the Mediterranean area (see Figure 1). Since these crops require sufficient winter chill to break endodormancy to avoid production problems (Atkinson et al., 2013), assessing relevant agroclimatic metrics for current and future scenarios in the growing areas can help to (i) analyze the current potential production problems (Hatfield et al., 2019), (ii) study the climate change influence over such metrics in each area (Campoy et al., 2011b; Luedeling et al., 2011; Luedeling, 2012), (iii) select the optimal locations to fulfill the cultivars chilling requirements (CRs), avoiding frost episodes that can damage flowers and thus ruin the production in the mid and long term (Julian et al., 2007; Guo et al., 2015, 2019; Chmielewski et al., 2018), and (iv) select the best agricultural practices and technologies to mitigate the effect of climate change (Campoy et al., 2010; Mahmood et al., 2018).

Chill and heat requirements (Fadón et al., 2020b) or level of frost damage (Miranda et al., 2005) of the current cultivated species/cultivars can be coupled with the agroclimatic metrics in the different areas to build decision tools that help producers and other stakeholders to design optimal production and economic policies for medium and long term. Available modeling tools to process large series of climate and phenological already serve as the basis to build the above-mentioned decision tools (Luedeling, 2019; Luedeling et al., 2021; Miranda et al., 2021). Climate projections in the Mediterranean basin reveal that the effects of global warming can be especially severe in this area (Giorgi and Lionello, 2008; MedECC, 2020; IPCC, 2021), thus anticipation measures are critical to avoid future production problems, which could seriously affect the economy of certain regions like the ones presented in this study (Olesen and Bindi, 2002; Benmoussa et al., 2018).

Different research studies have determined the negative influence of global warming on the production of temperate fruits and nuts in different regions across the planet. The main causes are related to the decrease in winter chill although the increase of frost risks due to the expected advance in blooming and flowering is also taken into account in some studies. For instance, Fernandez et al. forecasted a decrease in winter chill needed for deciduous fruit production in Chile, with expected negative impacts in northern areas of the country. At the same time, they projected significant reductions of frost probabilities during the most plausible period of budburst for deciduous fruit trees for all the considered sites (Fernandez et al., 2020); Lorite et al. analyzed phenomena like lack of winter chill, frost risk, and warm conditions during flowering in the Iberian Peninsula for some almond cultivars coupling climate projections and phenological information. They found that, in general (and depending on the considered cultivar), (i) the lack of winter chill will be more pronounced in the Mediterranean coast and the Guadalquivir Valley, (ii) warm conditions during flowering will be more intense in the Central Plateau and Ebro Valley, and (iii) the risk of frost will be reduced to particular areas of the Northern Plateau and Northern Hilly Areas (Lorite et al., 2020). Benmoussa et al. projected important future winter chill reductions in Tunisia that can significantly affect the production of some fruits and nuts. For example, for the most pessimistic scenario, only low-chill almond cultivars could be viable. In other scenarios, some pistachios and peach cultivars could be viable even in the long term for the North-Western part of the country (Benmoussa et al., 2020); Fraga and Santos considered both the future chilling and heat accumulation and their impacts on the production of different fruits in Portugal. They projected strong declines in winter chilling that will more severely affect the inner-most regions of the country. The northern apple growing areas will be particularly exposed to chilling reduction. The authors also projected increases in heat accumulation, with a higher impact in the southern and coastal areas of the country. They highlighted that this fact may increase the risk of frost damage due to the advance of phenological stages (Rodríguez et al., 2019, 2021; Fraga and Santos, 2021) compared the current situation of the production areas of some temperate fruits in Spain with future climate change scenarios regarding chill accumulation. They forecasted important chill losses in some areas (e.g., South-East or Gualdalquivir area) even in the near future. For the far future (>2070), these authors stated that considering current growing areas, plum, almond, and apple cultivars can be seriously affected by the lack of chill (Rodríguez et al., 2019, 2021).

In this study, we assessed the main agroclimatic variables related to stone fruit adaptation in different regions within Spain, including those where the most important stone fruits production takes place using data from 270 weather stations during the period 2000–2020. This is accompanied by future temperature projections to estimate the chill and heat accumulation evolution and the future probabilities of frost and early abnormal heat events compared with the current situation. This information can be very useful for taking the optimal decisions related to setting up new orchards, relocating current ones, or selecting the optimal cultivars to obtain profit in the long run.

The main contribution of this study is that we analyzed at the same time different agroclimatic variables related to stone fruit adaptation. Not only the chill accumulation to fulfill CRs as performed in the study by Rodríguez et al. (2019, 2021) but also heat accumulation for proper flowering, frost risks, and a variable rarely quantified in the literature: the probability of abnormal heat events in winter that can boost endodormancy release with a negative impact on fruit production, quality, and yield, as it has been observed in warm areas within the past years. We used data from a very dense network of weather stations that provide accurate metrics for the current situation. We focused on the current producing areas as decisions regarding warming adaptation will probably be taken in those areas, where the suitable technologies and knowledge are well settled down. In such areas, crop relocations would produce undesirable socio-economic consequences and depopulation. Further, for characterizing the current situation, we used real hourly temperatures instead of estimated ones, which confer more accuracy to the results compared with other studies where hourly temperatures are interpolated from daily ones. The used resolution (∼5 km) is finer than in other similar studies in Spain (Rodríguez et al., 2019, 2021; Lorite et al., 2020) and helps to make decisions even at a local level.

## Materials and Methods

### Climatic Data and Agroclimatic Variables

Climatic data from 340 weather stations located in the main stone fruits producing areas in Spain (see Figure 1) were used to assess the agroclimatic metrics. Data comprised the main climatic variables, including mean, maximum, and minimum temperature (°C), relative humidity (%), rainfall (mm), evapotranspiration (ETo, mm), and solar radiation (W/m^2^). Incomplete records and issues were found in some of the considered stations. After applying the Spanish regulation (UNE 500540, 2004), a final number of 270 stations was selected. Hourly temperature data were complete except for empty hours corresponding to maintenance events that were not filled as they consisted in a negligible percentage of the total. Mean hourly temperatures in the period 2000–2020 were used to calculate the main agroclimatic variables, including chill and heat accumulations as well as probabilities of potentially harmful frost and abnormal heat events in winter. The number of complete years per station varies per station: from 5 to 21 years (median = 20) depending on the station.

Chill accumulation for each season was calculated from the 1st of November until the 28th of February of the following year. Utah (Richardson et al., 1974) and Dynamic (Fishman et al., 1987) models were used to perform this calculation. Heat accumulation for each season was calculated from the 1st of January to the 8th of April (around 14 weeks) using the Richardson (Richardson et al., 1974) and Anderson (Anderson et al., 1986) models, which provide the results in growing degree hours (GDHs). Probabilities of frost and abnormal heat events were calculated per week as follows: for each week, a frost event occurs if the temperature falls below −1°C during at least three consecutive hours. Then, the probability of occurrence of frost events in a particular week is defined as the number of times that week had at least one frost event during the study period divided by the number of years considered. Similarly, an abnormal heat event occurs if the temperature rises above 25°C for at least three consecutive hours. Then, the probability of occurrence of abnormal heat events is calculated as explained for frost events. Week 1 started at the 1st of January. For frost events, weeks from 2 to 10 were considered as representative potential dangerous weeks. First weeks in the range (i.e., week 2 to week 5–6) would be the most dangerous ones in warm areas, whereas the rest (i.e., weeks 5–6 to week 10) would be the critical ones in cold areas. For abnormal heat events, the considered period ranged from week 49 of the previous year (beginning of December) to 8 (end of February) when these events could boost early dormancy release associated to later production problems.

### Future Scenarios

Regarding future scenarios, temperature projections calculated by the Spanish State Meteorological Agency (AEMET) were used. AEMET has been producing in recent years a set of reference downscaled climate change projections over Spain either applying statistical downscaling techniques to the outputs of the global climate models (GCMs) or making use of the information generated by dynamical downscaling techniques through European projects or international initiatives such as PRUDENCE, ENSEMBLES, and EURO-CORDEX (Amblar-Francés et al., 2018). In this study, we used the projected daily temperatures (i.e., maximum and minimum) using statistical downscaling based on artificial neural networks. This has been evaluated as a suitable method to produce climate projections in the current and future scenarios in Spain while reducing the GCMs model biases (Hernanz et al., 2022a,b) over a grid of 5 km resolution. Two temporal horizons have been considered, namely, 2025–2045 (characterized by 2035) and 2045–2065 (characterized by 2055) to provide results for short and medium term. Two representative concentration pathways, i.e., RCP4.5 and RCP8.5, were considered (van Vuuren et al., 2011). Of note, eleven GCMs were used in this study (Table 1). Results were presented using an *ensemble* methodology (Semenov and Stratonovitch, 2010; Wallach et al., 2018) where the average values of the projected metrics (e.g., chill and heat accumulation or probabilities) computed by all the models were used in subsequent steps. Hourly temperatures to calculate the agroclimatic indexes were simulated from daily ones using the chillR package (Luedeling, 2019).

**TABLE 1:** List of global climate models used in this study.

Model	Institution	References
bcc-csm1-1-m	Beijing Climate Center—Climate System Model 1.1	Wu et al., 2014
BNU-ESM	Beijing Normal University	Ji et al., 2014
CanESM2	Canadian Earth System Model	Chylek et al., 2011
CMCC-CM	Centro Euro-Mediterraneo sui Cambiamenti Climatici	Scoccimarro et al., 2011
GFDL-ESM2G	Geophysical Fluid Dynamics Laboratory—Earth System Models	Delworth et al., 2006
inmcm4	Institute of Numerical Mathematics	Volodin et al., 2010
IPSL-CM5A-LR	Institut Pierre Simon Laplace—Climate Model 5A	Dufresne et al., 2013
MIROC-ESM	Model for Interdisciplinary Research on Climate	Watanabe et al., 2011
MPI-ESM-LR	Max-Planck-Institute für Meteologie	Giorgetta et al., 2013
MPI-ESM-MR		
MRI-CGCM3	Meteorological Research Institute (Japan)	Yukimoto et al., 2012

To compare the agroclimatic variables in the present and future scenarios, the actual locations of the weather stations were compared with their closest points from the grid. Maximum, minimum, and mean distances from the weather stations to their closest points in the grid were 3.87, 0.26, and 2.14 km, respectively. In all cases (current and future scenarios), an interpolated area around the considered weather stations (i.e., no further than 50 km away from the closest weather station) was calculated using the inverse distance weighting method.

## Results

### Chill Accumulation

**FIGURE 2:**
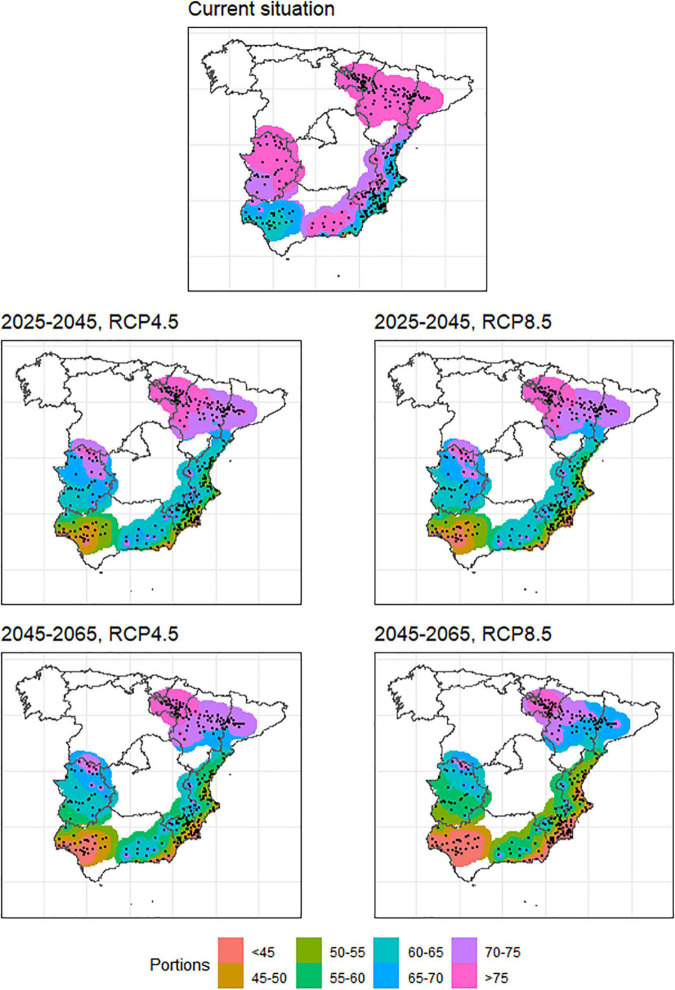
Chill accumulation in the main stone production areas in Spain for the current situation (approximately 2000–2020), two time horizons (2025–2045 and 2045–2065) and two future scenarios (RCP4.5 and RCP8.5).

As pointed out above, two models were used to calculate the chill accumulation, namely, the Utah (in chill units) and the Dynamic model (in portions). Using the mean values of the total accumulated chill within the whole period for all stations, a very high correlation was found between both indexes (*R*^2^ = 0.95, Supplementary Figure 1). Therefore, results are presented using only one of them (portions). Figure 2 shows the spatial patterns of mean chill portions over the different considered periods. In the current situation, we can see that there are several geographical areas with high chill accumulation (≥75 portions), like the Ebro Valley, northern Extremadura, and some interior areas in the Mediterranean. Only in the Mediterranean and Guadalquivir Valley, warm areas with chill accumulation below 60 portions (even below 50 in some isolated areas) are found. The future scenarios show a clear decrease of accumulated chill in warm areas, in northern Extremadura and some interior areas of the Mediterranean. The decrease of accumulated chill in the Ebro Valley will be produced in the eastern part of that area, while the interior will accumulate significant winter chill even in the most pessimistic scenario (e.g., 2055_RCP8.5). The effects of global warming over winter chill decline are more intense in the 2055_RCP8.5 scenario as expected. Supplementary Tables 1–4 show the mean chill accumulation in the considered period (1st November to end of February) in portions for all locations and models in every considered future scenario. The mean value of the outputs of the eleven models is shown, as well as the registered accumulated chill for the period 2000–2020 for comparison purposes.

**FIGURE 3:**
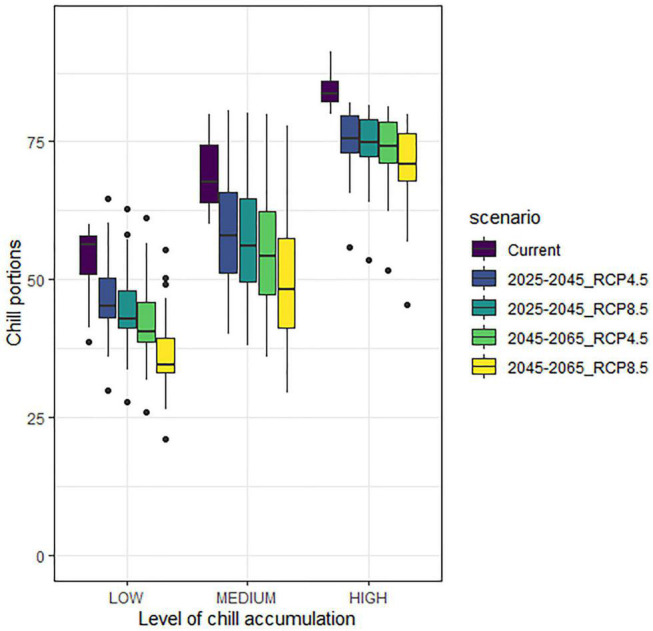
Boxplots of accumulated chill in all scenarios for low (<60 portions), medium (between 60 and 80 portions), and high (>80 portions) chill accumulation stations, referred to the current scenario.

To check if the expected chill accumulation decline will have a similar influence over the locations depending on their current chill accumulation, a classification of the 270 weather stations was performed, dividing them in terms of mean accumulated portions in the current scenario: low accumulation (<60 portions, 34 stations), medium accumulation (between 60 and 80 portions, 121 stations), and high accumulation (above 80 portions, 115 stations). Figure 3 shows the boxplots of the accumulated portions in every scenario for the three types of locations. The observed chill accumulation decline is as expected according to each scenario. In terms of differences in median values between current and future scenarios, it seems that the three types of locations present the same behavior (which means that the percentual losses are higher in low accumulation areas). However, the spread of the data is very different. Low and high chill accumulation areas show lower dispersion (with some outliers in the low end of the distribution) than medium areas, which present a higher dispersion but no outliers. The analysis of these outliers for high chill accumulation areas reveals that the outlier for all the four future scenarios corresponds to an interior Mediterranean location (Játiva). For low chill accumulation areas, the outlier in every case (including the current scenario) corresponds to a coastal Mediterranean location (Almería). The outliers for the high end of the distribution in low chill accumulation areas correspond to interior locations in the Mediterranean (i.e., Montesa, Callosa de Sarriá, and Murcia) although they could be artifacts since projections forecast more chill accumulation in future than in the current scenario. They could be caused by the possible climatic differences between the actual location of the weather stations and their closest point in the grid for future projections.

### Heat Accumulation

**FIGURE 4:**
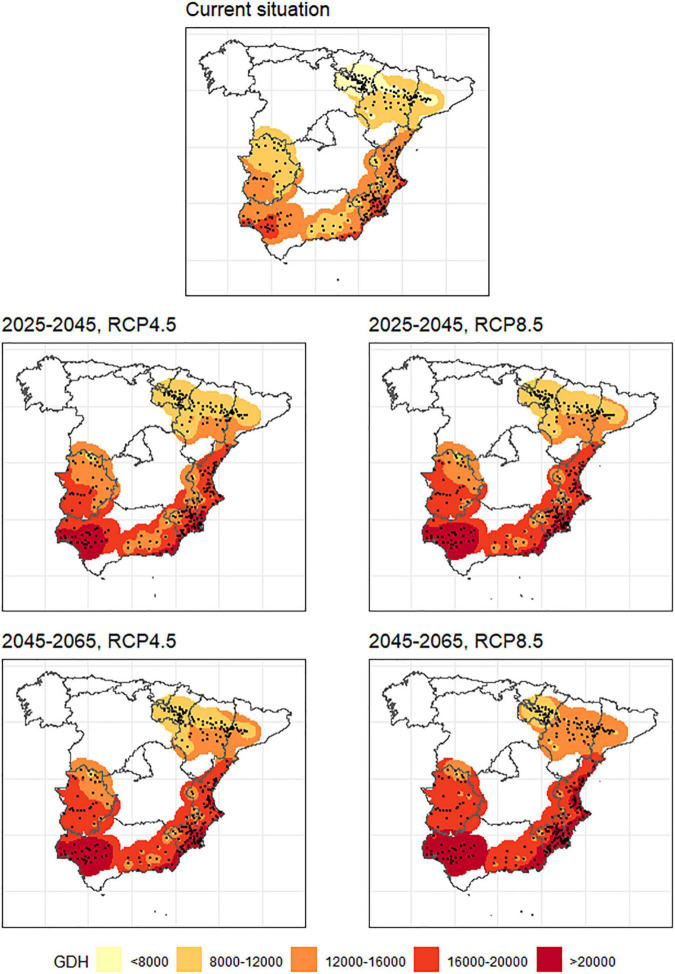
Heat accumulation in the main stone production areas in Spain for the current situation (approximately 2000–2020), two time horizons (2025–2045 and 2045–2065) and two future scenarios (RCP4.5 and RCP8.5).

Heat accumulation was calculated using two models (i.e., Richardson and Anderson models) similarly to chill accumulation. A high correlation was also found between the outcomes of both models (*R*^2^ = 0.998, Supplementary Figure 2). Therefore, results are presented using only the outcomes of the Anderson model. Figure 4 shows the spatial patterns of mean GDH over the different considered periods. All the scenarios regarding GDH seem to inversely correlate with their corresponding chill accumulation scenarios (Figure 2). Places where chill accumulation is low present high heat accumulation and vice-versa. As chill accumulation decreases in future scenarios, heat accumulation increases proportionally in each area. For instance, the Pearson correlation coefficient between the lost chill accumulation and the gained heat accumulation for current and 2055_RCP8.5 scenarios is 0.68 (*p*-value < 1e^–15^).

Like in the chill accumulation case, the effects of GDH increase are more intense in the 2055_RCP8.5 scenario as expected. Supplementary Tables 5–8 show the mean heat accumulation in the considered period (1st January–8th April) in GDH for all locations and models in every considered scenario. The mean value of the outputs of the eleven models is shown, as well as the registered accumulated heat for the period 2000–2020 for comparison purposes.

### Frost and Abnormal Heat Events Probabilities

**FIGURE 5:**
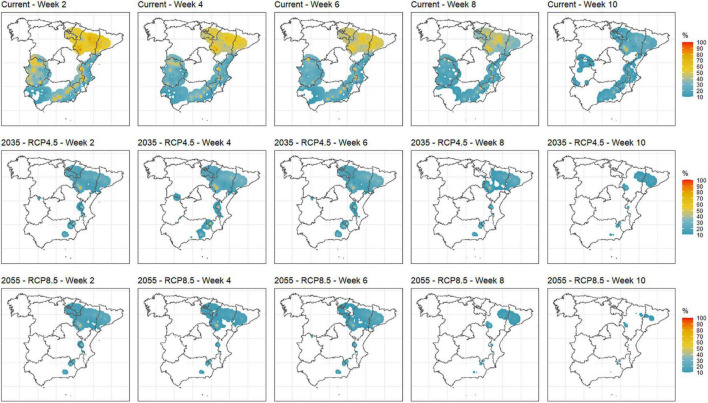
Probability of frost events in the main stone production areas in Spain for weeks 2 to 10 for the current, 2035_RCP4.5 and 2055_RCP8.5 scenarios.

The probability of frost events as defined above is shown in Figure 5 comparing weeks 2–10 for the current and 2035_RCP4.5 and 2055_RCP8.5 scenarios (only probabilities ≥ 10%). In the current situation, significant probabilities of frost events were recorded especially in areas of the Ebro Valley but also northern Extremadura and interior areas of the Mediterranean. Frost probabilities decrease from weeks 2 to 10 as expected, but some particular locations in the Ebro Valley still present a significant probability of frost on week 10. The analyzed future scenarios in Figure 5 are the most optimistic (i.e., 2035_RCP4.5) and pessimistic (i.e., 2055_RCP8.5), respectively, in terms of temperature rise. The probability of frost events vanishes from Extremadura and decreases in all areas, whereas just reduced areas of the Ebro Valley and some isolated areas in the interior Mediterranean show probabilities above 10% even in week 10. Like in the current situation, frost probabilities decrease from weeks 2 to 10. Remarkably, 2035_RCP4.5 and 2055_RCP8.5 scenarios present similar pictures in terms of probabilities of frost events, revealing that the Ebro Valley and some interior Mediterranean locations will undergo frost events in all the considered scenarios.

**FIGURE 6:**
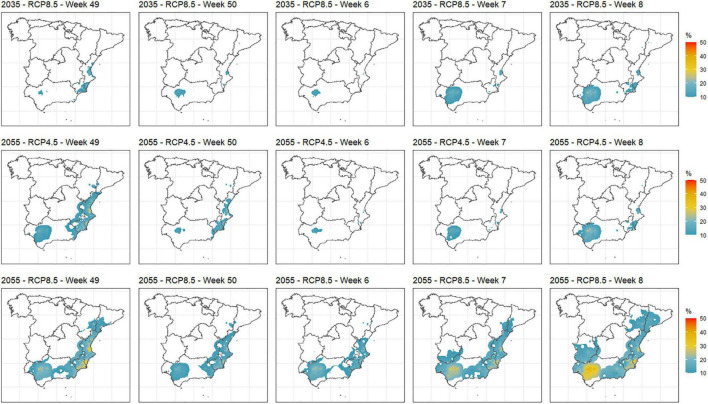
Probability of abnormal heat events in the main stone production areas in Spain for weeks 49 (beginning of December) to 8 (end of February) in the 2035_RCP8.5, 2055_RCP4.5, and 2055_RCP8.5 scenarios.

Regarding abnormal heat events as defined above, Figure 6 shows the probability of occurrence of such events from weeks 49 (i.e., beginning of December of the previous year) to 8 (i.e., end of February). Only probabilities ≥ 10% are considered. Therefore, maps for current and 2035_RCP4.5 scenarios are not shown since just a few isolated locations comply with that value. The shown future scenarios show that Guadalquivir Valley and locations near the coastal Mediterranean area will undergo the highest number of abnormal heat events in winter. Clear differences appear between 2035_RCP8.5 and 2055_RCP8.5 scenarios. The areas that will undergo these types of events are expanded in the latter covering interior Mediterranean locations and some areas of the Ebro Valley.

## Discussion and Conclusion

This study tried to characterize the main stone fruits producing areas of Spain using historic agroclimatic data (particularly temperatures) from 270 weather stations spread throughout such areas and compare the results with future projections in two time horizons and RCP scenarios. The study areas were selected based on the fact that current and future decisions to be made regarding the cultivation of stone fruits (i.e., peach, apricot, plum, and sweet cherry) will be mainly taken within the current producing areas, where the knowledge and technology for growing these crops are strongly installed. Thus, this study does not focus on other future potential locations for stone fruit cultivation.

The main computed variables, i.e., chill and heat accumulation, reveal that the considered areas are quite diverse from the agroclimatic point of view and that climate change will have an important impact, especially in the warmest areas even in the medium term. The models used to calculate either of them (i.e., Utah and Dynamic for chill and Richardson and Anderson for heat accumulation) show very high correlations as previously found by Ruiz et al. (2007, 2018).

Important chill accumulation reductions are projected in all areas, which agrees with previous studies in Mediterranean areas (Benmoussa et al., 2018, 2020; Rodríguez et al., 2019; Delgado et al., 2021; Fraga and Santos, 2021). The chill accumulation decrease will be similar in absolute values in all the studied regions, but the warmest ones (i.e., Mediterranean area and Guadalquivir Valley) can be much more affected in terms of stone fruits cultivation suitability since their current situation is already a limitation for many cultivars. In cold areas like Ebro Valley and Extremadura, the chill accumulation decline will not be in principle an obstacle to continue cultivating, although in some particular cold locations in Extremadura and the Mediterranean, the chill accumulation decline will be more intense than in other cold locations. It is to note that, according to Figure 3, a sudden drop in chill accumulation between the current situation and the near future is observed. The resolution of the used grid, even if fine (∼5 km) can be a cause of this effect. Other possible sources of discrepancies leading to exaggerated differences between the projected and the real values could be the remaining GCM model biases not being completely minimized during the downscaling process, or the fact that we are comparing calculations carried out with real hourly temperatures (i.e., current scenario) and calculations carried out with idealized temperature curves derived from projected daily maximum and minimum temperatures (Linvill, 1990) for the future scenarios. Similar sudden drops in the near future were also observed by Rodríguez et al., who forecasted a decrease of up to 30 chilling portions for the period 2021–2050 in some locations in Spain (Rodríguez et al., 2019), which agrees with our results. Benmoussa et al. (2020), Delgado et al. (2021), and Fraga and Santos (2021) also reported sudden drops between the historic and future scenarios in Tunisia, Portugal, and Asturias (North Spain), respectively. Like in our case, these studies also showed that no important differences for accumulated chill appear in the near future regardless of the RCP considered. Contrarily to chill accumulation, heat accumulation will rise in all the scenarios (especially in 2055_RCP8.5 as expected), and its evolution is inverse to this of chill accumulation. This was also observed by Fraga and Santos (2021) for Portugal.

Probabilities of frost and abnormal heat events in the weeks where they can importantly affect yield and production (e.g., late frost or abnormal heat events before endodormancy release) were computed as well. For the current scenario, frost events are more frequent in cold areas, as expected. Abnormal heat events in key weeks have been concentrated in the Mediterranean area during the past years but with very low probabilities. Future estimations for these variables show that frost events in weeks where stone fruit production can be affected (Miranda et al., 2005; Julian et al., 2007) will decrease as the century advances and will be less frequent for RCP8.5, which agrees with previous studies (Leolini et al., 2018). However, some areas of the Ebro Valley and particular interior locations of the Mediterranean areas will still undergo a significant number of frost events within the incumbent weeks even in the warmest scenario (i.e., 2055_RCP8.5, Figure 5). The definition of a frost event in terms of temperature and exposure time is closely related to the phenological stage of the incumbent cultivar (Miranda et al., 2005). Given the large variety of possible stone fruit cultivars, from very low to very high CR, and the number of analyzed locations, from cold to warm, establishing particular cultivar/location frost event definitions is not feasible in this study due to the huge volume of information involved. These types of studies are usually carried out using a few locations and/or cultivars, like the one performed by Lorite et al. (2020) for almonds in Spain, Fernandez et al. (2020) in Chile, who computed minimum temperatures below 0°C during the blooming period of the most representative deciduous fruit tree species cultivated at each of the nine considered sites, or Parker et al. (2021) who considered different temperatures and phenological stages for three species (i.e., almonds, avocados, and oranges) but also performed a general characterization of the area by considering three temperatures (0, −2, and +2°C) and exposure time. Our choice of −1°C and at least three consecutive hours aims at characterizing the evolution of the frost events rather than relating the specific damage to particular cultivars, which would suppose a different study. This definition was adopted after retrieving experts’ opinions. Due to the wide number of cultivars in terms of CR and HR and the diversity of temperature regimes in the considered areas in this study, we selected those weeks (from 2 to 10) where all (or most) combinations of cultivar/location could be susceptible of undergoing frost damages according to their phenological stage. For decision-making purposes, producers should select the map that best fits their particular situation (i.e., cultivar/location) to make the optimal decision. In general, warm areas and/or early flowering cultivars will be related to earlier weeks in the considered range, whereas cold areas and/or late flowering cultivars will be related to later weeks in the considered range. Abnormal heat events in winter that can boost an early endodormancy release, which negatively affects production (Viti and Monteleone, 1995; Rodrigo and Herrero, 2002; Ladwig et al., 2019), will be increased mainly in Guadalquivir Valley, coastal Mediterranean areas, and also in Extremadura and some areas of the Ebro Valley in mid- or late February (Figure 6). Quantification of this metric is usually not addressed in the literature but can provoke important production issues in warm areas as has been observed in recent years. Again, setting 25°C or above for at least three consecutive hours to define such an event was motivated by experts’ opinions. Similarly as with probabilities of frost events, we selected those weeks (from 49 to 8) where all (or most) combinations of cultivar/location could be susceptible of being affected by these events according to their phenological stage. In general, warm areas and/or early flowering cultivars will be related to earlier weeks in the considered range, whereas cold areas and/or late flowering cultivars will be related to later weeks in the considered range.

The agroclimatic metrics calculated in this study provide valuable information for producers to select the most suitable cultivars in every producing area from an adaptive point of view. Each cultivar has its CRs to break endodormancy (Campoy et al., 2011b; Fadón et al., 2020b). A decline in chill accumulation as projected in future scenarios may cause that currently grown cultivars do not fulfill their CR in certain areas, especially those of the Mediterranean and the Guadalquivir Valley areas, which are already warm. This would involve an incomplete endodormancy release that affects the fruit trees in three main aspects, namely, flower bud drops (and thus poor flowering), delay in flowering and sprouting, and lack of uniformity in both processes, which lead to serious productive problems (Legave et al., 1983; Erez, 2000; Atkinson et al., 2013). All of these can produce important economic losses to producers. In this context, knowledge about CR for different cultivars is crucial although the currently available information is relatively scarce in stone fruit trees (Fadón et al., 2020b), including peach (Maulión et al., 2014), apricot (Ruiz et al., 2007), plum (Ruiz et al., 2018), and sweet cherry (Alburquerque et al., 2008).

In warm areas like the Mediterranean and Guadalquivir Valley, where the accumulated chill is below 60 portions in the current situation, early ripening cultivars with CR between 30 and 60 portions are grown. CR fulfillment for these cultivars can be at risk in all the analyzed future scenarios (Figure 2). To ensure the adaptive suitability of the different species/cultivars to these areas, a relocation may be needed, and some of the cultivars should be moved to close areas (interior zones in the Mediterranean area or toward Extremadura in the case of the Guadalquivir Valley) where the CR will be fulfilled even in the future scenarios, and the frost risks are expected to decrease. In this context, the introduction or development of cultivars with very low CR becomes a crucial target to be considered in breeding programs of the incumbent species/cultivars, especially to be suitable for the warm areas where current cultivars’ adaptation will be at risk in future scenarios. Otherwise, these areas will not be able to keep their productive and economic activities related to stone fruit production. Apart from this, different agronomic practices and strategies could also be applied to minimize the chill accumulation decline in these areas at least locally. The application of bio-stimulants to break endodormancy before fulfilling the CR or the use of shading nets during different dormancy stages have already been described in warm areas for stone fruit production (Gilreath and Buchanan, 1981; Erez, 1987; Costa et al., 2004; Campoy et al., 2010; Petri et al., 2014), although further research and optimization must be carried out to make these techniques more effective and promote their systematic use. In contrast, in the coldest producing areas like the Ebro Valley, northern Extremadura, and some interior locations in the Mediterranean area, fewer frost events are expected, which could allow earlier cultivars than current ones, which would expand the number of viable cultivars and, therefore, the offer to the market with positive economic consequences for the area. Overall, in all the producing areas, it is crucial to consider the currently grown cultivars and analyze which are at the edge of their CR fulfillment to substitute or move them or to introduce the management practices described above to ensure the adaptation to the new climate change scenarios.

Regarding heat accumulation, the future scenarios forecast an increase of this variable in all the considered areas (Figure 4). In warm and intermediate areas, this variable is not as decisive as the chill accumulation but can have a relevant impact on phenology, producing an advance in flowering dates and thus increasing the potential frost injury risk (Mosedale et al., 2015; Unterberger et al., 2018; Ma et al., 2019). As an additional point, this flowering advance will involve a ripening advance as well (Peñuelas and Filella, 2001; Campoy et al., 2011b), which must be taken into account by producers to strategically put their products on the markets. In contrast, in cold areas, the lack of heat accumulation in the current situation can harm the phenological development and fruit growth (Fadón et al., 2020a). These currently cold areas will be favored by the forecasted heat accumulation increase for future scenarios. As shown in Figure 6, abnormal heat events will be more frequent in future scenarios on dates where the fruit trees have not yet released endodormancy, especially in warm areas like the Guadalquivir Valley and Mediterranean locations. These events can have a very negative effect when the CR are partially covered (around 60–70%), inducing an incomplete dormancy release that may involve vegetative and flowering problems, with a negative impact on fruit set and yield (Rodrigo and Herrero, 2002; Campoy et al., 2011a).

In any case, changes in the chill and heat accumulation regimes do not have a common effect on all cultivars and their locations since some compensation effects can take place regarding the balance chill/heat accumulation in terms of endodormancy release or flowering dates prediction (Pope et al., 2014). Besides, agroclimatic characterization of locations at a very local scale may require a particular calibration of data due to the spatial heterogeneity (Lorite et al., 2020) to make the best decisions regarding the optimal cultivar selections. The results presented in this study can be useful not only for stone fruit production but also for other temperate fruits with enormous importance in the incumbent areas, e.g., grapevines in La Rioja (Ebro Valley) or others. These results can be the basis of decision support systems to aid producers in making optimal strategic decisions (e.g., cultivar selection, relocation, and implementation of mitigation management practices) in the medium and long term.

## Data Availability Statement

The original contributions presented in the study are included in the article/Supplementary Material, further inquiries can be directed to the corresponding authors.

## Author Contributions

MC, JG-B, JG, and DR conceived and designed the study. MC provided the agroclimatic data for the current scenario. JAE performed the calculations for future scenarios. JAE and DR wrote the main part of the manuscript. JE provided information about technical agronomic aspects. JG managed the innovation project that funded this research. All authors revised the document and approved the submitted version.

## Conflict of Interest

The authors declare that the research was conducted in the absence of any commercial or financial relationships that could be construed as a potential conflict of interest.

## Publisher’s Note

All claims expressed in this article are solely those of the authors and do not necessarily represent those of their affiliated organizations, or those of the publisher, the editors and the reviewers. Any product that may be evaluated in this article, or claim that may be made by its manufacturer, is not guaranteed or endorsed by the publisher.

## Supplementary Material

Supplementary Figure 1 Correlation between mean accumulated portions and chill units for the current scenario in all the weather stations.

Supplementary Figure 2 Correlation between mean accumulated GDH for Anderson and Richardson models for the current scenario in all the weather stations.
