# Supplementary material for: Agroclimatic Metrics for the Main Stone Fruit Producing Areas in Spain in Current and Future Climate Change Scenarios: Implications From an Adaptive Point of View
Source: Front Plant Sci. 2022 Jun 8;13:842628. doi: 10.3389/fpls.2022.842628 (PMC9213681; doi:10.3389/fpls.2022.842628)
Supplement: Supplementary file 1 [file Data_Sheet_1.PDF]

Supplementary Table 1. Mean accumulated portions (1st November - end February) for 2025-2045, RCP 4.5 at each location.
The last column shows the chilling accumulation for the current situation, for comparison purposes
M1: bcc-csm1-1-m; **M2:** BNU-ESM; **M3:** CanESM2; **M4:** CMCC-CM; **M5:** GFDL-ESM2G; **M6:** inmcm4
M7: IPSL-CM5A-LR; **M8:** MIROC-ESM; **M9:** MPI-ESM-LR; **M10:** MPI-ESM-MR; **M11:** MRI-CGCM3

Municipality	Longitude	Latitude	M1	M2	M3	M4	M5	M6	M7	M8	M9	M10	M11	MEAN	CURRENT
Campo de Mirra	-0,7729762	38,679366	77,4	77,7	76,8	74,6	76,4	79,4	75,3	75,4	75,0	72,2	75,1	75,9	81,4
Villajoyosa	-0,2561866	38,527917	48,1	44,5	50,0	31,7	47,6	54,5	44,3	43,8	45,1	43,2	49,8	45,7	52,9
Ondara	0,0065631	38,818581	50,3	47,5	53,2	35,3	49,6	58,8	48,0	45,0	48,7	45,0	53,2	48,6	56,1
Denia Gata	0,082579	38,792724	53,7	50,6	56,5	38,5	52,4	59,8	49,7	49,6	50,9	48,2	54,3	51,3	61,2
Pinoso	-1,060721	38,427413	76,5	76,0	76,2	72,6	75,3	78,8	73,3	74,0	73,7	70,2	74,6	74,7	81,6
Monforte del Cid	-0,7303963	38,398862	57,5	53,5	59,0	43,5	53,1	61,4	52,5	51,8	53,4	48,5	57,9	53,8	64,4
Crevillente	-0,7831581	38,240831	48,1	44,1	49,4	32,2	45,0	52,6	43,5	43,7	44,1	39,8	49,7	44,8	53,2
Almoradi	-0,7745396	38,031431	48,0	43,8	51,6	32,0	45,7	53,0	45,2	44,3	44,9	40,9	49,9	45,4	55,6
Callosa de Sarria	-0,1044988	38,650249	63,0	60,1	64,7	50,8	60,7	68,3	58,3	59,1	60,0	56,5	62,1	60,3	49,7
Pilar de la Horadada	-0,8125284	37,868588	43,5	39,3	47,6	27,9	42,1	49,7	40,4	40,0	40,6	36,9	46,9	41,3	55,7
Catral	-0,8055704	38,153124	47,3	43,0	49,9	30,3	43,5	51,1	42,9	42,8	43,3	39,5	48,8	43,9	56,3
Altea	-0,0795078	38,603643	54,5	51,4	58,0	40,7	53,2	61,2	50,9	51,3	52,3	49,0	55,2	52,5	50,2
Planes	-0,3529076	38,78476	72,4	71,9	73,2	64,7	70,6	76,8	68,8	69,5	70,6	66,8	70,2	70,5	72,1
Villena	-0,8753684	38,595491	69,8	68,0	68,7	62,2	67,1	72,5	65,0	65,6	66,3	62,2	68,5	66,9	79,1
Agost	-0,6498214	38,421512	61,4	57,3	61,9	48,6	56,5	64,6	55,4	55,6	57,6	52,3	60,5	57,4	65,2
Almansa	-1,1075837	38,903228	79,0	79,3	78,7	78,3	78,6	80,7	78,3	77,8	77,5	76,1	76,2	78,2	80,2
Ontur	-1,4957688	38,622866	73,1	72,0	71,9	66,9	70,4	74,6	69,1	68,8	69,8	66,0	71,9	70,4	82,1
Caudete	-0,9798818	38,734665	75,8	74,5	74,9	70,3	73,2	77,9	72,2	72,1	72,9	68,9	73,8	73,3	75,4
La Mojonera	-2,7043824	36,787318	49,3	48,3	50,5	37,0	45,6	51,8	43,5	49,1	45,9	39,7	50,4	46,5	44,0
Almeria	-2,4024534	36,835404	32,0	30,2	34,9	16,2	29,7	34,9	28,5	31,8	30,4	25,2	35,8	29,9	38,6
Nijar	-2,1580794	36,95057	44,1	42,0	45,6	29,3	40,4	47,4	39,4	43,5	40,8	35,1	45,1	41,1	62,7
Tabernas	-2,3023755	37,091315	62,5	62,6	62,6	53,5	58,4	64,0	56,1	61,3	57,3	52,3	61,6	59,3	71,1
Fiñana	-2,8388277	37,156718	75,2	76,0	76,0	75,5	74,7	77,2	73,7	75,0	73,4	70,6	73,6	74,6	86,1
Cuevas de Almanzora	-1,7704017	37,389125	50,0	49,7	50,1	37,9	46,1	53,2	44,5	50,0	46,0	40,8	50,3	47,2	64,7
Huercal-overa	-1,8842832	37,412428	57,7	57,1	57,2	46,1	52,1	59,1	50,6	56,0	52,3	47,3	56,6	53,8	64,4
Cuevas de Almanzora	-1,800522	37,256757	39,1	36,3	39,8	22,8	35,1	41,6	34,1	39,0	35,5	30,3	41,4	35,9	50,3
Adra	-2,9923491	36,746758	38,8	32,7	38,3	30,5	35,4	42,0	35,4	36,8	34,3	30,7	41,9	36,1	41,2
Tijola	-2,457021	37,371918	69,8	70,8	69,6	64,5	66,5	71,0	63,7	68,8	65,7	60,1	68,6	67,2	81,0

Totana	-1,5130934	37,732459	56,1	51,9	57,2	41,8	52,6	60,4	50,9	50,5	52,2	46,6	56,4	52,4	70,8
Alhama	-1,4167602	37,7922	56,0	51,8	57,6	41,6	52,1	59,6	50,6	50,6	52,6	46,9	56,4	52,3	65,8
Librilla	-1,3382889	37,899373	60,2	56,5	60,8	48,0	57,1	63,6	54,9	55,3	56,3	51,5	60,4	56,8	64,9
Mazarron	-1,4009916	37,56215	48,5	46,7	51,8	31,7	45,7	52,4	45,5	46,9	45,6	42,4	48,7	46,0	49,4
Mazarron	-1,3788416	37,614572	51,3	48,4	53,3	36,0	48,2	54,2	47,4	48,8	48,4	43,9	50,5	48,2	58,6
Zalamea de la Serena	-5,6910276	38,678704	71,9	63,6	71,2	64,7	65,7	72,5	66,5	66,8	70,1	64,0	72,6	68,1	78,2
Monterrubio de la Serena	-5,3836134	38,591582	73,8	67,4	73,0	67,9	68,7	74,2	67,9	70,3	72,0	66,3	74,1	70,5	83,3
Don Benito	-5,9062469	38,930491	65,8	57,6	65,5	59,5	61,1	67,3	62,7	61,7	66,6	59,6	70,4	63,4	75,4
Villagonzalo	-6,1858738	38,837247	69,3	56,0	67,0	64,1	61,8	69,4	65,4	62,5	67,1	58,8	73,6	65,0	73,8
Jerez de los Caballeros	-6,7369024	38,281336	68,2	56,8	66,7	57,2	59,9	68,9	62,6	61,5	64,3	56,5	69,3	62,9	75,3
Olivenza	-7,0578251	38,720921	68,7	56,4	67,3	57,2	59,6	69,9	63,0	60,8	63,7	55,9	70,6	63,0	73,5
Villafranca de los Barros	-6,3485695	38,575591	71,3	59,5	69,2	61,7	62,9	71,4	65,8	64,3	67,3	59,9	71,9	65,9	74,7
Merida	-6,3192869	38,845149	69,4	57,4	67,6	63,5	62,8	69,6	65,9	62,1	67,8	59,2	73,5	65,3	75,7
Azuaga	-5,7077922	38,391445	75,5	68,7	74,5	69,6	69,4	75,2	69,7	71,2	74,2	67,8	75,4	71,9	81,2
Puebla de alcocer	-5,0955855	39,074643	73,4	68,3	72,8	69,0	70,3	75,3	70,2	71,1	73,2	67,7	75,5	71,5	78,9
Don Benito	-5,858992	38,984723	65,6	56,4	65,9	59,8	60,0	67,8	61,9	61,2	67,3	59,6	70,5	63,3	74,0
Badajoz	-6,827838	38,877039	67,9	55,2	66,7	62,8	61,6	68,6	64,5	60,7	66,1	58,4	72,7	64,1	73,4
Pueblonuevo del Guadiana	-6,7328012	38,912998	67,5	55,2	66,1	62,3	61,4	68,3	63,3	60,8	65,5	57,8	72,4	63,7	73,3
Lobon	-6,6655535	38,860185	68,2	55,7	65,5	62,3	61,4	67,4	64,5	61,3	65,5	57,4	72,7	63,8	72,7
Arroyo de San Servan	-6,4728164	38,858246	70,0	58,1	67,8	64,3	62,8	69,9	66,0	63,0	68,2	59,8	73,3	65,8	74,4
Villar de Reina	-5,742601	39,102295	70,0	62,5	69,4	64,8	65,4	71,5	66,3	65,9	70,7	64,1	72,5	67,6	78,9
Cartagena	-0,9508754	37,688833	47,4	45,6	50,1	31,0	44,1	50,2	44,0	45,9	43,7	41,1	47,4	44,6	58,8
Murcia	-1,1227711	37,831265	55,6	52,9	58,1	41,6	53,9	60,4	51,3	52,6	52,5	47,6	56,9	53,1	62,8
Fuente alamo	-1,1292626	37,748269	46,0	42,4	49,4	30,8	43,5	51,0	42,4	41,9	43,0	39,2	48,5	43,5	63,7
Cartagena	-1,070786	37,676671	46,0	42,1	48,7	29,6	43,7	51,3	42,0	41,8	42,0	38,1	48,6	43,1	60,0
Cartagena	-0,8037931	37,611152	48,1	45,7	50,1	31,9	45,5	51,2	44,4	46,0	45,1	42,8	47,9	45,3	58,7
Fuente alamo	-1,2380371	37,699008	54,5	49,9	56,1	38,7	51,5	58,4	49,5	49,6	50,2	45,7	55,3	50,8	63,2
Casatejada	-5,6781	39,867824	71,6	63,6	70,9	66,8	67,2	72,7	67,5	68,1	72,0	66,1	74,3	69,2	81,3
Aldehuela del Jerte	-6,2302346	40,008316	71,3	62,1	70,8	68,7	66,7	70,6	67,4	67,6	70,6	63,0	76,8	68,7	79,5
Moraleja	-6,6759606	40,046357	70,3	61,8	70,9	66,5	66,7	71,5	67,9	63,4	69,3	62,4	73,9	67,7	80,0
Coria	-6,5458096	39,978094	71,0	61,1	71,1	67,7	67,1	71,9	68,4	66,6	69,9	63,0	76,4	68,6	78,4
Madrigalejo	-5,5954391	39,135847	70,6	62,3	70,0	65,1	66,8	72,6	66,8	66,3	70,6	63,9	73,3	68,0	80,7
Valdesalor	-6,4785825	39,377191	72,3	62,6	71,2	66,6	66,7	73,8	69,2	66,1	70,3	63,3	74,3	68,7	80,3

Peraleda de la Mata	-5,4639595	39,861132	74,2	66,5	73,5	69,0	69,4	75,9	70,7	70,8	73,6	68,1	75,6	71,6	82,4
Tejeda de tietar	-5,8600359	39,960042	74,4	72,0	73,9	71,0	73,7	75,6	71,7	76,8	75,0	69,5	78,5	73,8	77,6
Casar de Palomero	-6,3056933	40,298554	80,3	76,1	79,8	78,2	77,6	81,5	78,1	76,7	79,0	73,5	80,6	78,3	84,5
Madroñera	-5,7623097	39,464885	79,7	75,7	79,1	76,5	76,5	81,0	76,9	77,2	79,1	74,3	79,4	77,8	84,2
Guadalupe	-5,3482094	39,387141	78,6	73,8	78,4	75,6	75,8	80,2	76,0	76,4	78,1	73,2	78,8	76,8	85,3
Alcantara	-6,8981123	39,746563	70,1	58,3	68,2	64,9	63,9	70,5	66,5	63,0	68,4	60,6	73,8	66,2	78,5
Jarandilla de la Vega	-5,6463392	40,101413	81,1	79,4	81,0	79,0	80,3	81,4	79,4	81,7	80,1	76,5	81,3	80,1	84,0
Gargantilla	-5,9414268	40,239041	81,7	82,1	82,2	80,3	80,9	82,3	80,8	81,7	80,4	77,3	80,6	80,9	84,8
Talayuela	-5,5642306	40,011198	74,5	72,4	74,8	70,3	73,5	75,5	72,3	75,3	74,4	68,8	77,6	73,6	81,1
Valdastillas	-5,8687982	40,141215	83,1	83,0	83,4	82,3	82,3	83,2	82,5	81,6	81,5	78,7	80,3	82,0	83,5
Cieza	-1,3097249	38,235442	69,4	67,8	69,1	61,1	66,9	72,9	64,3	65,9	65,8	61,3	69,0	66,7	64,4
Ulea	-1,2578423	38,191392	62,3	59,9	62,9	50,1	58,5	65,4	56,6	57,9	58,7	53,4	62,7	58,9	71,6
Cieza	-1,4963438	38,283888	63,8	60,7	63,1	52,3	59,0	66,2	57,7	58,7	59,8	54,3	63,1	59,9	61,1
Calasparra	-1,6947638	38,253487	64,8	61,7	63,6	52,2	59,8	66,9	58,5	58,5	60,5	55,7	64,0	60,5	72,8
Calasparra	-1,5850018	38,269499	61,6	58,2	61,0	50,3	57,9	64,3	56,5	55,9	57,9	52,5	61,5	58,0	73,3
Caravaca	-1,980057	38,043911	75,6	74,9	75,4	70,0	74,6	77,7	72,9	73,7	73,2	69,8	72,2	73,6	88,1
Cehegin	-1,6828994	38,110901	71,0	69,0	70,1	62,6	67,7	73,2	66,6	66,9	67,4	62,3	70,2	67,9	73,0
Moratalla	-1,813186	38,196653	73,1	70,5	73,2	62,2	71,2	75,1	68,7	70,8	70,3	66,7	69,3	70,1	79,0
Cehegin	-1,7798922	38,104477	74,3	72,4	74,1	64,2	72,5	76,4	69,8	72,6	71,2	68,2	70,6	71,5	76,3
Moratalla	-2,0961142	38,1145	79,3	80,3	78,2	79,1	79,2	79,4	78,8	79,0	77,5	76,8	76,5	78,5	87,1
Vall de Uxo	-0,2304536	39,795861	57,9	55,6	59,9	45,8	56,4	66,0	55,1	53,3	56,9	53,7	59,7	56,4	64,2
Onda	-0,2444114	39,954016	63,7	62,1	62,1	53,8	60,0	68,8	57,8	56,5	61,6	57,0	63,0	60,6	69,2
San Rafael del Rio	0,3675272	40,594077	64,8	63,0	63,4	56,0	60,8	70,6	58,8	56,6	62,1	57,3	64,9	61,7	75,5
Benicarlo	0,4014538	40,411511	57,5	55,4	56,7	47,7	55,7	65,2	52,0	49,9	55,6	50,9	58,8	55,0	67,6
Castellon	-0,1191495	39,989342	62,3	60,4	60,1	52,1	59,0	68,3	55,8	55,8	59,2	54,5	62,1	59,1	70,3
Burriana	-0,1057138	39,887849	53,6	51,8	56,4	40,0	53,0	61,7	50,7	49,0	51,9	49,2	56,6	52,2	66,9
Ribera de Cabanes	0,1464314	40,133934	63,6	61,4	62,1	53,4	60,9	70,6	57,1	56,6	60,7	56,5	63,1	60,6	67,4
Nules	-0,1683946	39,877237	55,7	54,0	58,8	43,3	56,4	64,1	53,6	52,2	56,0	52,3	58,4	55,0	65,4
Segorbe	-0,4830876	39,817295	70,8	70,1	71,2	60,9	69,2	75,1	67,2	66,7	68,7	64,8	69,1	68,5	74,4
Baza	-2,7677154	37,564477	69,8	71,7	69,9	64,0	65,9	70,8	63,5	68,0	65,2	59,3	68,5	67,0	83,9
Puebla de Don Fadrique	-2,3817176	37,876115	77,8	78,7	76,6	76,2	77,7	78,9	77,2	77,4	76,2	73,7	74,7	76,8	82,3
Loja	-4,138128	37,1693	69,1	64,2	70,4	67,5	65,0	71,1	65,2	66,4	67,3	62,2	70,0	67,1	79,5
Iznalloz	-3,5514591	37,416406	71,8	69,8	70,9	69,8	67,0	72,8	65,5	69,4	67,3	62,8	69,7	68,8	86,0

Jerez del Marquesado	-3,1498644	37,190536	74,4	75,2	74,6	73,6	72,9	75,1	72,6	73,6	72,6	69,4	72,1	73,3	84,3
Cadiar	-3,183988	36,923123	71,3	70,0	71,2	70,1	68,6	73,5	67,2	70,3	67,3	63,5	69,9	69,4	81,4
Zafarraya	-4,1538389	36,990314	79,3	78,9	80,3	80,6	78,2	81,0	78,1	79,6	78,4	75,3	78,7	79,0	89,9
Padul	-3,600317	37,018743	71,0	69,7	70,6	70,4	67,4	72,5	66,0	69,7	66,7	62,5	70,4	68,8	80,0
Granada	-3,6385645	37,172054	69,7	67,6	69,2	66,1	65,1	71,6	63,0	67,4	64,6	60,3	67,9	66,6	77,9
Almuñecar	-3,6790578	36,751942	55,0	50,0	54,1	49,4	49,8	58,6	49,6	52,8	50,8	45,9	56,3	52,0	54,1
Gibraleon	-7,0278022	37,318328	44,0	36,4	47,3	32,2	39,6	50,6	43,8	41,3	41,6	34,8	50,3	42,0	59,5
Lepe	-7,2430825	37,302685	45,7	38,5	49,7	33,6	42,7	54,1	44,3	43,2	43,0	36,8	51,0	43,9	57,0
Gibraleon	-7,059841	37,412354	50,5	43,3	52,7	39,4	46,0	57,2	47,7	47,4	47,0	40,7	55,1	47,9	66,0
Moguer	-6,7925285	37,14648	46,7	39,1	49,9	35,1	42,4	53,6	45,1	43,1	44,6	38,1	51,9	44,5	62,3
Niebla	-6,7353478	37,347125	48,0	39,9	51,5	36,5	43,6	54,1	46,5	44,4	45,8	39,3	53,9	45,8	62,5
Aroche	-6,9449915	37,958077	69,7	59,6	68,1	59,1	62,6	70,5	64,4	62,8	65,7	58,6	70,3	64,7	71,4
La puebla de Guzman	-7,2483655	37,552176	57,7	50,9	60,9	47,9	53,9	64,4	55,6	55,0	55,0	48,9	61,5	55,6	69,5
El Campillo	-6,5992719	37,660989	58,4	52,5	61,1	49,3	54,5	63,8	57,1	55,8	56,9	49,8	62,9	56,6	74,5
La Palma del Condado	-6,5415566	37,366968	54,2	45,9	56,9	43,3	49,9	60,4	52,3	51,4	52,0	45,0	58,7	51,8	66,3
Almonte	-6,4765444	37,148345	45,2	36,9	48,6	33,2	41,2	52,7	44,0	42,1	42,8	36,3	51,2	43,1	60,1
Valfarta	-0,1478858	41,531503	75,0	74,1	74,4	76,9	71,4	79,2	70,7	71,1	72,7	69,5	73,4	73,5	83,5
Zaidin	0,2890014	41,637169	74,1	71,9	72,8	76,2	69,8	78,1	69,2	69,4	71,3	67,8	72,3	72,1	79,4
Alcolea de Cinca	0,0731411	41,74095	74,5	73,3	72,7	76,8	70,4	77,8	69,8	70,0	72,1	69,4	73,0	72,7	82,2
Tanarite de Litera	0,3771357	41,780947	74,8	73,9	74,4	75,9	71,0	78,4	70,3	71,0	72,7	69,6	71,9	73,1	81,9
Lanaja	-0,337846	41,786429	77,3	76,2	75,2	78,8	73,1	80,2	73,5	72,7	75,2	72,4	74,9	75,4	83,5
Monzon	0,1273494	41,957687	76,8	76,2	76,8	77,4	73,8	79,7	73,3	74,1	74,2	71,9	73,9	75,3	83,1
Barbastro	0,1126102	42,013471	77,3	77,1	76,0	77,6	74,1	79,5	74,1	73,7	74,3	72,5	74,7	75,5	84,0
Sariñena	-0,1766614	41,771411	76,5	74,7	74,6	77,2	71,5	78,9	71,4	71,5	72,9	69,9	73,3	73,9	83,1
Huesca	-0,3777068	42,105429	77,9	77,9	77,3	79,3	74,6	80,2	75,4	75,5	75,9	73,5	75,2	76,6	83,6
Candasnos	0,094436	41,45994	72,5	71,0	72,4	75,3	68,9	77,2	67,7	68,5	69,9	66,5	70,9	71,0	83,7
Grañen	-0,3560041	41,942469	78,1	77,9	76,9	77,9	74,0	80,9	74,2	74,5	75,6	73,1	75,0	76,2	82,9
Huerto	-0,1365362	41,966019	78,8	78,3	76,8	78,5	75,1	80,5	75,5	74,4	75,9	73,6	76,2	76,7	83,6
Gurrea de Gallego	-0,7311994	41,992829	79,0	78,1	77,7	78,6	75,7	79,4	76,8	77,4	77,8	75,2	75,9	77,4	85,9
Alfantega	0,1477817	41,821958	76,7	76,6	75,9	76,9	73,3	79,2	73,2	73,4	74,2	71,9	74,5	75,1	82,1
Fraga	0,3539314	41,495165	71,8	70,2	70,7	74,9	67,9	77,1	66,2	67,0	68,7	65,9	69,8	70,0	76,9
Tardienta	-0,5075831	41,969367	77,0	76,5	75,5	78,7	73,4	80,8	73,6	73,0	74,6	72,0	75,0	75,5	83,5
San Esteban de Litera	0,3042037	41,882938	77,1	77,1	76,4	78,0	74,2	80,0	74,2	74,1	75,1	72,8	75,1	75,8	82,0

Belver de Cinca	0,2318291	41,742536	75,8	74,0	73,4	77,1	72,0	79,5	70,5	71,7	73,1	70,2	73,5	73,7	83,8
Alberuela de Tubo	-0,2573084	41,883957	76,0	74,8	74,6	77,3	71,7	78,9	71,4	72,1	73,0	70,8	73,5	74,0	84,5
Jumilla	-1,4232837	38,394834	71,5	70,3	70,5	64,2	69,1	73,8	67,0	68,4	67,9	63,3	70,2	68,7	77,7
Yecla	-1,1859032	38,658948	75,0	74,7	74,2	70,4	73,4	77,4	72,1	72,1	72,2	68,9	73,4	73,1	85,6
Yecla	-1,1125211	38,562731	72,5	71,6	71,8	65,8	70,4	74,8	68,3	69,2	68,8	65,4	71,0	70,0	83,7
Jumilla	-1,2407841	38,392588	73,5	72,0	72,3	66,2	70,8	75,5	68,4	69,5	69,5	65,4	71,2	70,4	73,6
Jumilla	-1,3242866	38,31972	66,5	63,9	65,5	55,7	63,2	69,2	60,6	61,2	62,5	57,8	65,5	62,9	74,7
Aitona	0,4609093	41,486913	72,2	69,1	69,7	74,0	67,9	77,1	65,6	67,0	68,9	64,9	70,2	69,7	77,7
Albesa	0,6705502	41,760356	73,6	74,0	72,9	76,9	67,5	76,6	71,2	70,0	71,7	68,7	73,8	72,4	82,6
Alcarras	0,5506119	41,56508	74,2	73,6	73,0	74,9	70,5	78,7	68,4	70,1	71,3	68,5	71,3	72,2	81,5
Alfarras	0,5780224	41,819488	74,4	74,6	74,0	78,1	68,9	77,0	73,2	71,2	72,9	69,9	73,8	73,4	83,0
Algerri	0,6483717	41,801036	74,9	75,4	74,5	78,7	70,1	77,7	73,6	72,4	73,7	71,0	74,3	74,2	80,0
Alguaire	0,5361346	41,742812	76,7	75,8	76,2	77,5	73,1	79,2	73,2	73,7	74,5	71,7	74,1	75,1	84,8
Castellnou de Seana	0,9520619	41,65659	73,8	73,7	73,1	77,3	68,4	77,3	71,5	70,3	72,5	69,1	73,7	72,8	82,6
Cervera	1,2967772	41,662217	78,6	79,1	78,6	81,7	74,3	80,4	77,5	77,0	76,8	74,5	76,5	77,7	83,6
Gimenells i el Pla de la Font	0,3933398	41,658132	75,5	73,8	75,0	76,4	72,3	78,6	70,9	71,7	72,5	70,3	73,1	73,6	83,2
Golmes	0,9248038	41,63641	72,4	73,3	72,7	76,0	67,0	77,3	70,7	68,8	71,9	69,1	73,4	72,1	75,0
Raimat	0,4490319	41,683272	76,8	76,0	74,9	77,9	72,8	79,8	72,4	73,4	74,0	71,3	74,0	74,9	83,1
Balaguer- Monasterio Avellanas	0,7613663	41,879114	76,2	77,1	76,4	79,5	71,7	79,1	75,4	73,4	74,5	72,1	75,3	75,5	83,3
El Canos	1,2041447	41,689385	78,2	78,4	77,7	81,1	73,2	80,4	76,4	75,5	76,4	74,1	76,5	77,1	84,6
El Poal	0,8777387	41,672786	72,4	73,3	72,7	76,1	67,1	77,3	70,7	68,8	71,9	69,2	73,4	72,1	81,3
Sant Marti de Riucorb	1,0885432	41,572353	75,9	76,1	76,0	79,6	70,4	79,0	74,3	73,4	74,4	71,9	75,1	75,1	85,1
Seros	0,4279758	41,463784	72,8	69,8	71,4	73,9	67,6	76,7	65,5	66,6	69,0	64,7	69,2	69,7	78,2
Tarrega	1,1626814	41,666945	75,8	75,8	75,5	80,0	70,5	78,8	73,8	73,1	74,4	71,7	74,8	74,9	80,5
Tornabous	1,0451011	41,68834	75,1	74,6	74,2	77,8	69,3	77,9	72,2	71,8	73,2	69,9	74,4	73,7	80,2
Vallfogona de Balaguer	0,8293888	41,784868	73,5	73,8	73,7	78,0	68,7	77,3	71,9	71,4	73,0	69,4	73,9	73,1	82,5
Vilanova de Segria	0,628392	41,714499	71,6	72,5	72,2	76,7	66,5	75,0	69,6	68,9	70,4	66,9	72,6	71,2	81,9
Lorca	-1,6294551	37,601733	62,8	61,3	63,1	48,8	58,5	64,5	57,4	59,8	59,3	54,5	59,8	59,1	72,8
Lorca	-1,6938893	37,50379	58,2	56,8	57,7	46,7	52,1	59,5	51,0	56,0	52,1	47,3	56,5	54,0	73,8
Agoncillo	-2,2904337	42,468182	82,9	82,5	80,9	81,7	81,0	81,5	81,4	81,6	81,8	79,5	80,9	81,4	89,7
Albelda de Iregua	-2,4718558	42,380733	82,5	81,6	80,8	82,0	81,1	80,9	81,5	81,3	81,7	80,0	80,4	81,2	90,3
Asenjo	-2,1533164	42,340952	83,5	83,0	81,5	82,9	81,1	81,9	81,2	82,2	82,4	80,1	81,3	81,9	91,1
Logroño	-2,5136369	42,43969	82,8	82,8	80,9	82,4	81,1	81,9	81,5	81,5	82,0	80,0	81,1	81,6	91,5

Santa Engracia del Juvera	-2,2629377	42,368971	82,8	83,0	81,3	81,7	81,3	82,0	81,6	82,1	81,9	79,9	81,0	81,7	86,3
Aldea Nueva de Ebro	-1,9048679	42,222598	81,2	81,8	80,0	79,1	79,0	80,6	78,9	79,7	80,4	77,7	80,2	79,9	86,4
Alfaro	-1,7776916	42,152119	81,6	81,7	79,9	77,3	80,0	80,2	79,4	80,6	80,2	78,2	79,1	79,8	88,6
Calahorra	-2,001826	42,334834	81,2	82,1	80,8	79,4	79,0	81,3	79,4	80,7	80,1	77,6	80,7	80,2	90,9
Corvera (Cabreton)	-1,8924542	42,006904	82,2	81,3	80,4	79,7	80,5	81,0	80,4	80,4	81,0	78,4	80,1	80,5	86,1
Igea	-1,9937535	42,05775	81,5	81,1	81,1	81,0	80,7	80,8	81,3	80,8	81,3	79,6	79,6	80,8	88,5
Quel	-2,037178	42,252488	82,3	82,0	80,7	81,0	79,9	81,3	80,3	80,4	80,9	78,6	80,6	80,7	85,4
Rincon de Soto	-1,8508464	42,251583	81,2	81,7	80,1	79,1	79,0	80,7	79,0	79,7	80,3	77,7	80,1	79,9	85,8
Aguilas	-1,5921627	37,418665	45,7	43,2	48,5	29,3	43,0	49,8	42,8	42,9	42,4	39,9	46,9	43,1	48,9
Lorca	-1,8177885	37,855634	75,8	74,4	75,9	67,5	74,8	77,8	72,9	74,4	73,7	70,0	71,6	73,5	83,5
Lorca	-1,623984	37,4878	59,3	58,2	61,7	43,4	56,0	63,4	54,8	57,8	56,0	52,4	57,5	56,4	59,9
Puerto Lumbreras	-1,7255508	37,590472	57,5	55,8	56,7	47,1	51,8	58,5	50,6	54,9	52,4	47,4	56,3	53,6	68,9
Mula	-1,4294482	38,065871	63,5	61,0	63,3	50,5	60,0	67,0	58,1	59,2	59,2	53,9	63,0	59,9	61,4
Mula	-1,46674	38,041031	65,4	62,4	65,3	53,9	62,3	69,4	59,9	60,4	60,9	56,3	65,5	62,0	60,6
Torres de Cotillas	-1,3025362	38,006971	53,7	49,0	55,3	38,5	50,1	58,1	48,1	48,4	49,4	44,7	54,7	50,0	64,2
Molina del Segura	-1,2206884	38,127483	55,6	50,5	57,3	41,5	51,4	59,2	49,5	49,6	52,2	47,0	56,7	51,9	62,5
Molina del Segura	-1,2336707	38,071139	53,8	49,5	55,5	37,2	49,8	57,3	48,0	48,2	49,5	44,9	54,7	49,9	64,6
Abanilla	-1,0655079	38,170041	53,9	49,6	55,6	37,9	49,8	57,6	48,1	48,3	49,5	45,0	54,7	50,0	64,5
Fortuna	-1,1526819	38,161028	54,0	49,5	55,3	39,8	50,3	58,6	48,3	48,8	50,0	43,8	55,9	50,4	63,7
Ojos	-1,3394287	38,113316	60,4	57,4	61,2	48,4	56,3	63,9	55,2	54,9	56,7	51,2	60,5	56,9	52,7
Beniel	-0,9997837	38,034507	51,9	47,6	53,6	36,0	48,5	56,3	46,7	47,7	47,2	43,3	53,8	48,4	63,2
Murcia	-1,2682702	37,898166	62,0	60,1	63,1	50,9	59,6	66,3	57,3	58,2	58,1	54,0	62,4	59,3	48,9
Murcia	-0,9840042	37,977528	54,4	50,7	56,2	38,8	51,4	59,1	49,5	49,3	50,5	45,3	55,4	50,9	56,4
Murcia	-1,1347189	37,940075	58,1	54,2	59,5	45,2	54,6	62,7	53,4	53,4	53,7	49,2	59,3	54,9	61,4
Fitero	-1,8426437	42,046077	81,9	82,1	80,7	79,6	80,7	81,3	80,8	80,7	81,3	79,3	80,2	80,8	85,4
Cascante	-1,7239555	42,034371	81,4	81,5	79,8	77,2	79,6	80,6	79,3	79,6	80,1	77,9	79,0	79,6	86,2
Ablitas	-1,6447131	41,996446	78,5	77,1	77,5	77,0	74,4	79,4	74,3	75,4	76,6	73,5	75,8	76,3	85,9
Murillo el fruto	-1,4871859	42,38498	81,2	81,1	80,0	80,2	79,3	81,8	78,6	80,8	80,3	77,5	79,6	80,1	84,5
Miranda de Arga	-1,8087315	42,511252	82,4	81,9	80,2	80,8	79,9	81,1	81,1	80,9	81,6	79,0	79,7	80,8	86,1
Falces	-1,7925482	42,409669	82,8	82,8	81,6	81,1	80,6	82,4	81,2	82,1	81,7	79,0	81,1	81,5	85,8
Bargota	-2,2992201	42,477657	82,9	82,5	80,9	81,7	81,0	81,5	81,4	81,6	81,8	79,5	80,9	81,4	85,7
Bardenas Reales	-1,5187546	42,295154	81,5	82,3	81,1	81,2	80,3	82,0	79,0	82,0	80,8	78,0	80,2	80,8	86,0
Los Arcos	-2,1845206	42,539308	83,3	82,7	80,5	82,2	81,3	81,8	82,0	82,0	81,9	79,9	80,7	81,7	86,9

Sesma	-2,126631	42,473409	83,2	82,4	81,2	81,8	81,1	81,5	81,8	81,5	82,1	80,2	80,9	81,6	90,1
Cadreita	-1,6556731	42,26433	79,7	80,5	79,7	79,1	78,3	81,2	76,7	80,1	79,2	76,4	79,0	79,1	82,6
Bardenas Reales	-1,7183027	42,207768	81,7	82,1	80,8	80,0	79,4	81,4	79,3	80,9	80,6	78,1	81,1	80,5	79,3
Sartaguda	-2,0512344	42,361948	82,1	81,9	80,8	80,0	79,7	81,4	80,6	81,3	80,8	78,5	81,0	80,7	78,2
Olite	-1,662579	42,423779	81,3	81,4	80,5	80,8	79,7	82,0	78,8	81,3	80,9	78,1	80,2	80,5	85,7
Murillo el Cuende	-1,6153521	42,361474	81,2	81,3	80,8	80,6	79,5	81,9	78,6	81,1	80,6	77,8	79,8	80,3	82,4
Corella	-1,8398436	42,115577	81,7	81,7	80,4	77,9	79,7	80,4	79,3	80,3	80,7	78,5	79,2	80,0	81,2
Funes	-1,8066789	42,287885	81,2	81,7	80,1	79,6	78,8	81,0	79,0	79,8	80,7	77,7	80,5	80,0	79,8
Lerin	-1,9763006	42,503595	82,5	82,2	80,8	81,7	80,7	81,6	81,0	81,6	81,9	79,5	80,9	81,3	87,3
Los Palacios y Villafranca	-5,9390554	37,179127	46,3	38,8	49,4	36,7	41,1	50,5	42,3	42,3	44,6	39,3	53,9	44,1	56,8
Las cabezas de San Juan	-5,884722	37,01556	45,6	38,7	47,9	36,6	40,4	49,2	41,2	43,2	44,1	38,1	51,8	43,3	59,2
Lebrija	-6,1261602	36,976641	43,4	36,6	47,1	34,7	39,3	48,9	41,3	41,0	42,2	36,4	50,8	42,0	63,1
Aznalcazar	-6,2733503	37,151795	48,4	40,9	53,0	37,7	45,6	56,4	47,9	46,0	46,5	39,9	54,9	47,0	62,9
Isla Mayor	-6,1512787	37,098521	45,6	37,9	47,8	37,3	41,1	50,5	42,0	43,2	43,4	38,6	51,3	43,5	58,0
La puebla del Rio	-6,1338321	37,226032	45,6	38,5	48,6	37,2	40,3	50,5	42,9	43,0	44,2	39,0	52,7	43,8	60,7
La puebla del Rio II	-6,0465691	37,080174	45,8	39,0	48,8	37,3	41,8	51,4	42,8	42,9	44,0	39,1	51,7	44,1	62,9
Ecija	-5,0770704	37,592934	55,4	45,7	53,8	51,1	47,3	57,7	48,7	48,5	51,0	45,8	57,0	51,1	66,3
La Luisiana	-5,2281407	37,525293	55,6	47,7	57,3	47,1	48,7	59,0	49,6	52,1	53,6	48,3	60,0	52,6	68,1
Carmona	-5,587615	37,400903	48,9	40,9	50,8	40,1	43,2	53,5	45,4	45,6	46,7	41,3	54,7	46,4	63,8
Osuna	-5,1348377	37,25503	54,2	46,4	55,6	46,2	47,9	58,0	49,8	52,0	52,4	46,5	58,9	51,6	64,9
La Rinconada	-5,924839	37,456832	45,8	37,8	48,1	39,3	39,8	50,1	41,0	42,9	43,8	38,9	53,2	43,7	64,9
Sanlucar La Mayor	-6,2550749	37,42179	52,0	42,7	55,3	40,3	46,8	57,6	50,2	48,7	50,2	42,1	57,7	49,4	66,6
Villanueva del Rio y Minas	-5,6840093	37,613036	48,2	39,9	49,9	40,7	42,8	52,6	44,3	44,2	45,5	40,6	54,6	45,7	59,9
Lora del Rio	-5,5407037	37,660906	53,1	45,0	55,5	44,5	46,6	57,4	48,6	49,6	50,4	45,1	58,8	50,4	65,9
Los Molares	-5,6729697	37,176152	48,4	40,7	50,8	40,3	42,6	52,4	44,6	44,7	46,0	40,7	54,1	45,9	63,9
Guillena	-6,06419	37,514568	48,3	40,9	49,2	39,6	41,6	50,9	43,0	44,6	45,3	40,3	53,6	45,2	64,0
Puebla Cazalla	-5,3509152	37,218131	54,8	47,9	57,0	45,8	48,3	58,3	50,0	52,0	53,6	47,4	59,5	52,2	62,6
Alcala del Rio	-5,9641033	37,512529	46,2	38,0	47,6	36,6	39,5	49,3	40,4	41,7	42,9	37,7	50,9	42,8	56,5
San Javier	-0,819705	37,791664	43,9	40,0	48,1	27,7	42,7	50,7	40,6	40,7	40,8	37,4	46,6	41,7	56,5
Torre Pacheco	-0,8985888	37,773803	42,1	38,7	45,7	26,8	41,4	48,4	39,6	39,0	38,7	35,3	45,8	40,1	63,7
San Javier	-0,8836862	37,848045	45,0	41,4	49,2	29,1	43,4	51,3	41,7	41,1	42,5	38,8	47,7	42,8	57,9
Torre Pacheco	-0,9316281	37,823827	47,1	43,4	50,4	30,3	44,7	52,7	44,0	42,7	43,7	40,0	49,5	44,4	59,6
Torre Pacheco	-0,9867861	37,74765	43,5	39,8	48,0	26,9	41,8	50,1	40,7	39,2	40,8	36,7	47,0	41,3	63,7

Pedralba	-0,7175861	39,567014	65,7	59,7	62,8	53,9	61,2	68,7	58,6	57,3	60,8	55,7	62,8	60,6	69,6
Liria	-0,627062	39,691055	64,9	58,1	60,9	54,5	61,5	67,2	57,1	58,1	60,7	55,7	61,8	60,1	73,5
Benifayo	-0,4618662	39,280627	50,8	48,5	53,8	37,7	50,5	58,8	49,2	46,7	49,7	45,9	54,5	49,7	60,1
Cheste	-0,7444395	39,518889	70,0	65,3	67,4	58,5	67,1	73,1	63,6	64,1	66,2	61,3	66,5	65,7	67,3
Tabernes de Valldigna	-0,2380292	39,095261	54,2	52,9	57,2	40,5	53,6	62,4	51,6	49,5	53,3	49,4	56,5	52,8	53,4
Villanueva de Castellon	-0,5242892	39,065567	53,4	51,3	57,1	41,2	52,6	62,0	52,2	48,9	53,1	49,0	56,7	52,5	64,4
Sagunto	-0,2663216	39,647534	55,0	53,1	57,4	42,3	55,1	62,9	52,2	51,4	54,2	51,0	57,9	53,9	63,2
Benavites	-0,2162186	39,730391	58,3	56,1	60,6	44,8	56,8	66,7	55,6	53,4	56,8	53,5	59,6	56,5	61,1
Moncada	-0,3989602	39,587729	56,3	54,5	58,9	43,6	55,7	64,0	53,6	51,6	55,6	52,2	58,7	55,0	64,9
Carcagente	-0,4461657	39,113604	51,8	50,2	55,2	39,5	52,3	58,9	49,9	48,4	51,6	47,4	55,6	51,0	65,5
Carlet	-0,5459462	39,22642	62,1	53,9	59,7	47,4	57,2	65,4	54,2	53,9	56,6	51,3	60,3	56,5	78,8
Luchente	-0,3600825	38,938508	67,1	66,3	69,0	56,7	66,1	73,8	63,9	63,9	66,6	61,5	67,5	65,7	89,7
Requena	-1,2323883	39,504667	78,1	77,9	77,1	77,0	77,1	79,4	76,3	76,1	76,5	73,8	75,5	76,8	62,1
Algemesi	-0,4353656	39,216442	50,4	48,9	54,6	36,8	51,0	58,3	49,0	47,0	50,4	46,8	54,3	49,8	66,7
Campo Arcis	-1,1622154	39,433357	77,5	76,8	76,1	75,2	75,8	79,1	75,3	74,2	75,0	72,8	74,5	75,7	64,2
Betera	-0,4685258	39,597708	57,7	55,9	60,7	46,3	56,8	65,8	55,5	53,2	56,5	53,1	60,2	56,5	68,2
Picasent	-0,4976324	39,362484	56,4	53,9	59,1	43,3	56,1	64,7	53,9	52,1	55,5	51,7	59,0	55,1	66,6
Montesa	-0,6383798	38,954502	69,1	64,3	66,8	57,9	65,0	71,5	62,2	62,8	65,2	60,1	65,9	64,6	57,5
Jativa	-0,5497109	38,998803	56,5	54,7	59,6	45,2	56,6	64,2	54,2	52,5	56,8	53,3	59,4	55,7	83,4
Villalonga	-0,2042579	38,892111	56,1	54,0	59,7	44,3	56,6	64,6	53,4	52,0	55,8	52,5	58,8	55,3	61,9
Gandia	-0,2506841	38,964297	60,6	58,5	62,7	49,2	59,3	67,9	56,9	55,9	59,3	55,5	62,1	58,9	60,7
Bolbaite	-0,6901658	39,069153	69,6	65,0	67,6	58,2	65,9	72,9	63,4	63,6	66,4	60,9	66,1	65,4	70,2
Chulilla	-0,8322414	39,67678	69,5	64,8	67,5	59,8	66,0	72,4	63,3	63,9	66,4	60,9	66,8	65,6	73,1
Almoacid de la Sierra	-1,3299642	41,452078	78,1	76,9	78,0	76,2	74,6	78,5	73,9	74,8	75,7	72,3	74,6	75,8	83,0
Belchite	-0,7216155	41,350306	77,4	75,8	76,4	75,5	72,3	78,3	71,6	74,4	75,0	71,5	73,6	74,7	85,1
Quinto	-0,5186373	41,388348	71,6	67,9	69,3	76,0	66,2	75,7	65,0	65,6	68,8	64,4	69,8	69,1	81,7
Fabara	0,1540344	41,167877	72,3	69,5	70,8	74,5	67,4	76,8	65,5	66,9	69,6	64,8	69,6	69,8	81,1
Epila	-1,2820466	41,583234	77,2	74,6	75,9	73,6	72,7	78,2	72,3	73,0	73,8	70,1	74,0	74,1	83,5
Ejea de los Caballeros	-1,1961298	42,097715	79,4	78,0	78,7	79,2	76,4	80,0	77,3	77,8	78,3	76,0	76,9	78,0	85,8
Sabada	-1,309387	42,267312	81,0	81,5	79,8	80,0	79,6	81,9	77,9	80,6	80,3	77,7	79,9	80,0	90,3
Luna	-0,9359498	42,095761	80,3	78,9	79,6	80,4	77,1	80,2	78,1	78,9	79,5	77,4	77,6	78,9	89,1
Santa Engracia	-1,3305062	41,921246	77,4	75,1	76,0	76,0	73,4	78,1	73,8	73,7	75,6	72,3	74,7	75,1	85,7
Pastriz	-0,7461599	41,59514	77,7	76,3	76,4	76,1	72,5	78,4	71,2	74,4	74,7	71,3	74,4	74,9	81,1

Zaragoza	-0,823819	41,71363	78,3	76,5	76,2	77,5	73,9	78,6	73,5	75,6	76,2	73,1	75,1	75,9	80,4
Calatayud	-1,6583593	41,332167	79,2	78,9	78,4	79,1	77,8	78,4	78,3	78,4	78,5	76,4	76,3	78,2	87,5
Borja	-1,5076903	41,855146	78,7	78,0	78,0	77,5	74,8	79,5	75,3	76,4	77,6	73,5	75,7	76,8	86,2
Tarazona	-1,7458841	41,916177	82,8	82,4	81,2	80,4	81,9	82,3	81,3	82,0	82,1	80,0	80,7	81,5	84,8
Caspe	-0,0710973	41,303968	69,3	66,8	70,1	73,2	64,2	75,3	62,6	63,9	66,6	62,0	67,0	67,4	80,9
Osera de Ebro	-0,5363755	41,545081	74,4	72,1	73,5	76,1	69,4	78,6	68,6	68,4	71,5	67,2	71,4	71,9	81,1
Daroca	-1,4247095	41,108073	78,7	78,8	77,9	79,3	78,3	77,8	79,2	77,9	78,9	77,6	76,9	78,3	86,7
Zuera	-0,7511391	41,869389	78,2	76,9	76,4	78,1	74,7	78,8	75,1	76,5	76,8	74,4	75,2	76,5	84,8
El Bayo	-1,2487734	42,175713	79,5	78,4	78,8	79,0	76,5	80,4	77,5	78,0	78,3	76,2	77,3	78,2	86,4
Tauste	-1,1428386	42,00023	77,7	75,5	75,9	76,4	73,3	78,4	73,8	73,9	75,7	71,9	74,6	75,2	85,9
Boquiñeni	-1,2496818	41,843217	78,4	75,9	76,3	76,4	73,4	78,5	73,0	74,0	75,5	71,6	74,5	75,2	84,7
