# Supplementary material for: Agroclimatic Metrics for the Main Stone Fruit Producing Areas in Spain in Current and Future Climate Change Scenarios: Implications From an Adaptive Point of View
Source: Front Plant Sci. 2022 Jun 8;13:842628. doi: 10.3389/fpls.2022.842628 (PMC9213681; doi:10.3389/fpls.2022.842628)
Supplement: Supplementary file 2 [file Data_Sheet_2.PDF]

Supplementary Table 2. Mean accumulated portions (1st November - end February) for 2025-2045, RCP 8,5 at each location.
The last column shows the chilling accumulation for the current situation, for comparison purposes
M1: bcc-csm1-1-m; **M2:** BNU-ESM; **M3:** CanESM2; **M4:** CMCC-CM; **M5:** GFDL-ESM2G; **M6:** inmcm4
M7: IPSL-CM5A-LR; **M8:** MIROC-ESM; **M9:** MPI-ESM-LR; **M10:** MPI-ESM-MR; **M11:** MRI-CGCM3

Municipality	Longitude	Latitude	M1	M2	M3	M4	M5	M6	M7	M8	M9	M10	M11	MEAN	CURRENT
Campo de Mirra	-0,7729762	38,679366	76,3	77,1	75,3	72,2	76,3	78,6	75,1	73,4	74,4	72,3	74,1	75,0	81,4
Villajoyosa	-0,2561866	38,527917	42,1	41,2	43,9	27,3	48,3	52,7	42,2	37,8	45,4	44,1	49,2	43,1	52,9
Ondara	0,0065631	38,818581	44,6	44,5	45,9	30,4	50,7	57,3	45,8	40,2	48,7	47,1	52,3	46,1	56,1
Denia Gata	0,082579	38,792724	47,3	46,7	49,3	33,7	52,5	58,8	48,5	44,4	50,2	50,1	53,8	48,7	61,2
Pinoso	-1,060721	38,427413	74,5	74,7	74,3	69,2	74,8	77,9	73,2	72,0	72,7	70,6	74,0	73,5	81,6
Monforte del Cid	-0,7303963	38,398862	52,1	51,6	53,5	38,5	55,2	59,3	51,0	47,2	52,1	49,3	56,7	51,5	64,4
Crevillente	-0,7831581	38,240831	42,9	41,3	43,8	29,0	46,5	51,8	42,0	39,2	43,6	40,9	49,2	42,7	53,2
Almoradi	-0,7745396	38,031431	42,7	40,3	46,5	28,2	47,1	51,5	43,0	38,8	44,2	41,7	48,9	43,0	55,6
Callosa de Sarria	-0,1044988	38,650249	57,3	57,8	59,1	46,0	61,5	67,0	57,5	54,7	59,7	58,1	61,0	58,2	49,7
Pilar de la Horadada	-0,8125284	37,868588	39,0	36,6	41,2	23,8	43,3	48,2	38,8	34,8	40,1	37,9	45,3	39,0	55,7
Catral	-0,8055704	38,153124	42,2	40,1	44,3	26,7	45,6	50,3	41,1	37,5	42,8	40,7	47,9	41,8	56,3
Altea	-0,0795078	38,603643	48,1	48,2	50,9	35,3	54,8	60,2	49,5	46,0	51,7	51,0	54,6	50,0	50,2
Planes	-0,3529076	38,78476	69,2	69,5	69,9	61,1	71,1	75,8	68,5	66,3	69,1	68,3	69,8	69,0	72,1
Villena	-0,8753684	38,595491	66,5	66,9	66,1	58,4	67,8	71,2	64,9	62,7	65,3	63,0	67,9	65,5	79,1
Agost	-0,6498214	38,421512	56,2	54,9	57,3	43,2	58,8	63,2	54,8	51,9	56,4	54,4	59,7	55,5	65,2
Almansa	-1,1075837	38,903228	78,4	79,1	78,1	76,3	78,7	79,7	77,8	76,8	77,2	75,6	76,3	77,6	80,2
Ontur	-1,4957688	38,622866	70,4	70,8	69,9	63,0	71,6	74,3	69,0	66,7	69,2	67,1	71,1	69,4	82,1
Caudete	-0,9798818	38,734665	73,5	73,4	73,7	67,1	73,4	76,7	71,7	70,3	71,4	70,2	72,4	72,2	75,4
La Mojonera	-2,7043824	36,787318	45,1	46,3	45,1	30,4	46,8	50,8	41,4	44,9	43,9	41,7	50,1	44,2	44,0
Almeria	-2,4024534	36,835404	28,5	28,1	29,5	13,6	31,0	33,6	26,2	27,3	28,4	26,9	33,2	27,8	38,6
Nijar	-2,1580794	36,95057	39,9	39,5	40,3	24,4	41,0	46,1	36,8	39,2	39,5	37,4	45,1	39,0	62,7
Tabernas	-2,3023755	37,091315	58,7	60,2	58,8	48,5	59,0	64,0	54,8	57,5	56,3	54,3	61,5	57,6	71,1
Fiñana	-2,8388277	37,156718	74,3	75,7	74,5	74,1	74,4	76,2	72,9	74,2	73,1	71,0	72,9	73,9	86,1
Cuevas de Almanzora	-1,7704017	37,389125	46,3	47,3	44,9	31,4	47,1	52,6	42,3	46,2	44,8	42,9	50,2	45,1	64,7
Huercal-overa	-1,8842832	37,412428	52,9	54,8	52,3	40,8	53,8	59,2	49,5	52,2	51,8	49,1	56,9	52,1	64,4
Cuevas de Almanzora	-1,800522	37,256757	34,5	34,1	33,9	18,6	36,4	40,4	32,1	34,1	34,1	31,9	40,3	33,7	50,3
Adra	-2,9923491	36,746758	34,8	31,6	32,1	24,9	36,6	41,9	32,4	30,4	34,2	32,8	40,4	33,8	41,2
Tijola	-2,457021	37,371918	66,9	69,5	66,6	60,0	66,9	70,4	62,9	67,1	64,4	61,6	67,7	65,8	81,0

Totana	-1,5130934	37,732459	50,8	49,5	52,4	37,7	53,2	58,6	49,5	46,3	50,6	48,1	55,5	50,2	70,8
Alhama	-1,4167602	37,7922	50,6	49,4	52,5	36,5	53,5	58,6	49,3	46,1	50,8	48,4	55,5	50,1	65,8
Librilla	-1,3382889	37,899373	55,4	54,2	55,9	42,3	57,9	61,7	54,2	51,9	54,3	51,7	58,9	54,4	64,9
Mazarron	-1,4009916	37,56215	42,7	43,8	46,0	26,6	47,7	52,3	42,9	41,8	45,5	43,5	48,6	43,8	49,4
Mazarron	-1,3788416	37,614572	45,4	46,3	48,4	30,5	49,7	53,8	44,7	44,1	47,8	45,5	50,7	46,1	58,6
Zalamea de la Serena	-5,6910276	38,678704	69,5	63,6	68,5	61,6	67,1	72,1	67,3	63,5	68,1	64,5	71,7	67,0	78,2
Monterrubio de la Serena	-5,3836134	38,591582	72,3	67,2	71,1	64,4	69,4	73,3	68,8	67,7	70,2	66,4	73,2	69,5	83,3
Don Benito	-5,9062469	38,930491	63,8	58,2	62,9	55,9	63,4	66,8	63,8	57,7	64,9	60,3	68,2	62,4	75,4
Villagonzalo	-6,1858738	38,837247	66,6	56,5	64,0	60,5	63,5	68,7	66,5	58,3	64,8	60,4	71,9	63,8	73,8
Jerez de los Caballeros	-6,7369024	38,281336	65,7	56,1	63,0	52,9	61,7	68,0	64,1	57,6	62,6	58,7	67,8	61,7	75,3
Olivenza	-7,0578251	38,720921	66,8	55,2	62,7	53,7	60,6	68,8	63,4	56,5	62,4	57,9	69,0	61,6	73,5
Villafranca de los Barros	-6,3485695	38,575591	68,5	59,3	66,4	58,0	63,7	70,8	66,7	60,6	64,9	61,4	70,4	64,6	74,7
Merida	-6,3192869	38,845149	66,5	57,2	64,7	60,1	64,1	69,3	66,6	58,4	65,0	60,6	72,1	64,1	75,7
Azuaga	-5,7077922	38,391445	73,5	68,3	72,9	66,1	70,3	74,6	70,5	68,6	71,8	68,4	74,3	70,9	81,2
Puebla de alcocer	-5,0955855	39,074643	71,9	68,3	71,4	65,7	71,5	74,0	71,2	68,7	71,6	67,5	73,8	70,5	78,9
Don Benito	-5,858992	38,984723	63,8	57,1	62,5	56,1	62,2	67,2	63,2	56,8	65,3	60,2	68,6	62,1	74,0
Badajoz	-6,827838	38,877039	65,5	55,3	63,3	58,8	62,3	67,9	65,2	56,9	64,1	59,3	71,0	62,7	73,4
Pueblonuevo del Guadiana	-6,7328012	38,912998	65,4	55,2	61,9	58,8	62,2	68,2	64,8	56,4	63,7	58,9	70,6	62,4	73,3
Lobon	-6,6655535	38,860185	66,1	56,0	62,4	58,5	62,6	66,5	65,6	57,3	63,2	58,8	71,3	62,6	72,7
Arroyo de San Servan	-6,4728164	38,858246	67,4	58,0	64,2	60,8	65,0	69,4	66,9	59,2	65,4	61,2	71,9	64,5	74,4
Villar de Reina	-5,742601	39,102295	67,7	62,9	66,8	60,9	66,6	70,5	66,9	62,7	68,7	64,0	71,6	66,3	78,9
Cartagena	-0,9508754	37,688833	41,7	42,8	44,4	25,9	46,1	50,9	41,1	41,7	43,7	42,3	46,6	42,5	58,8
Murcia	-1,1227711	37,831265	50,7	50,3	51,9	36,5	55,1	58,6	50,0	48,0	51,7	49,4	55,5	50,7	62,8
Fuente alamo	-1,1292626	37,748269	40,9	39,2	44,0	26,3	45,2	50,2	40,9	36,7	42,2	40,2	47,1	41,2	63,7
Cartagena	-1,070786	37,676671	41,1	39,1	43,1	25,6	45,2	50,1	40,6	37,1	41,7	39,4	47,3	40,9	60,0
Cartagena	-0,8037931	37,611152	41,8	42,7	44,2	27,4	47,1	51,6	41,5	41,2	45,4	43,8	47,5	43,1	58,7
Fuente alamo	-1,2380371	37,699008	49,4	47,0	50,6	34,6	52,3	56,7	48,1	44,7	49,0	46,9	53,8	48,5	63,2
Casatejada	-5,6781	39,867824	69,8	64,1	68,1	63,6	69,0	72,0	68,4	65,3	70,1	65,8	74,0	68,2	81,3
Aldehuela del Jerte	-6,2302346	40,008316	69,4	62,7	68,2	64,8	65,5	69,7	69,8	65,1	68,3	63,1	75,3	67,4	79,5
Moraleja	-6,6759606	40,046357	68,2	61,1	67,7	62,8	65,7	70,4	69,5	59,2	67,9	63,0	72,6	66,2	80,0
Coria	-6,5458096	39,978094	69,1	62,0	67,9	64,6	66,1	70,6	69,6	63,8	69,3	63,9	75,4	67,5	78,4
Madrigalejo	-5,5954391	39,135847	68,4	62,8	66,6	61,1	68,1	72,0	67,9	62,7	68,5	64,2	72,2	66,8	80,7
Valdesalor	-6,4785825	39,377191	70,0	62,7	68,2	63,2	67,9	73,5	69,8	62,3	68,3	64,5	73,3	67,6	80,3

Peraleda de la Mata	-5,4639595	39,861132	71,9	66,3	70,6	66,0	71,2	74,7	71,6	68,0	72,4	68,3	74,2	70,5	82,4
Tejeda de tietar	-5,8600359	39,960042	72,9	73,3	72,9	67,6	73,5	74,5	74,3	74,4	73,4	68,9	77,3	73,0	77,6
Casar de Palomero	-6,3056933	40,298554	79,0	76,6	78,9	76,5	77,7	80,3	78,8	74,9	77,5	73,5	80,1	77,6	84,5
Madroñera	-5,7623097	39,464885	79,0	75,5	77,8	73,6	77,9	80,6	77,5	74,9	77,9	74,3	78,9	77,1	84,2
Guadalupe	-5,3482094	39,387141	77,8	74,6	77,0	72,7	76,6	79,2	76,4	74,1	77,1	72,8	78,6	76,1	85,3
Alcantara	-6,8981123	39,746563	67,6	57,6	64,4	61,2	64,8	69,5	68,7	58,4	65,8	61,6	72,8	64,7	78,5
Jarandilla de la Vega	-5,6463392	40,101413	80,5	80,7	80,2	77,9	80,7	80,4	81,1	80,2	79,5	76,3	80,7	79,8	84,0
Gargantilla	-5,9414268	40,239041	81,5	81,9	81,4	79,5	80,9	82,0	81,1	80,0	79,7	76,4	80,7	80,5	84,8
Talayuela	-5,5642306	40,011198	73,7	72,9	73,5	67,7	73,2	74,7	73,9	73,4	73,3	68,6	76,7	72,9	81,1
Valdastillas	-5,8687982	40,141215	82,4	82,9	83,2	81,6	81,6	82,7	81,9	80,0	80,8	79,0	80,5	81,5	83,5
Cieza	-1,3097249	38,235442	65,8	65,4	65,8	56,4	67,5	71,5	64,5	63,1	64,1	62,0	67,6	64,9	64,4
Ulea	-1,2578423	38,191392	57,9	57,7	58,1	44,5	60,8	64,7	56,3	53,9	56,8	54,3	61,4	56,9	71,6
Cieza	-1,4963438	38,283888	59,7	58,3	59,3	47,7	61,3	65,1	57,4	55,4	57,6	54,9	62,2	58,1	61,1
Calasparra	-1,6947638	38,253487	60,2	58,9	60,8	47,9	61,5	65,6	58,3	55,2	57,7	55,2	63,3	58,6	72,8
Calasparra	-1,5850018	38,269499	57,6	56,5	57,2	44,9	59,5	63,1	55,0	52,6	55,6	52,7	60,9	56,0	73,3
Caravaca	-1,980057	38,043911	74,4	74,2	74,5	65,7	74,7	75,9	72,9	72,6	72,4	71,1	71,9	72,8	88,1
Cehegin	-1,6828994	38,110901	67,8	67,7	67,4	58,1	68,3	72,1	66,1	63,7	65,6	63,8	68,7	66,3	73,0
Moratalla	-1,813186	38,196653	69,9	69,8	71,1	58,2	70,9	73,3	68,6	68,0	68,7	66,7	68,1	68,5	79,0
Cehegin	-1,7798922	38,104477	71,5	70,8	72,2	60,5	72,3	74,5	70,2	69,7	69,7	68,2	69,3	69,9	76,3
Moratalla	-2,0961142	38,1145	79,3	80,6	78,9	77,8	79,0	79,5	77,8	79,3	77,2	76,6	76,4	78,4	87,1
Vall de Uxo	-0,2304536	39,795861	52,9	52,5	53,9	41,0	57,9	64,8	53,6	48,9	56,5	54,5	58,7	54,1	64,2
Onda	-0,2444114	39,954016	59,4	60,8	57,5	50,0	62,6	68,0	58,3	51,2	60,1	57,9	60,5	58,8	69,2
San Rafael del Rio	0,3675272	40,594077	59,8	61,6	58,8	52,4	62,9	69,6	58,7	50,9	60,8	57,5	62,5	59,6	75,5
Benicarlo	0,4014538	40,411511	52,7	54,3	51,4	43,4	57,4	63,4	52,2	43,8	54,9	51,8	57,5	53,0	67,6
Castellon	-0,1191495	39,989342	57,5	59,0	55,0	48,9	60,7	67,3	56,2	50,0	58,2	55,1	60,6	57,1	70,3
Burriana	-0,1057138	39,887849	48,3	48,4	49,8	35,0	54,4	59,7	50,1	44,3	52,2	49,8	55,0	49,8	66,9
Ribera de Cabanes	0,1464314	40,133934	58,6	60,3	56,7	49,7	62,2	68,7	56,8	50,6	60,2	57,4	60,9	58,4	67,4
Nules	-0,1683946	39,877237	50,9	51,0	52,7	38,4	57,4	62,1	52,0	47,3	55,1	53,4	56,9	52,5	65,4
Segorbe	-0,4830876	39,817295	66,6	67,7	67,1	57,9	70,2	73,9	67,2	62,8	68,1	66,1	68,4	66,9	74,4
Baza	-2,7677154	37,564477	66,8	70,5	66,9	59,9	65,9	70,3	62,9	66,1	63,6	60,7	68,4	65,6	83,9
Puebla de Don Fadrique	-2,3817176	37,876115	77,5	78,5	77,1	74,1	77,8	78,8	76,6	77,0	75,7	74,8	74,3	76,6	82,3
Loja	-4,138128	37,1693	65,5	62,3	66,1	63,3	66,0	70,8	64,6	63,5	66,3	63,0	68,9	65,5	79,5
Iznalloz	-3,5514591	37,416406	69,0	69,2	67,1	66,6	67,3	72,1	66,2	66,6	65,8	63,9	69,3	67,5	86,0

Jerez del Marquesado	-3,1498644	37,190536	73,2	74,6	72,9	72,4	73,0	74,9	71,9	72,3	71,9	70,0	71,4	72,6		84,3
Cadiar	-3,183988	36,923123	69,3	69,5	67,9	67,0	69,1	72,1	66,9	68,2	66,8	65,1	68,8	68,3		81,4
Zafarraya	-4,1538389	36,990314	77,8	78,7	78,6	78,8	78,3	80,4	77,4	78,1	77,6	75,7	78,0	78,1		89,9
Padul	-3,600317	37,018743	68,5	68,7	66,6	66,1	67,3	72,3	66,2	67,1	66,1	64,4	69,6	67,5		80,0
Granada	-3,6385645	37,172054	66,8	65,7	64,3	62,1	65,2	71,4	63,7	64,1	63,9	61,7	67,0	65,1		77,9
Almuñecar	-3,6790578	36,751942	51,4	48,5	48,1	44,2	51,1	57,8	47,9	48,3	50,2	47,8	54,9	50,0		54,1
Gibraleon	-7,0278022	37,318328	41,1	34,2	43,1	27,6	40,0	49,8	44,6	37,3	39,4	36,1	49,2	40,2		59,5
Lepe	-7,2430825	37,302685	43,4	36,6	43,9	29,9	42,2	52,6	42,8	38,1	41,2	38,3	49,6	41,7		57,0
Gibraleon	-7,059841	37,412354	47,5	42,3	48,5	34,1	45,8	55,7	49,0	43,4	44,7	42,2	53,8	46,1		66,0
Moguer	-6,7925285	37,14648	44,7	37,5	44,7	30,3	42,9	53,0	44,7	38,9	42,7	39,4	51,2	42,7		62,3
Niebla	-6,7353478	37,347125	45,3	39,1	46,3	31,7	43,2	53,1	46,6	40,6	44,3	40,3	52,8	43,9		62,5
Aroche	-6,9449915	37,958077	67,2	58,9	64,5	54,9	63,0	69,9	65,2	59,4	63,3	59,8	69,3	63,2		71,4
La puebla de Guzman	-7,2483655	37,552176	56,0	49,0	56,7	42,7	53,7	62,7	56,1	51,2	53,4	50,0	60,0	53,8		69,5
El Campillo	-6,5992719	37,660989	56,3	51,4	57,6	44,1	54,9	62,5	57,7	52,5	53,6	51,1	61,2	54,8		74,5
La Palma del Condado	-6,5415566	37,366968	52,2	44,5	52,8	38,0	50,0	59,4	53,1	47,8	49,1	46,4	58,0	50,1		66,3
Almonte	-6,4765444	37,148345	42,6	35,2	43,4	28,9	40,5	51,2	43,4	37,6	40,8	38,2	49,9	41,1		60,1
Valfarta	-0,1478858	41,531503	73,7	73,2	71,4	76,0	72,7	79,1	72,0	67,0	72,5	69,7	73,6	72,8		83,5
Zaidin	0,2890014	41,637169	72,2	70,8	69,6	75,4	71,2	77,1	70,8	66,4	70,7	68,0	72,4	71,3		79,4
Alcolea de Cinca	0,0731411	41,74095	73,0	72,2	70,0	76,0	71,4	77,1	71,3	67,2	71,6	69,5	72,6	72,0		82,2
Tanarite de Litera	0,3771357	41,780947	73,4	73,7	72,0	75,4	72,6	77,1	71,8	68,7	72,1	69,8	71,7	72,6		81,9
Lanaja	-0,337846	41,786429	76,0	75,4	72,8	78,1	74,3	80,1	74,5	68,8	74,1	72,2	74,7	74,6		83,5
Monzon	0,1273494	41,957687	75,3	75,1	74,0	77,2	74,9	78,3	74,4	71,2	73,4	71,5	74,1	74,5		83,1
Barbastro	0,1126102	42,013471	75,9	76,4	74,1	77,3	74,6	79,1	74,9	71,2	73,6	72,0	74,1	74,8		84,0
Sariñena	-0,1766614	41,771411	74,5	74,0	72,0	76,6	72,8	78,8	72,3	68,5	72,3	70,0	73,1	73,2		83,1
Huesca	-0,3777068	42,105429	77,1	77,1	76,0	78,6	75,7	80,3	76,0	73,0	74,9	73,5	74,6	76,1		83,6
Candasnos	0,094436	41,45994	70,7	70,5	69,0	74,5	70,2	76,0	69,2	65,4	69,6	67,2	71,3	70,3		83,7
Grañen	-0,3560041	41,942469	77,1	76,3	74,7	78,2	74,5	79,5	75,5	71,5	74,4	72,4	74,1	75,3		82,9
Huerto	-0,1365362	41,966019	77,3	77,4	75,6	78,5	75,5	80,3	76,5	71,7	75,0	73,2	75,8	76,1		83,6
Gurrea de Gallego	-0,7311994	41,992829	78,6	78,2	77,0	78,2	77,2	79,2	76,6	74,6	76,9	74,9	74,8	76,9		85,9
Alfantega	0,1477817	41,821958	75,8	75,6	74,1	76,8	74,0	78,4	74,5	70,9	73,3	71,5	73,9	74,4		82,1
Fraga	0,3539314	41,495165	69,4	69,6	66,4	73,5	68,7	75,6	68,3	63,2	68,8	66,5	69,9	69,1		76,9
Tardienta	-0,5075831	41,969367	75,7	75,9	73,8	78,6	74,3	80,5	74,8	69,4	74,2	72,3	74,2	74,9		83,5
San Esteban de Litera	0,3042037	41,882938	76,3	76,3	75,1	77,9	74,8	78,6	75,0	71,7	74,0	72,4	74,3	75,1		82,0

Belver de Cinca	0,2318291	41,742536	74,4	73,3	70,7	76,8	72,6	78,3	72,5	68,4	73,0	70,1	73,3	73,0	83,8
Alberuela de Tubo	-0,2573084	41,883957	74,6	74,1	72,1	76,4	73,1	78,7	72,5	68,8	72,5	70,1	73,7	73,3	84,5
Jumilla	-1,4232837	38,394834	68,0	68,4	67,0	59,5	69,7	73,1	67,0	65,1	66,9	64,4	69,3	67,1	77,7
Yecla	-1,1859032	38,658948	72,9	73,2	72,0	66,9	73,8	76,2	71,9	70,3	70,9	69,3	72,3	71,8	85,6
Yecla	-1,1125211	38,562731	69,5	70,1	69,2	62,1	70,5	74,2	68,5	66,2	68,0	65,7	69,7	68,5	83,7
Jumilla	-1,2407841	38,392588	69,8	70,4	69,6	62,5	71,1	74,9	68,3	66,7	68,0	66,3	70,6	68,9	73,6
Jumilla	-1,3242866	38,31972	62,4	61,7	62,2	52,1	64,3	68,1	60,5	58,8	60,8	58,2	64,7	61,3	74,7
Aitona	0,4609093	41,486913	69,5	68,3	64,6	72,8	68,7	76,0	67,4	62,0	68,0	65,0	70,1	68,4	77,7
Albesa	0,6705502	41,760356	70,7	73,7	70,6	75,3	70,4	76,4	72,1	66,5	70,9	68,7	73,8	71,7	82,6
Alcarras	0,5506119	41,56508	71,9	71,9	70,2	73,7	71,2	77,2	70,0	66,8	70,6	67,5	72,1	71,2	81,5
Alfarras	0,5780224	41,819488	72,1	75,0	71,8	76,7	71,6	76,8	73,1	68,0	71,6	70,8	73,7	72,8	83,0
Algerri	0,6483717	41,801036	72,8	75,7	72,5	77,4	72,7	77,4	74,3	69,7	72,8	71,4	74,7	73,8	80,0
Alguaire	0,5361346	41,742812	75,6	75,2	74,5	77,5	74,1	78,0	73,7	71,2	74,3	71,5	73,3	74,5	84,8
Castellnou de Seana	0,9520619	41,65659	70,4	74,0	71,3	75,9	70,9	77,0	71,8	67,7	71,5	69,5	73,9	72,2	82,6
Cervera	1,2967772	41,662217	77,2	78,9	77,4	81,3	76,6	80,1	77,2	74,7	75,9	74,9	76,2	77,3	83,6
Gimenells i el Pla de la Font	0,3933398	41,658132	74,4	74,0	72,2	75,6	72,5	77,8	72,4	68,6	73,3	70,2	73,1	73,1	83,2
Golmes	0,9248038	41,63641	69,3	73,1	69,8	75,0	69,9	76,6	71,3	66,1	71,3	69,4	73,5	71,4	75,0
Raimat	0,4490319	41,683272	75,1	74,9	72,9	77,3	73,9	79,6	73,6	70,0	73,6	71,1	73,7	74,2	83,1
Balaguer- Monasterio Avellanas	0,7613663	41,879114	74,2	77,1	74,5	78,2	73,5	78,3	75,7	71,5	73,4	72,3	74,8	74,9	83,3
El Canos	1,2041447	41,689385	75,9	78,5	75,8	80,4	75,7	80,1	77,1	73,0	75,2	73,9	76,1	76,5	84,6
El Poal	0,8777387	41,672786	69,3	73,1	69,8	74,9	69,9	76,6	71,3	66,2	71,3	69,4	73,5	71,4	81,3
Sant Marti de Riucorb	1,0885432	41,572353	73,3	75,9	74,2	78,2	72,7	78,4	74,4	70,3	73,1	72,1	75,0	74,3	85,1
Seros	0,4279758	41,463784	69,7	69,3	67,3	72,4	68,9	75,9	66,9	63,1	68,1	64,9	69,8	68,8	78,2
Tarrega	1,1626814	41,666945	73,4	75,5	73,8	79,1	72,8	78,2	74,1	70,0	73,4	72,1	74,8	74,3	80,5
Tornabous	1,0451011	41,68834	72,3	74,4	71,6	76,8	71,7	77,5	73,0	68,4	72,3	70,3	74,4	73,0	80,2
Vallfogona de Balaguer	0,8293888	41,784868	71,4	74,0	71,3	76,5	71,6	77,3	73,1	68,5	71,9	69,9	73,8	72,7	82,5
Vilanova de Segria	0,628392	41,714499	68,7	72,4	69,1	75,0	69,3	74,7	70,1	65,9	69,7	68,0	72,8	70,5	81,9
Lorca	-1,6294551	37,601733	56,9	58,3	59,8	44,3	59,9	64,3	55,7	56,9	58,0	55,5	59,6	57,2	72,8
Lorca	-1,6938893	37,50379	53,8	53,8	52,5	41,5	53,6	60,1	49,2	52,2	51,5	48,3	56,1	52,1	73,8
Agoncillo	-2,2904337	42,468182	82,2	82,3	80,5	80,9	81,6	81,5	80,5	79,8	80,4	79,0	80,7	80,9	89,7
Albelda de Iregua	-2,4718558	42,380733	82,1	81,9	80,5	81,5	81,4	81,3	81,5	79,8	80,4	79,2	80,2	80,9	90,3
Asenjo	-2,1533164	42,340952	83,0	83,0	81,3	82,3	81,9	82,0	81,3	80,1	81,3	79,6	81,4	81,6	91,1
Logroño	-2,5136369	42,43969	82,7	83,0	80,6	81,4	81,5	81,9	81,2	80,2	80,9	79,1	80,8	81,2	91,5

Santa Engracia del Juvera	-2,2629377	42,368971	82,5	82,9	80,9	81,4	81,9	81,9	81,1	79,8	80,5	79,1	81,0	81,2	86,3
Aldea Nueva de Ebro	-1,9048679	42,222598	80,6	81,3	79,1	77,8	79,8	80,6	78,8	77,4	78,9	76,3	79,6	79,1	86,4
Alfaro	-1,7776916	42,152119	81,3	81,7	80,1	76,0	80,5	80,3	79,9	78,4	79,8	77,9	78,4	79,5	88,6
Calahorra	-2,001826	42,334834	80,8	81,9	79,7	78,0	80,3	80,9	78,9	78,2	78,9	77,0	80,6	79,6	90,9
Corvera (Cabreton)	-1,8924542	42,006904	81,9	81,4	79,6	78,1	80,2	80,7	79,9	78,9	79,8	78,4	79,4	79,8	86,1
Igea	-1,9937535	42,05775	81,9	81,4	80,7	80,5	80,7	80,8	80,3	79,3	80,6	79,3	79,4	80,5	88,5
Quel	-2,037178	42,252488	81,7	82,3	80,2	79,9	80,9	81,3	80,2	78,7	79,6	77,9	80,7	80,3	85,4
Rincon de Soto	-1,8508464	42,251583	80,6	81,3	79,2	77,8	79,8	80,5	78,8	77,4	79,0	76,3	79,7	79,1	85,8
Aguilas	-1,5921627	37,418665	40,4	40,4	43,2	25,0	44,4	50,4	40,1	38,7	42,7	40,7	45,9	41,1	48,9
Lorca	-1,8177885	37,855634	73,7	73,2	73,7	63,5	75,0	76,1	72,3	72,1	72,4	70,6	71,6	72,2	83,5
Lorca	-1,623984	37,4878	53,7	54,7	56,4	38,9	57,7	62,6	53,2	54,0	55,4	53,5	57,3	54,3	59,9
Puerto Lumbreras	-1,7255508	37,590472	53,3	53,8	52,3	41,7	53,1	59,0	48,3	51,6	51,6	48,7	55,9	51,7	68,9
Mula	-1,4294482	38,065871	59,2	58,3	59,2	46,0	61,6	65,9	57,3	54,7	57,7	55,4	61,6	57,9	61,4
Mula	-1,46674	38,041031	60,9	59,8	61,2	49,1	63,4	67,5	59,1	56,3	59,7	57,5	64,1	59,9	60,6
Torres de Cotillas	-1,3025362	38,006971	49,1	46,5	50,3	33,6	51,6	56,4	46,7	43,2	48,3	45,8	53,9	47,8	64,2
Molina del Segura	-1,2206884	38,127483	50,7	48,5	52,0	36,2	52,7	57,3	48,5	45,3	50,3	47,9	55,6	49,5	62,5
Molina del Segura	-1,2336707	38,071139	48,4	46,2	50,1	33,3	50,7	55,7	47,2	43,6	48,4	45,6	53,4	47,5	64,6
Abanilla	-1,0655079	38,170041	48,2	46,0	50,0	33,3	51,5	56,4	46,8	44,3	48,6	45,9	53,0	47,6	64,5
Fortuna	-1,1526819	38,161028	49,3	47,0	49,6	35,1	51,3	56,5	47,5	45,1	49,1	45,9	54,7	48,3	63,7
Ojos	-1,3394287	38,113316	55,5	54,8	56,5	42,6	58,2	62,7	53,9	51,0	55,5	52,1	59,2	54,7	52,7
Beniel	-0,9997837	38,034507	46,5	44,4	47,6	31,7	49,8	54,4	45,6	42,6	46,7	44,1	51,9	46,0	63,2
Murcia	-1,2682702	37,898166	57,9	57,7	58,4	45,4	61,1	65,5	56,6	54,3	57,2	54,9	61,0	57,3	48,9
Murcia	-0,9840042	37,977528	49,0	47,5	51,1	34,5	52,6	57,6	47,9	44,3	49,4	46,9	54,0	48,6	56,4
Murcia	-1,1347189	37,940075	52,8	51,5	54,0	39,1	55,6	61,1	52,4	49,2	52,8	50,3	57,8	52,4	61,4
Fitero	-1,8426437	42,046077	82,1	81,9	80,3	78,2	80,9	80,7	80,6	79,4	80,3	79,1	80,0	80,3	85,4
Cascante	-1,7239555	42,034371	80,8	81,5	79,1	75,7	80,2	80,5	78,4	77,6	78,7	77,5	78,7	79,0	86,2
Ablitas	-1,6447131	41,996446	77,1	77,4	75,8	75,6	75,3	79,0	74,1	72,5	75,8	72,9	74,3	75,4	85,9
Murillo el fruto	-1,4871859	42,38498	80,3	81,0	80,0	79,4	80,6	81,5	78,6	78,4	78,9	77,3	79,1	79,5	84,5
Miranda de Arga	-1,8087315	42,511252	81,7	82,0	79,6	79,6	81,1	80,9	80,2	78,7	79,8	78,4	80,1	80,2	86,1
Falces	-1,7925482	42,409669	82,7	82,8	80,6	80,4	81,9	82,1	80,7	80,1	80,4	78,4	81,0	81,0	85,8
Bargota	-2,2992201	42,477657	82,2	82,4	80,5	80,9	81,6	81,5	80,5	79,8	80,4	79,0	80,7	80,9	85,7
Bardenas Reales	-1,5187546	42,295154	81,1	81,9	80,3	80,9	81,2	81,6	78,9	79,9	79,5	78,3	80,0	80,3	86,0
Los Arcos	-2,1845206	42,539308	82,6	82,7	80,7	81,5	81,7	81,6	81,7	80,1	80,9	79,6	80,8	81,3	86,9

Sesma	-2,126631	42,473409	83,0	82,7	80,7	81,1	81,6	82,0	81,1	80,2	81,1	79,7	81,1	81,3	90,1
Cadreita	-1,6556731	42,26433	79,3	79,8	78,6	78,0	79,6	80,4	76,7	77,9	78,1	76,1	78,4	78,4	82,6
Bardenas Reales	-1,7183027	42,207768	81,1	82,7	80,0	78,1	80,3	81,0	79,1	78,6	79,3	77,0	80,9	79,8	79,3
Sartaguda	-2,0512344	42,361948	81,3	82,1	80,2	79,4	81,1	80,9	79,8	79,4	79,5	77,8	80,6	80,2	78,2
Olite	-1,662579	42,423779	80,4	81,1	80,2	79,9	80,3	81,6	78,5	79,1	79,6	77,7	79,9	79,9	85,7
Murillo el Cuende	-1,6153521	42,361474	80,4	81,0	79,9	79,9	80,6	81,6	78,4	78,9	79,6	77,7	79,5	79,8	82,4
Corella	-1,8398436	42,115577	81,4	81,7	79,6	76,3	80,5	80,4	79,6	78,7	79,7	78,5	79,2	79,6	81,2
Funes	-1,8066789	42,287885	80,6	81,6	79,4	77,7	79,9	80,4	79,2	77,5	79,1	76,4	80,3	79,3	79,8
Lerin	-1,9763006	42,503595	82,3	82,5	80,4	80,7	81,4	81,5	80,2	79,5	80,4	79,1	80,8	80,8	87,3
Los Palacios y Villafranca	-5,9390554	37,179127	42,8	37,8	45,2	32,5	42,5	50,0	41,8	38,9	43,1	39,6	52,9	42,5	56,8
Las cabezas de San Juan	-5,884722	37,01556	42,6	37,8	43,0	32,2	41,6	48,8	39,8	37,6	42,4	39,1	51,1	41,5	59,2
Lebrija	-6,1261602	36,976641	40,0	36,1	42,2	29,8	40,9	48,2	40,2	36,8	40,6	37,9	49,6	40,2	63,1
Aznalcazar	-6,2733503	37,151795	46,0	39,0	48,1	32,4	44,9	55,8	48,5	42,6	44,4	40,8	53,6	45,1	62,9
Isla Mayor	-6,1512787	37,098521	42,3	37,2	43,3	32,4	41,9	50,2	41,0	38,6	42,2	39,6	51,2	41,8	58,0
La puebla del Rio	-6,1338321	37,226032	42,4	37,5	43,7	32,7	41,7	50,3	41,7	38,2	42,4	39,7	51,6	42,0	60,7
La puebla del Rio II	-6,0465691	37,080174	42,5	38,0	43,2	32,5	43,0	51,2	41,8	38,0	42,9	40,2	50,7	42,2	62,9
Ecija	-5,0770704	37,592934	52,0	45,2	49,4	46,5	50,2	57,3	48,4	44,8	50,0	47,6	55,8	49,7	66,3
La Luisiana	-5,2281407	37,525293	52,4	47,3	54,2	43,2	50,8	58,2	50,3	48,2	51,9	48,1	58,9	51,2	68,1
Carmona	-5,587615	37,400903	46,1	40,5	47,5	35,5	45,3	53,9	44,5	40,7	45,4	42,3	54,4	45,1	63,8
Osuna	-5,1348377	37,25503	50,6	45,3	51,7	41,4	49,2	57,6	49,9	47,5	50,7	47,5	57,6	49,9	64,9
La Rinconada	-5,924839	37,456832	42,1	36,8	44,0	34,7	40,7	49,5	40,7	38,2	42,6	39,1	52,1	41,9	64,9
Sanlucar La Mayor	-6,2550749	37,42179	49,1	42,3	51,2	34,6	46,7	56,9	50,7	45,8	47,2	43,3	56,8	47,7	66,6
Villanueva del Rio y Minas	-5,6840093	37,613036	45,0	40,0	46,9	35,5	44,1	52,5	43,8	40,7	44,8	41,0	53,9	44,4	59,9
Lora del Rio	-5,5407037	37,660906	49,7	44,3	52,2	40,4	48,5	56,9	49,2	46,4	49,9	46,0	57,4	49,2	65,9
Los Molares	-5,6729697	37,176152	45,2	40,4	46,9	35,6	44,5	52,8	44,0	41,1	44,7	41,1	53,2	44,5	63,9
Guillena	-6,06419	37,514568	44,9	39,7	45,4	35,2	43,1	50,9	42,7	39,9	44,0	40,2	52,9	43,6	64,0
Puebla Cazalla	-5,3509152	37,218131	52,0	47,2	52,9	41,6	50,5	58,0	50,1	47,5	51,6	48,3	58,8	50,8	62,6
Alcala del Rio	-5,9641033	37,512529	43,0	36,8	43,4	32,4	40,6	49,2	40,1	37,6	42,4	38,3	50,0	41,3	56,5
San Javier	-0,819705	37,791664	39,3	37,1	42,7	24,4	44,8	48,2	39,1	35,3	41,0	38,9	45,4	39,7	56,5
Torre Pacheco	-0,8985888	37,773803	38,3	35,8	39,5	23,2	42,2	46,9	37,4	34,1	38,9	36,5	44,9	38,0	63,7
San Javier	-0,8836862	37,848045	40,5	37,6	43,5	25,4	44,8	49,2	40,1	35,8	41,5	38,9	46,5	40,3	57,9
Torre Pacheco	-0,9316281	37,823827	41,5	40,0	44,5	26,2	46,6	50,8	41,2	37,8	43,3	40,9	48,4	41,9	59,6
Torre Pacheco	-0,9867861	37,74765	39,2	36,6	42,9	23,7	43,4	48,5	39,4	35,1	40,4	37,3	45,8	39,3	63,7

Pedralba	-0,7175861	39,567014	61,4	57,6	57,5	49,0	62,3	66,8	58,5	52,2	60,1	57,3	62,1	58,6	69,6
Liria	-0,627062	39,691055	60,3	57,5	55,9	50,7	62,5	64,7	58,3	54,0	59,6	57,3	61,2	58,4	73,5
Benifayo	-0,4618662	39,280627	45,7	45,2	47,6	33,9	52,5	58,4	47,8	41,3	49,7	47,3	54,0	47,6	60,1
Cheste	-0,7444395	39,518889	66,6	63,9	62,4	55,0	67,5	70,6	63,6	60,1	64,9	62,8	66,2	64,0	67,3
Tabernes de Valldigna	-0,2380292	39,095261	48,8	49,5	50,6	35,1	55,0	60,9	50,0	44,5	52,4	51,2	56,0	50,4	53,4
Villanueva de Castellon	-0,5242892	39,065567	49,0	48,3	50,7	36,6	54,1	60,2	50,0	43,9	52,4	49,7	55,5	50,0	64,4
Sagunto	-0,2663216	39,647534	49,6	49,8	51,3	37,2	56,1	61,2	51,5	46,4	54,3	52,8	56,6	51,5	63,2
Benavites	-0,2162186	39,730391	53,0	53,3	54,7	39,9	58,6	64,9	54,3	49,6	56,5	54,5	58,9	54,4	61,1
Moncada	-0,3989602	39,587729	51,4	50,9	53,4	38,6	56,8	62,9	52,6	46,6	54,8	53,4	57,7	52,6	64,9
Carcagente	-0,4461657	39,113604	47,0	47,2	48,9	34,2	53,5	58,7	48,6	43,7	51,0	48,9	54,8	48,8	65,5
Carlet	-0,5459462	39,22642	56,7	52,3	52,9	42,0	59,0	63,1	53,8	49,3	56,5	52,8	58,5	54,3	78,8
Luchente	-0,3600825	38,938508	63,6	63,6	64,3	52,7	67,7	72,9	63,7	59,6	65,0	63,3	66,5	63,9	89,7
Requena	-1,2323883	39,504667	77,6	77,8	76,5	74,6	77,8	78,7	76,5	74,4	75,8	74,0	74,7	76,2	62,1
Algemesi	-0,4353656	39,216442	45,4	46,4	47,6	32,5	51,8	57,9	47,7	41,4	50,4	47,9	53,5	47,5	66,7
Campo Arcis	-1,1622154	39,433357	76,9	76,2	75,4	73,3	76,7	78,2	75,3	72,7	74,5	72,4	73,8	75,0	64,2
Betera	-0,4685258	39,597708	53,3	53,5	54,9	41,4	57,8	64,3	54,2	48,5	56,3	54,1	59,2	54,3	68,2
Picasent	-0,4976324	39,362484	50,6	51,3	53,4	38,1	57,4	62,7	52,7	47,1	54,9	53,4	57,5	52,6	66,6
Montesa	-0,6383798	38,954502	65,4	62,2	62,7	53,5	66,3	69,7	63,0	58,4	63,9	61,5	65,1	62,9	57,5
Jativa	-0,5497109	38,998803	51,9	51,3	54,1	39,8	58,3	62,7	53,4	47,4	56,1	54,1	58,5	53,4	83,4
Villalonga	-0,2042579	38,892111	51,1	50,7	53,1	38,7	57,6	62,3	52,1	46,6	55,6	53,7	58,1	52,7	61,9
Gandia	-0,2506841	38,964297	55,7	56,0	57,1	44,8	60,8	66,8	56,2	51,4	58,8	56,7	61,2	56,9	60,7
Bolbaite	-0,6901658	39,069153	65,8	63,8	62,8	54,5	67,3	70,7	63,5	59,2	64,5	62,2	65,3	63,6	70,2
Chulilla	-0,8322414	39,67678	66,4	63,5	62,3	56,6	67,8	70,3	63,2	60,1	64,2	62,8	65,9	63,9	73,1
Almoacid de la Sierra	-1,3299642	41,452078	77,0	77,0	76,4	74,9	75,1	78,3	74,6	72,1	75,1	72,3	73,5	75,1	83,0
Belchite	-0,7216155	41,350306	75,7	75,6	74,4	73,7	74,1	77,2	72,6	70,7	74,8	71,2	72,7	73,9	85,1
Quinto	-0,5186373	41,388348	69,4	67,0	64,6	74,1	67,2	74,4	65,8	61,6	67,8	65,5	70,1	67,9	81,7
Fabara	0,1540344	41,167877	69,4	68,6	65,8	72,7	68,6	75,3	66,8	62,4	68,6	66,0	69,8	68,5	81,1
Epila	-1,2820466	41,583234	75,4	74,3	73,6	71,9	73,5	77,1	72,4	69,4	72,5	70,1	72,2	73,0	83,5
Ejea de los Caballeros	-1,1961298	42,097715	78,8	78,8	77,6	78,5	77,0	79,7	77,0	74,8	77,6	75,6	75,6	77,4	85,8
Sabada	-1,309387	42,267312	80,2	81,3	79,5	79,1	80,2	81,3	78,0	78,6	79,2	77,2	78,8	79,4	90,3
Luna	-0,9359498	42,095761	79,6	79,6	78,9	79,5	78,1	79,7	78,0	76,8	78,6	77,0	77,0	78,4	89,1
Santa Engracia	-1,3305062	41,921246	76,4	75,1	74,0	74,3	75,1	77,0	74,0	70,5	75,3	72,4	73,2	74,3	85,7
Pastriz	-0,7461599	41,59514	76,2	75,8	74,1	74,3	73,9	77,3	72,9	71,3	74,5	71,2	73,0	74,0	81,1

Zaragoza	-0,823819	41,71363	77,1	76,6	75,3	76,0	75,2	77,5	74,3	72,6	75,3	72,7	74,1	75,1	80,4
Calatayud	-1,6583593	41,332167	79,2	78,8	78,9	78,4	78,1	78,5	78,2	76,5	77,1	75,7	75,7	77,7	87,5
Borja	-1,5076903	41,855146	77,3	78,1	76,8	76,1	76,3	79,0	75,5	73,6	76,8	74,1	74,7	76,2	86,2
Tarazona	-1,7458841	41,916177	82,6	82,5	81,1	79,0	81,8	81,7	81,1	80,2	81,1	79,6	80,3	81,0	84,8
Caspe	-0,0710973	41,303968	65,5	65,3	65,5	71,2	65,5	74,3	63,5	59,6	66,6	62,5	67,8	66,1	80,9
Osera de Ebro	-0,5363755	41,545081	72,0	71,4	70,3	75,1	70,1	77,7	69,6	65,0	70,7	67,9	71,5	71,0	81,1
Daroca	-1,4247095	41,108073	79,5	79,3	78,1	79,3	78,3	77,7	78,8	77,1	77,6	77,3	76,8	78,2	86,7
Zuera	-0,7511391	41,869389	77,5	77,3	75,3	76,8	76,1	78,3	74,8	73,5	76,3	73,8	74,1	75,8	84,8
El Bayo	-1,2487734	42,175713	79,3	78,4	78,0	78,4	77,3	80,0	78,1	75,8	77,8	75,8	76,3	77,7	86,4
Tauste	-1,1428386	42,00023	76,6	75,0	74,8	75,1	74,6	77,6	73,9	71,0	74,9	72,1	73,6	74,5	85,9
Boquiñeni	-1,2496818	41,843217	77,0	75,5	74,6	75,0	74,7	77,7	73,7	70,6	74,8	71,8	72,9	74,4	84,7
