# Supplementary material for: Agroclimatic Metrics for the Main Stone Fruit Producing Areas in Spain in Current and Future Climate Change Scenarios: Implications From an Adaptive Point of View
Source: Front Plant Sci. 2022 Jun 8;13:842628. doi: 10.3389/fpls.2022.842628 (PMC9213681; doi:10.3389/fpls.2022.842628)
Supplement: Supplementary file 3 [file Data_Sheet_3.PDF]

**Supplementary Table 3. Mean accumulated portions (1st November - end February) for 2045-2065, RCP 4.5 at each location.**
**The last column shows the chilling accumulation for the current situation, for comparison purposes**

**M1:** bcc-csm1-1-m; **M2:** BNU-ESM; **M3:** CanESM2; **M4:** CMCC-CM; **M5:** GFDL-ESM2G; **M6:** inmcm4
**M7:** IPSL-CM5A-LR; **M8:** MIROC-ESM; **M9:** MPI-ESM-LR; **M10:** MPI-ESM-MR; **M11:** MRI-CGCM3

| Municipality | Longitude | Latitude | M1 | M2 | M3 | M4 | M5 | M6 | M7 | M8 | M9 | M10 | M11 | MEAN | CURRENT |
|---|---|---|---|---|---|---|---|---|---|---|---|---|---|---|---|
| Campo de Mirra | -0,7729762 | 38,679366 | 76,4 | 75,3 | 74,8 | 68,2 | 75,5 | 78,2 | 73,5 | 73,0 | 74,5 | 73,3 | 74,2 | 74,3 | 81,4 |
| Villajoyosa | -0,2561866 | 38,527917 | 39,8 | 39,1 | 40,5 | 22,6 | 45,5 | 53,0 | 40,3 | 37,0 | 44,0 | 43,3 | 45,3 | 40,9 | 52,9 |
| Ondara | 0,0065631 | 38,818581 | 42,4 | 43,0 | 43,6 | 25,6 | 48,6 | 56,5 | 44,6 | 39,3 | 47,0 | 45,9 | 48,9 | 44,1 | 56,1 |
| Denia Gata | 0,082579 | 38,792724 | 46,3 | 45,7 | 47,3 | 28,5 | 49,7 | 57,9 | 46,9 | 44,0 | 49,8 | 49,6 | 50,4 | 46,9 | 61,2 |
| Pinoso | -1,060721 | 38,427413 | 74,2 | 73,6 | 73,1 | 64,8 | 74,0 | 76,9 | 71,1 | 71,4 | 72,6 | 71,7 | 72,9 | 72,4 | 81,6 |
| Monforte del Cid | -0,7303963 | 38,398862 | 50,2 | 48,8 | 51,1 | 32,7 | 52,3 | 58,7 | 49,7 | 46,7 | 52,3 | 49,8 | 53,7 | 49,6 | 64,4 |
| Crevillente | -0,7831581 | 38,240831 | 41,9 | 37,7 | 40,9 | 22,9 | 43,5 | 50,3 | 40,8 | 37,3 | 42,9 | 40,6 | 45,7 | 40,4 | 53,2 |
| Almoradi | -0,7745396 | 38,031431 | 41,3 | 37,2 | 42,6 | 23,0 | 43,7 | 50,9 | 41,3 | 37,5 | 43,6 | 41,3 | 45,4 | 40,7 | 55,6 |
| Callosa de Sarria | -0,1044988 | 38,650249 | 56,1 | 56,1 | 57,6 | 40,2 | 59,3 | 65,8 | 56,1 | 53,7 | 58,7 | 58,2 | 58,8 | 56,4 | 49,7 |
| Pilar de la Horadada | -0,8125284 | 37,868588 | 37,4 | 33,8 | 37,4 | 19,2 | 39,5 | 47,9 | 36,6 | 33,2 | 39,7 | 37,2 | 41,8 | 36,7 | 55,7 |
| Catral | -0,8055704 | 38,153124 | 40,3 | 36,6 | 40,9 | 21,7 | 42,1 | 49,4 | 39,7 | 35,8 | 42,8 | 39,9 | 44,6 | 39,4 | 56,3 |
| Altea | -0,0795078 | 38,603643 | 47,0 | 47,5 | 48,7 | 30,2 | 51,4 | 59,2 | 47,8 | 45,5 | 51,1 | 50,3 | 51,5 | 48,2 | 50,2 |
| Planes | -0,3529076 | 38,78476 | 68,9 | 68,0 | 68,7 | 55,2 | 69,3 | 75,4 | 67,0 | 65,1 | 69,5 | 68,8 | 67,9 | 67,6 | 72,1 |
| Villena | -0,8753684 | 38,595491 | 66,1 | 64,1 | 64,2 | 52,6 | 66,1 | 70,7 | 62,4 | 61,9 | 65,6 | 63,3 | 65,9 | 63,9 | 79,1 |
| Agost | -0,6498214 | 38,421512 | 54,2 | 52,8 | 54,5 | 37,8 | 55,4 | 62,6 | 52,6 | 50,0 | 56,0 | 53,8 | 57,0 | 53,3 | 65,2 |
| Almansa | -1,1075837 | 38,903228 | 79,1 | 77,4 | 77,2 | 73,8 | 78,4 | 79,7 | 77,1 | 76,5 | 77,3 | 76,8 | 75,8 | 77,2 | 80,2 |
| Ontur | -1,4957688 | 38,622866 | 70,5 | 69,2 | 68,6 | 58,2 | 69,0 | 72,5 | 66,6 | 66,1 | 69,2 | 67,3 | 70,0 | 67,9 | 82,1 |
| Caudete | -0,9798818 | 38,734665 | 73,5 | 71,2 | 72,4 | 62,4 | 72,4 | 75,7 | 69,7 | 69,5 | 71,7 | 70,9 | 71,6 | 71,0 | 75,4 |
| La Mojonera | -2,7043824 | 36,787318 | 43,7 | 43,5 | 41,9 | 24,9 | 44,6 | 49,6 | 41,3 | 42,8 | 45,0 | 41,7 | 45,4 | 42,2 | 44,0 |
| Almeria | -2,4024534 | 36,835404 | 26,5 | 25,0 | 25,6 | 10,9 | 27,4 | 33,9 | 26,0 | 23,7 | 28,6 | 27,0 | 29,8 | 25,9 | 38,6 |
| Nijar | -2,1580794 | 36,95057 | 37,6 | 36,5 | 36,4 | 19,4 | 38,7 | 45,3 | 36,3 | 36,5 | 38,7 | 37,2 | 41,1 | 36,7 | 62,7 |
| Tabernas | -2,3023755 | 37,091315 | 58,2 | 58,5 | 56,4 | 44,0 | 57,1 | 62,3 | 53,2 | 56,0 | 56,6 | 54,4 | 58,6 | 55,9 | 71,1 |
| Fiñana | -2,8388277 | 37,156718 | 74,7 | 73,9 | 74,2 | 70,4 | 74,5 | 76,1 | 72,4 | 73,4 | 73,6 | 72,1 | 72,8 | 73,5 | 86,1 |
| Cuevas de Almanzora | -1,7704017 | 37,389125 | 44,6 | 44,8 | 42,1 | 25,6 | 45,0 | 51,2 | 41,4 | 44,3 | 45,1 | 42,8 | 46,8 | 43,1 | 64,7 |
| Huercal-overa | -1,8842832 | 37,412428 | 52,7 | 52,9 | 50,1 | 35,5 | 51,1 | 57,7 | 49,4 | 51,5 | 52,2 | 49,7 | 53,4 | 50,6 | 64,4 |
| Cuevas de Almanzora | -1,800522 | 37,256757 | 32,6 | 31,8 | 30,6 | 14,7 | 33,0 | 39,9 | 32,0 | 31,6 | 33,5 | 32,1 | 36,3 | 31,7 | 50,3 |
| Adra | -2,9923491 | 36,746758 | 33,8 | 29,1 | 29,3 | 20,9 | 33,9 | 43,0 | 32,0 | 29,2 | 33,5 | 32,3 | 36,5 | 32,1 | 41,2 |
| Tijola | -2,457021 | 37,371918 | 66,7 | 67,5 | 64,6 | 54,2 | 65,3 | 69,7 | 61,5 | 65,2 | 65,3 | 62,5 | 66,2 | 64,4 | 81,0 |

| | | | | | | | | | | | | | | | |
|---|---|---|---|---|---|---|---|---|---|---|---|---|---|---|---|
| Totana | -1,5130934 | 37,732459 | 49,3 | 46,9 | 49,9 | 31,0 | 51,3 | 58,4 | 48,2 | 45,4 | 50,6 | 47,9 | 52,5 | 48,3 | 70,8 |
| Alhama | -1,4167602 | 37,7922 | 49,9 | 46,8 | 50,0 | 30,4 | 50,7 | 57,3 | 47,6 | 45,5 | 51,3 | 48,3 | 52,5 | 48,2 | 65,8 |
| Librilla | -1,3382889 | 37,899373 | 53,1 | 51,5 | 52,9 | 35,8 | 56,0 | 61,3 | 51,7 | 50,2 | 54,0 | 51,7 | 55,6 | 52,2 | 64,9 |
| Mazarron | -1,4009916 | 37,56215 | 40,9 | 42,2 | 42,8 | 21,9 | 44,1 | 50,6 | 41,4 | 41,0 | 44,2 | 42,6 | 44,6 | 41,5 | 49,4 |
| Mazarron | -1,3788416 | 37,614572 | 44,1 | 44,1 | 45,0 | 25,4 | 46,1 | 51,8 | 43,3 | 42,5 | 46,4 | 45,0 | 47,6 | 43,7 | 58,6 |
| Zalamea de la Serena | -5,6910276 | 38,678704 | 69,5 | 57,3 | 65,5 | 57,3 | 65,8 | 70,6 | 63,6 | 60,7 | 69,2 | 64,5 | 70,9 | 65,0 | 78,2 |
| Monterrubio de la Serena | -5,3836134 | 38,591582 | 72,8 | 61,9 | 68,1 | 59,4 | 68,4 | 72,1 | 65,6 | 64,5 | 71,1 | 66,9 | 72,1 | 67,5 | 83,3 |
| Don Benito | -5,9062469 | 38,930491 | 63,7 | 50,2 | 58,7 | 51,2 | 62,3 | 65,8 | 58,8 | 54,8 | 64,7 | 59,9 | 67,0 | 59,7 | 75,4 |
| Villagonzalo | -6,1858738 | 38,837247 | 66,9 | 49,5 | 59,6 | 56,6 | 62,7 | 67,4 | 62,4 | 55,5 | 65,1 | 59,3 | 71,2 | 61,5 | 73,8 |
| Jerez de los Caballeros | -6,7369024 | 38,281336 | 64,8 | 49,9 | 59,5 | 47,3 | 60,6 | 67,4 | 60,3 | 54,6 | 62,4 | 57,7 | 66,8 | 59,2 | 75,3 |
| Olivenza | -7,0578251 | 38,720921 | 65,7 | 49,9 | 59,8 | 48,2 | 59,7 | 68,0 | 60,0 | 54,4 | 61,5 | 57,4 | 68,0 | 59,3 | 73,5 |
| Villafranca de los Barros | -6,3485695 | 38,575591 | 68,6 | 52,6 | 62,8 | 52,6 | 62,5 | 69,7 | 63,3 | 57,8 | 65,8 | 61,4 | 69,3 | 62,4 | 74,7 |
| Merida | -6,3192869 | 38,845149 | 67,0 | 50,9 | 60,1 | 56,2 | 63,0 | 67,5 | 62,9 | 55,4 | 65,1 | 59,9 | 70,5 | 61,7 | 75,7 |
| Azuaga | -5,7077922 | 38,391445 | 74,0 | 63,5 | 69,7 | 61,3 | 69,5 | 72,9 | 67,2 | 65,8 | 72,6 | 68,8 | 73,2 | 69,0 | 81,2 |
| Puebla de alcocer | -5,0955855 | 39,074643 | 72,6 | 61,7 | 68,4 | 60,8 | 70,6 | 72,8 | 67,8 | 65,4 | 71,5 | 68,0 | 73,5 | 68,5 | 78,9 |
| Don Benito | -5,858992 | 38,984723 | 62,7 | 48,3 | 58,8 | 51,1 | 61,1 | 65,2 | 58,3 | 53,6 | 65,4 | 60,3 | 66,7 | 59,2 | 74,0 |
| Badajoz | -6,827838 | 38,877039 | 65,6 | 48,6 | 59,5 | 54,9 | 61,9 | 66,3 | 61,5 | 53,5 | 64,3 | 58,6 | 69,5 | 60,4 | 73,4 |
| Pueblonuevo del Guadiana | -6,7328012 | 38,912998 | 65,5 | 49,4 | 58,2 | 54,8 | 62,0 | 65,7 | 61,2 | 53,1 | 63,4 | 58,0 | 68,9 | 60,0 | 73,3 |
| Lobon | -6,6655535 | 38,860185 | 65,8 | 49,2 | 58,1 | 54,5 | 62,1 | 65,8 | 61,6 | 53,9 | 63,7 | 57,8 | 70,1 | 60,2 | 72,7 |
| Arroyo de San Servan | -6,4728164 | 38,858246 | 67,4 | 51,2 | 60,6 | 57,2 | 63,8 | 68,4 | 63,4 | 55,6 | 65,8 | 60,4 | 70,5 | 62,2 | 74,4 |
| Villar de Reina | -5,742601 | 39,102295 | 68,0 | 56,2 | 63,3 | 56,1 | 66,5 | 69,1 | 62,6 | 58,8 | 68,7 | 63,9 | 70,3 | 63,9 | 78,9 |
| Cartagena | -0,9508754 | 37,688833 | 40,1 | 40,5 | 40,7 | 20,9 | 42,3 | 48,9 | 40,6 | 39,3 | 42,2 | 41,1 | 43,6 | 40,0 | 58,8 |
| Murcia | -1,1227711 | 37,831265 | 49,0 | 47,8 | 49,1 | 30,6 | 52,8 | 58,2 | 48,6 | 46,9 | 50,9 | 49,2 | 52,0 | 48,6 | 62,8 |
| Fuente alamo | -1,1292626 | 37,748269 | 39,4 | 35,9 | 39,8 | 21,7 | 42,2 | 49,1 | 38,7 | 34,9 | 41,8 | 39,6 | 44,0 | 38,8 | 63,7 |
| Cartagena | -1,070786 | 37,676671 | 39,1 | 36,4 | 40,0 | 20,7 | 41,9 | 49,0 | 39,0 | 34,9 | 40,6 | 38,5 | 44,2 | 38,6 | 60,0 |
| Cartagena | -0,8037931 | 37,611152 | 40,0 | 40,3 | 40,6 | 22,3 | 43,0 | 50,2 | 41,1 | 39,4 | 44,3 | 42,7 | 44,3 | 40,7 | 58,7 |
| Fuente alamo | -1,2380371 | 37,699008 | 47,7 | 44,0 | 47,9 | 28,4 | 50,2 | 56,1 | 45,9 | 43,2 | 48,9 | 46,3 | 50,8 | 46,3 | 63,2 |
| Casatejada | -5,6781 | 39,867824 | 69,7 | 56,2 | 65,1 | 59,2 | 68,4 | 70,4 | 64,5 | 60,9 | 70,1 | 65,6 | 72,9 | 65,7 | 81,3 |
| Aldehuela del Jerte | -6,2302346 | 40,008316 | 70,4 | 55,5 | 65,0 | 61,6 | 68,4 | 69,2 | 64,4 | 61,7 | 68,3 | 62,8 | 74,3 | 65,6 | 79,5 |
| Moraleja | -6,6759606 | 40,046357 | 69,4 | 55,0 | 64,7 | 58,5 | 66,6 | 69,2 | 65,0 | 56,8 | 67,8 | 62,7 | 71,4 | 64,3 | 80,0 |
| Coria | -6,5458096 | 39,978094 | 70,2 | 55,3 | 64,5 | 61,0 | 67,8 | 69,9 | 65,3 | 59,9 | 68,5 | 62,9 | 73,9 | 65,4 | 78,4 |
| Madrigalejo | -5,5954391 | 39,135847 | 68,9 | 55,6 | 63,1 | 57,0 | 67,4 | 69,8 | 63,6 | 59,3 | 68,9 | 64,3 | 70,9 | 64,4 | 80,7 |
| Valdesalor | -6,4785825 | 39,377191 | 70,9 | 56,6 | 64,7 | 58,4 | 67,1 | 71,0 | 66,3 | 59,1 | 68,6 | 63,9 | 71,8 | 65,3 | 80,3 |

| | | | | | | | | | | | | | | | |
|---|---|---|---|---|---|---|---|---|---|---|---|---|---|---|---|
| Peraleda de la Mata | -5,4639595 | 39,861132 | 72,0 | 59,1 | 68,3 | 61,7 | 69,9 | 73,2 | 67,9 | 64,6 | 71,5 | 67,5 | 73,2 | 68,1 | 82,4 |
| Tejeda de tietar | -5,8600359 | 39,960042 | 73,2 | 65,0 | 69,5 | 64,6 | 74,7 | 73,5 | 70,5 | 70,9 | 73,0 | 68,7 | 76,5 | 70,9 | 77,6 |
| Casar de Palomero | -6,3056933 | 40,298554 | 80,7 | 72,0 | 77,2 | 73,1 | 78,2 | 80,1 | 76,6 | 72,5 | 77,6 | 74,3 | 79,6 | 76,5 | 84,5 |
| Madroñera | -5,7623097 | 39,464885 | 79,1 | 70,2 | 75,7 | 69,3 | 77,4 | 79,2 | 74,9 | 72,5 | 78,1 | 74,4 | 78,0 | 75,4 | 84,2 |
| Guadalupe | -5,3482094 | 39,387141 | 77,9 | 69,1 | 74,8 | 68,5 | 75,8 | 78,1 | 73,8 | 71,5 | 76,8 | 73,3 | 78,0 | 74,3 | 85,3 |
| Alcantara | -6,8981123 | 39,746563 | 67,5 | 51,5 | 61,0 | 58,1 | 64,4 | 68,1 | 64,6 | 55,9 | 65,9 | 60,7 | 71,0 | 62,6 | 78,5 |
| Jarandilla de la Vega | -5,6463392 | 40,101413 | 81,5 | 75,5 | 79,2 | 74,9 | 81,8 | 79,8 | 78,6 | 78,6 | 79,4 | 76,1 | 80,4 | 78,7 | 84,0 |
| Gargantilla | -5,9414268 | 40,239041 | 82,2 | 79,7 | 80,6 | 76,7 | 81,6 | 81,1 | 79,6 | 79,9 | 80,1 | 77,7 | 80,7 | 80,0 | 84,8 |
| Talayuela | -5,5642306 | 40,011198 | 73,8 | 64,5 | 70,6 | 64,5 | 74,7 | 74,0 | 69,5 | 70,3 | 72,7 | 68,7 | 75,8 | 70,8 | 81,1 |
| Valdastillas | -5,8687982 | 40,141215 | 84,1 | 81,4 | 82,9 | 78,6 | 82,5 | 82,3 | 80,9 | 79,8 | 81,4 | 79,2 | 80,6 | 81,2 | 83,5 |
| Cieza | -1,3097249 | 38,235442 | 65,4 | 63,8 | 63,7 | 50,8 | 65,5 | 70,7 | 62,1 | 61,7 | 64,5 | 62,3 | 66,3 | 63,3 | 64,4 |
| Ulea | -1,2578423 | 38,191392 | 56,3 | 55,4 | 55,2 | 38,5 | 59,1 | 63,6 | 54,2 | 52,7 | 57,5 | 54,9 | 58,5 | 55,1 | 71,6 |
| Cieza | -1,4963438 | 38,283888 | 58,5 | 55,7 | 56,9 | 41,9 | 59,1 | 64,3 | 54,6 | 54,0 | 57,8 | 55,6 | 59,7 | 56,2 | 61,1 |
| Calasparra | -1,6947638 | 38,253487 | 59,8 | 56,5 | 58,5 | 42,5 | 59,6 | 65,0 | 55,5 | 53,7 | 59,0 | 56,2 | 60,7 | 57,0 | 72,8 |
| Calasparra | -1,5850018 | 38,269499 | 56,3 | 53,4 | 54,2 | 39,5 | 57,2 | 62,3 | 53,4 | 51,6 | 56,1 | 54,0 | 58,3 | 54,2 | 73,3 |
| Caravaca | -1,980057 | 38,043911 | 73,8 | 72,7 | 73,7 | 61,6 | 73,8 | 75,6 | 70,8 | 71,1 | 73,4 | 71,8 | 71,0 | 71,8 | 88,1 |
| Cehegin | -1,6828994 | 38,110901 | 67,2 | 66,0 | 65,8 | 53,3 | 66,9 | 71,2 | 64,1 | 62,8 | 66,2 | 63,5 | 67,0 | 64,9 | 73,0 |
| Moratalla | -1,813186 | 38,196653 | 70,0 | 67,2 | 70,0 | 52,1 | 70,4 | 72,9 | 66,6 | 66,5 | 69,4 | 68,5 | 66,8 | 67,3 | 79,0 |
| Cehegin | -1,7798922 | 38,104477 | 71,5 | 69,3 | 71,0 | 55,1 | 71,2 | 73,9 | 68,3 | 67,9 | 70,8 | 69,5 | 67,9 | 68,8 | 76,3 |
| Moratalla | -2,0961142 | 38,1145 | 79,5 | 79,7 | 78,4 | 75,2 | 79,3 | 79,3 | 77,8 | 79,0 | 78,1 | 77,2 | 76,3 | 78,2 | 87,1 |
| Vall de Uxo | -0,2304536 | 39,795861 | 51,6 | 51,0 | 51,4 | 35,5 | 55,9 | 62,9 | 51,8 | 47,8 | 54,8 | 54,1 | 56,3 | 52,1 | 64,2 |
| Onda | -0,2444114 | 39,954016 | 57,9 | 57,9 | 55,8 | 43,9 | 60,7 | 66,6 | 56,3 | 51,9 | 60,3 | 58,1 | 59,1 | 57,1 | 69,2 |
| San Rafael del Rio | 0,3675272 | 40,594077 | 58,9 | 58,5 | 56,7 | 45,6 | 61,2 | 68,3 | 56,0 | 51,8 | 60,3 | 58,1 | 60,4 | 57,8 | 75,5 |
| Benicarlo | 0,4014538 | 40,411511 | 51,1 | 50,6 | 49,2 | 37,4 | 55,5 | 62,7 | 49,9 | 45,7 | 54,0 | 51,7 | 55,2 | 51,2 | 67,6 |
| Castellon | -0,1191495 | 39,989342 | 56,0 | 55,2 | 53,6 | 42,8 | 59,5 | 66,2 | 54,2 | 50,4 | 57,5 | 55,7 | 57,8 | 55,4 | 70,3 |
| Burriana | -0,1057138 | 39,887849 | 46,8 | 46,2 | 47,7 | 29,4 | 52,1 | 58,9 | 48,0 | 44,0 | 50,6 | 49,6 | 52,1 | 47,8 | 66,9 |
| Ribera de Cabanes | 0,1464314 | 40,133934 | 57,5 | 56,1 | 55,2 | 43,2 | 60,3 | 67,8 | 54,6 | 51,5 | 58,6 | 56,8 | 59,2 | 56,4 | 67,4 |
| Nules | -0,1683946 | 39,877237 | 48,9 | 49,7 | 50,3 | 33,2 | 54,9 | 61,3 | 50,5 | 46,6 | 54,0 | 52,6 | 54,3 | 50,6 | 65,4 |
| Segorbe | -0,4830876 | 39,817295 | 66,5 | 66,8 | 67,3 | 52,4 | 68,5 | 73,2 | 65,8 | 63,1 | 67,5 | 67,0 | 66,3 | 65,9 | 74,4 |
| Baza | -2,7677154 | 37,564477 | 67,0 | 68,9 | 64,6 | 53,9 | 64,6 | 68,8 | 61,0 | 64,5 | 65,1 | 61,6 | 65,8 | 64,2 | 83,9 |
| Puebla de Don Fadrique | -2,3817176 | 37,876115 | 77,9 | 77,6 | 76,7 | 70,2 | 77,3 | 77,9 | 75,3 | 75,9 | 76,6 | 75,4 | 74,2 | 75,9 | 82,3 |
| Loja | -4,138128 | 37,1693 | 65,9 | 59,3 | 64,8 | 58,7 | 63,7 | 69,6 | 63,1 | 60,3 | 66,3 | 63,8 | 67,0 | 63,9 | 79,5 |
| Iznalloz | -3,5514591 | 37,416406 | 69,9 | 65,8 | 66,4 | 61,6 | 66,7 | 71,3 | 64,0 | 64,4 | 67,1 | 64,3 | 68,0 | 66,3 | 86,0 |

| | | | | | | | | | | | | | | |
|---|---|---|---|---|---|---|---|---|---|---|---|---|---|---|
| Jerez del Marquesado | -3,1498644 | 37,190536 | 73,6 | 72,3 | 72,8 | 68,3 | 72,4 | 74,4 | 70,4 | 71,8 | 72,7 | 70,4 | 71,7 | 71,9 | 84,3 |
| Cadiar | -3,183988 | 36,923123 | 69,7 | 66,5 | 67,7 | 62,3 | 68,1 | 71,8 | 65,3 | 66,4 | 68,0 | 65,2 | 68,3 | 67,2 | 81,4 |
| Zafarraya | -4,1538389 | 36,990314 | 78,9 | 76,8 | 78,5 | 74,7 | 77,9 | 79,7 | 76,4 | 76,9 | 78,7 | 76,7 | 78,0 | 77,6 | 89,9 |
| Padul | -3,600317 | 37,018743 | 69,5 | 65,3 | 66,0 | 61,0 | 66,8 | 71,8 | 64,1 | 65,4 | 66,7 | 64,3 | 68,4 | 66,3 | 80,0 |
| Granada | -3,6385645 | 37,172054 | 67,5 | 62,6 | 63,7 | 57,1 | 64,3 | 70,2 | 61,9 | 62,0 | 64,6 | 61,3 | 65,6 | 63,7 | 77,9 |
| Almuñecar | -3,6790578 | 36,751942 | 51,0 | 45,4 | 47,0 | 38,5 | 49,4 | 57,6 | 47,3 | 46,5 | 49,6 | 48,2 | 51,9 | 48,4 | 54,1 |
| Gibraleon | -7,0278022 | 37,318328 | 40,1 | 30,6 | 38,5 | 23,2 | 38,0 | 49,6 | 41,1 | 34,4 | 39,2 | 34,8 | 45,4 | 37,7 | 59,5 |
| Lepe | -7,2430825 | 37,302685 | 41,3 | 32,7 | 39,3 | 25,1 | 41,1 | 52,1 | 41,1 | 35,7 | 40,4 | 36,5 | 45,3 | 39,2 | 57,0 |
| Gibraleon | -7,059841 | 37,412354 | 46,2 | 36,6 | 43,6 | 30,2 | 45,4 | 55,5 | 45,9 | 40,8 | 44,9 | 40,4 | 50,4 | 43,6 | 66,0 |
| Moguer | -6,7925285 | 37,14648 | 42,8 | 33,4 | 39,7 | 26,0 | 41,2 | 52,2 | 41,9 | 37,4 | 42,1 | 38,2 | 46,7 | 40,1 | 62,3 |
| Niebla | -6,7353478 | 37,347125 | 43,8 | 33,1 | 41,8 | 27,2 | 42,2 | 52,4 | 43,0 | 37,7 | 43,6 | 38,7 | 49,2 | 41,2 | 62,5 |
| Aroche | -6,9449915 | 37,958077 | 66,8 | 53,6 | 60,4 | 49,3 | 62,0 | 68,6 | 61,7 | 55,9 | 64,3 | 59,8 | 67,9 | 60,9 | 71,4 |
| La puebla de Guzman | -7,2483655 | 37,552176 | 54,3 | 45,1 | 52,7 | 37,5 | 52,5 | 62,7 | 53,0 | 48,5 | 53,0 | 48,9 | 57,2 | 51,4 | 69,5 |
| El Campillo | -6,5992719 | 37,660989 | 55,0 | 46,3 | 53,5 | 39,8 | 53,6 | 61,8 | 54,9 | 49,7 | 54,7 | 50,6 | 58,4 | 52,6 | 74,5 |
| La Palma del Condado | -6,5415566 | 37,366968 | 50,4 | 39,6 | 48,5 | 33,6 | 49,1 | 59,4 | 49,9 | 44,9 | 49,6 | 45,5 | 54,5 | 47,7 | 66,3 |
| Almonte | -6,4765444 | 37,148345 | 41,1 | 30,5 | 39,0 | 24,7 | 39,6 | 50,8 | 41,1 | 35,3 | 40,3 | 35,7 | 45,8 | 38,5 | 60,1 |
| Valfarta | -0,1478858 | 41,531503 | 73,7 | 70,7 | 70,3 | 72,0 | 70,8 | 78,0 | 71,1 | 68,5 | 72,2 | 70,9 | 71,8 | 71,8 | 83,5 |
| Zaidin | 0,2890014 | 41,637169 | 72,0 | 68,4 | 69,3 | 71,0 | 69,5 | 76,6 | 69,1 | 67,3 | 70,9 | 69,3 | 70,6 | 70,4 | 79,4 |
| Alcolea de Cinca | 0,0731411 | 41,74095 | 73,1 | 69,5 | 69,7 | 71,8 | 70,1 | 76,5 | 70,1 | 68,2 | 71,7 | 70,8 | 71,3 | 71,2 | 82,2 |
| Tanarite de Litera | 0,3771357 | 41,780947 | 73,7 | 70,7 | 71,3 | 72,3 | 71,0 | 76,6 | 70,2 | 68,8 | 72,0 | 70,7 | 70,5 | 71,6 | 81,9 |
| Lanaja | -0,337846 | 41,786429 | 76,5 | 72,1 | 71,2 | 73,5 | 72,9 | 79,5 | 73,4 | 69,1 | 74,5 | 73,2 | 73,6 | 73,6 | 83,5 |
| Monzon | 0,1273494 | 41,957687 | 76,0 | 73,3 | 73,5 | 73,6 | 73,2 | 77,7 | 73,9 | 71,4 | 74,4 | 72,8 | 72,7 | 73,8 | 83,1 |
| Barbastro | 0,1126102 | 42,013471 | 77,6 | 74,4 | 73,4 | 73,5 | 73,7 | 78,7 | 74,5 | 72,3 | 74,1 | 73,2 | 73,8 | 74,5 | 84,0 |
| Sariñena | -0,1766614 | 41,771411 | 74,5 | 71,8 | 70,8 | 72,6 | 71,0 | 77,6 | 71,3 | 68,8 | 72,3 | 70,8 | 72,0 | 72,1 | 83,1 |
| Huesca | -0,3777068 | 42,105429 | 77,4 | 75,4 | 74,7 | 75,1 | 74,9 | 79,3 | 75,0 | 73,1 | 75,8 | 74,2 | 74,4 | 75,4 | 83,6 |
| Candasnos | 0,094436 | 41,45994 | 70,5 | 67,6 | 68,7 | 69,1 | 68,2 | 75,6 | 68,0 | 65,5 | 70,2 | 68,0 | 69,6 | 69,2 | 83,7 |
| Grañen | -0,3560041 | 41,942469 | 77,2 | 74,7 | 74,0 | 74,5 | 74,1 | 78,9 | 74,5 | 71,8 | 75,1 | 73,6 | 73,5 | 74,7 | 82,9 |
| Huerto | -0,1365362 | 41,966019 | 78,2 | 75,6 | 73,7 | 75,0 | 75,0 | 79,8 | 75,6 | 72,2 | 75,9 | 74,2 | 75,0 | 75,5 | 83,6 |
| Gurrea de Gallego | -0,7311994 | 41,992829 | 79,3 | 76,7 | 76,0 | 75,0 | 75,8 | 78,4 | 75,8 | 75,2 | 77,2 | 76,0 | 74,7 | 76,4 | 85,9 |
| Alfantega | 0,1477817 | 41,821958 | 76,5 | 73,7 | 72,9 | 73,3 | 73,3 | 77,6 | 73,2 | 70,7 | 74,1 | 73,0 | 73,3 | 73,8 | 82,1 |
| Fraga | 0,3539314 | 41,495165 | 68,9 | 66,1 | 65,4 | 68,9 | 68,1 | 74,9 | 66,4 | 64,1 | 68,7 | 66,9 | 68,5 | 67,9 | 76,9 |
| Tardienta | -0,5075831 | 41,969367 | 76,9 | 73,6 | 72,1 | 74,7 | 72,5 | 79,7 | 73,1 | 70,0 | 73,9 | 73,2 | 73,3 | 73,9 | 83,5 |
| San Esteban de Litera | 0,3042037 | 41,882938 | 77,1 | 73,9 | 73,4 | 74,6 | 73,9 | 78,2 | 74,4 | 71,9 | 74,6 | 73,4 | 73,6 | 74,4 | 82,0 |

| | | | | | | | | | | | | | | | |
|---|---|---|---|---|---|---|---|---|---|---|---|---|---|---|---|
| Belver de Cinca | 0,2318291 | 41,742536 | 74,7 | 70,4 | 69,7 | 73,1 | 71,8 | 77,3 | 70,9 | 69,4 | 72,7 | 71,7 | 72,1 | 72,2 | 83,8 |
| Alberuela de Tubo | -0,2573084 | 41,883957 | 74,7 | 71,6 | 70,4 | 73,2 | 71,2 | 77,4 | 72,0 | 69,4 | 72,7 | 71,3 | 72,5 | 72,4 | 84,5 |
| Jumilla | -1,4232837 | 38,394834 | 68,1 | 66,8 | 66,0 | 54,2 | 68,1 | 72,2 | 64,4 | 64,0 | 67,0 | 64,3 | 67,4 | 65,7 | 77,7 |
| Yecla | -1,1859032 | 38,658948 | 73,5 | 72,2 | 71,6 | 62,5 | 72,4 | 75,4 | 69,5 | 69,6 | 71,4 | 69,8 | 71,9 | 70,9 | 85,6 |
| Yecla | -1,1125211 | 38,562731 | 69,7 | 68,0 | 68,0 | 57,1 | 69,4 | 72,8 | 66,5 | 65,6 | 67,6 | 66,2 | 68,7 | 67,2 | 83,7 |
| Jumilla | -1,2407841 | 38,392588 | 69,9 | 68,1 | 67,7 | 57,2 | 68,9 | 73,5 | 66,0 | 65,3 | 68,2 | 66,0 | 69,3 | 67,3 | 73,6 |
| Jumilla | -1,3242866 | 38,31972 | 61,7 | 59,2 | 60,4 | 45,2 | 62,2 | 67,0 | 58,0 | 57,1 | 61,3 | 58,9 | 62,5 | 59,4 | 74,7 |
| Aitona | 0,4609093 | 41,486913 | 69,2 | 64,3 | 63,2 | 67,8 | 67,9 | 75,9 | 65,8 | 62,7 | 68,1 | 66,3 | 68,0 | 67,2 | 77,7 |
| Albesa | 0,6705502 | 41,760356 | 71,4 | 70,9 | 69,9 | 72,3 | 68,9 | 75,5 | 71,7 | 67,8 | 71,1 | 69,5 | 72,4 | 71,0 | 82,6 |
| Alcarras | 0,5506119 | 41,56508 | 71,8 | 69,5 | 69,1 | 69,2 | 70,0 | 76,4 | 68,0 | 67,4 | 70,9 | 68,5 | 69,6 | 70,0 | 81,5 |
| Alfarras | 0,5780224 | 41,819488 | 73,1 | 72,1 | 71,6 | 74,3 | 70,3 | 76,2 | 73,4 | 68,9 | 72,6 | 71,4 | 73,3 | 72,5 | 83,0 |
| Algerri | 0,6483717 | 41,801036 | 74,1 | 73,1 | 72,2 | 74,9 | 71,9 | 76,6 | 73,8 | 70,6 | 73,3 | 72,8 | 74,2 | 73,4 | 80,0 |
| Alguaire | 0,5361346 | 41,742812 | 76,0 | 73,4 | 73,4 | 74,1 | 73,3 | 78,1 | 73,5 | 71,6 | 74,4 | 73,0 | 72,6 | 73,9 | 84,8 |
| Castellnou de Seana | 0,9520619 | 41,65659 | 71,6 | 71,3 | 70,1 | 72,4 | 69,3 | 75,5 | 71,6 | 68,2 | 71,3 | 70,2 | 72,7 | 71,3 | 82,6 |
| Cervera | 1,2967772 | 41,662217 | 78,5 | 77,9 | 77,0 | 79,0 | 75,1 | 80,2 | 77,8 | 75,3 | 76,6 | 76,3 | 76,2 | 77,3 | 83,6 |
| Gimenells i el Pla de la Font | 0,3933398 | 41,658132 | 74,1 | 71,2 | 71,6 | 72,0 | 71,5 | 77,0 | 70,9 | 69,0 | 72,4 | 71,2 | 72,1 | 72,1 | 83,2 |
| Golmes | 0,9248038 | 41,63641 | 70,6 | 69,8 | 69,6 | 70,7 | 68,7 | 75,5 | 70,9 | 66,8 | 71,2 | 69,9 | 72,5 | 70,6 | 75,0 |
| Raimat | 0,4490319 | 41,683272 | 75,9 | 72,9 | 71,5 | 73,2 | 72,4 | 78,7 | 72,4 | 70,6 | 73,8 | 72,5 | 72,8 | 73,3 | 83,1 |
| Balaguer- Monasterio Avellanas | 0,7613663 | 41,879114 | 75,4 | 74,9 | 74,0 | 75,7 | 72,7 | 78,4 | 75,5 | 72,0 | 74,4 | 73,4 | 74,4 | 74,6 | 83,3 |
| El Canos | 1,2041447 | 41,689385 | 77,2 | 76,4 | 75,6 | 77,6 | 74,7 | 79,7 | 76,7 | 73,6 | 75,6 | 75,5 | 75,8 | 76,2 | 84,6 |
| El Poal | 0,8777387 | 41,672786 | 70,7 | 69,8 | 69,7 | 70,7 | 68,7 | 75,5 | 70,9 | 66,8 | 71,2 | 69,9 | 72,5 | 70,6 | 81,3 |
| Sant Marti de Riucorb | 1,0885432 | 41,572353 | 74,5 | 73,8 | 74,1 | 75,3 | 71,5 | 77,9 | 74,7 | 70,5 | 74,0 | 73,0 | 73,8 | 73,9 | 85,1 |
| Seros | 0,4279758 | 41,463784 | 69,1 | 66,5 | 65,6 | 68,1 | 67,7 | 74,7 | 65,5 | 63,5 | 68,1 | 66,3 | 68,0 | 67,6 | 78,2 |
| Tarrega | 1,1626814 | 41,666945 | 74,5 | 73,6 | 73,6 | 75,7 | 72,1 | 77,3 | 74,1 | 70,8 | 74,0 | 73,4 | 74,2 | 73,9 | 80,5 |
| Tornabous | 1,0451011 | 41,68834 | 72,5 | 71,7 | 71,0 | 73,5 | 70,2 | 76,3 | 72,9 | 69,3 | 72,9 | 71,3 | 73,4 | 72,3 | 80,2 |
| Vallfogona de Balaguer | 0,8293888 | 41,784868 | 71,7 | 71,6 | 70,0 | 73,3 | 70,2 | 75,9 | 72,6 | 68,7 | 72,2 | 70,6 | 73,0 | 71,8 | 82,5 |
| Vilanova de Segria | 0,628392 | 41,714499 | 69,8 | 69,2 | 68,6 | 72,2 | 67,8 | 73,4 | 70,1 | 66,7 | 69,7 | 68,6 | 71,3 | 69,8 | 81,9 |
| Lorca | -1,6294551 | 37,601733 | 56,1 | 58,4 | 57,0 | 39,0 | 56,9 | 63,0 | 53,5 | 55,4 | 58,0 | 56,7 | 57,3 | 55,6 | 72,8 |
| Lorca | -1,6938893 | 37,50379 | 52,6 | 52,4 | 50,3 | 36,1 | 50,8 | 58,9 | 48,9 | 50,9 | 51,9 | 49,7 | 52,8 | 50,5 | 73,8 |
| Agoncillo | -2,2904337 | 42,468182 | 82,8 | 81,1 | 80,0 | 78,4 | 80,6 | 81,0 | 80,6 | 80,5 | 80,7 | 80,1 | 80,3 | 80,5 | 89,7 |
| Albelda de Iregua | -2,4718558 | 42,380733 | 83,2 | 81,5 | 80,3 | 80,0 | 80,6 | 80,8 | 81,0 | 80,5 | 80,7 | 80,2 | 79,8 | 80,8 | 90,3 |
| Asenjo | -2,1533164 | 42,340952 | 83,6 | 82,0 | 80,9 | 79,9 | 81,3 | 81,6 | 81,2 | 81,5 | 81,8 | 80,7 | 81,1 | 81,4 | 91,1 |
| Logroño | -2,5136369 | 42,43969 | 83,5 | 81,8 | 80,7 | 79,3 | 80,7 | 81,2 | 81,4 | 81,1 | 81,3 | 80,0 | 80,3 | 81,0 | 91,5 |

| | | | | | | | | | | | | | | | |
|---|---|---|---|---|---|---|---|---|---|---|---|---|---|---|---|
| Santa Engracia del Juvera | -2,2629377 | 42,368971 | 83,2 | 81,8 | 80,7 | 79,0 | 80,7 | 81,2 | 80,8 | 80,8 | 81,2 | 80,2 | 80,8 | 81,0 | 86,3 |
| Aldea Nueva de Ebro | -1,9048679 | 42,222598 | 81,6 | 80,2 | 78,7 | 75,0 | 78,6 | 79,4 | 78,0 | 78,5 | 79,1 | 77,8 | 79,4 | 78,8 | 86,4 |
| Alfaro | -1,7776916 | 42,152119 | 82,5 | 80,6 | 79,0 | 72,2 | 79,7 | 79,4 | 79,8 | 79,8 | 79,8 | 78,2 | 78,4 | 79,0 | 88,6 |
| Calahorra | -2,001826 | 42,334834 | 81,5 | 79,9 | 79,7 | 76,4 | 79,1 | 80,4 | 78,4 | 79,1 | 79,1 | 77,7 | 79,6 | 79,2 | 90,9 |
| Corvera (Cabreton) | -1,8924542 | 42,006904 | 82,8 | 80,2 | 79,6 | 75,1 | 79,9 | 80,5 | 79,7 | 79,5 | 80,4 | 78,7 | 79,2 | 79,6 | 86,1 |
| Igea | -1,9937535 | 42,05775 | 82,7 | 81,1 | 80,6 | 78,1 | 80,8 | 80,2 | 80,7 | 80,1 | 80,7 | 80,3 | 79,2 | 80,4 | 88,5 |
| Quel | -2,037178 | 42,252488 | 82,5 | 81,1 | 79,5 | 77,1 | 79,7 | 80,7 | 80,1 | 79,3 | 80,2 | 78,6 | 80,0 | 79,9 | 85,4 |
| Rincon de Soto | -1,8508464 | 42,251583 | 81,7 | 80,2 | 78,7 | 75,0 | 78,6 | 79,4 | 78,0 | 78,5 | 79,1 | 77,8 | 79,4 | 78,8 | 85,8 |
| Aguilas | -1,5921627 | 37,418665 | 38,8 | 37,9 | 39,5 | 20,4 | 41,0 | 48,5 | 38,3 | 36,8 | 40,9 | 39,3 | 43,1 | 38,6 | 48,9 |
| Lorca | -1,8177885 | 37,855634 | 73,2 | 71,8 | 73,5 | 59,3 | 73,6 | 75,6 | 70,2 | 70,9 | 72,7 | 72,1 | 70,6 | 71,2 | 83,5 |
| Lorca | -1,623984 | 37,4878 | 52,3 | 53,9 | 53,3 | 33,9 | 54,1 | 61,7 | 51,4 | 52,0 | 54,7 | 54,3 | 53,5 | 52,3 | 59,9 |
| Puerto Lumbreras | -1,7255508 | 37,590472 | 52,3 | 52,8 | 50,0 | 36,4 | 49,9 | 57,0 | 48,2 | 50,2 | 52,0 | 49,3 | 53,4 | 50,1 | 68,9 |
| Mula | -1,4294482 | 38,065871 | 57,6 | 56,0 | 56,0 | 39,8 | 59,7 | 64,0 | 54,8 | 53,5 | 57,7 | 55,4 | 58,5 | 55,7 | 61,4 |
| Mula | -1,46674 | 38,041031 | 59,6 | 57,4 | 58,4 | 43,1 | 60,6 | 66,8 | 56,9 | 54,7 | 60,3 | 57,3 | 61,8 | 57,9 | 60,6 |
| Torres de Cotillas | -1,3025362 | 38,006971 | 47,7 | 42,9 | 47,2 | 28,2 | 49,0 | 55,1 | 44,9 | 41,9 | 48,7 | 45,7 | 50,5 | 45,6 | 64,2 |
| Molina del Segura | -1,2206884 | 38,127483 | 49,4 | 44,8 | 49,4 | 30,3 | 50,3 | 57,1 | 46,6 | 44,1 | 51,1 | 48,2 | 52,2 | 47,6 | 62,5 |
| Molina del Segura | -1,2336707 | 38,071139 | 47,3 | 43,4 | 47,5 | 26,8 | 48,0 | 55,5 | 45,8 | 42,8 | 48,3 | 45,3 | 50,5 | 45,6 | 64,6 |
| Abanilla | -1,0655079 | 38,170041 | 46,9 | 43,3 | 47,1 | 28,1 | 48,8 | 55,1 | 45,7 | 42,5 | 48,2 | 45,9 | 50,1 | 45,6 | 64,5 |
| Fortuna | -1,1526819 | 38,161028 | 47,4 | 44,1 | 47,3 | 29,4 | 48,4 | 55,8 | 45,7 | 44,0 | 48,1 | 46,1 | 51,0 | 46,1 | 63,7 |
| Ojos | -1,3394287 | 38,113316 | 53,9 | 52,1 | 53,9 | 36,7 | 55,7 | 61,6 | 51,3 | 49,3 | 55,1 | 53,0 | 56,6 | 52,7 | 52,7 |
| Beniel | -0,9997837 | 38,034507 | 44,6 | 41,8 | 45,2 | 25,8 | 47,2 | 53,9 | 43,7 | 41,1 | 46,1 | 43,5 | 48,9 | 43,8 | 63,2 |
| Murcia | -1,2682702 | 37,898166 | 56,6 | 55,2 | 56,3 | 39,4 | 58,9 | 63,7 | 53,6 | 53,1 | 56,8 | 54,8 | 58,3 | 55,1 | 48,9 |
| Murcia | -0,9840042 | 37,977528 | 47,4 | 45,1 | 47,9 | 28,7 | 49,9 | 56,4 | 46,6 | 43,4 | 48,5 | 46,2 | 50,9 | 46,5 | 56,4 |
| Murcia | -1,1347189 | 37,940075 | 51,2 | 49,4 | 51,3 | 33,4 | 53,7 | 59,5 | 49,7 | 48,1 | 52,5 | 49,8 | 54,4 | 50,3 | 61,4 |
| Fitero | -1,8426437 | 42,046077 | 83,0 | 80,9 | 80,0 | 74,9 | 80,3 | 80,3 | 80,6 | 80,1 | 81,1 | 79,5 | 79,7 | 80,0 | 85,4 |
| Cascante | -1,7239555 | 42,034371 | 81,9 | 80,2 | 78,7 | 71,1 | 79,1 | 79,6 | 78,4 | 78,6 | 80,0 | 78,1 | 78,6 | 78,6 | 86,2 |
| Ablitas | -1,6447131 | 41,996446 | 77,9 | 75,2 | 74,2 | 71,9 | 74,1 | 78,2 | 73,2 | 73,4 | 76,3 | 74,0 | 73,9 | 74,8 | 85,9 |
| Murillo el fruto | -1,4871859 | 42,38498 | 80,8 | 80,3 | 79,6 | 76,4 | 78,6 | 80,4 | 78,2 | 78,7 | 79,1 | 78,8 | 78,8 | 79,1 | 84,5 |
| Miranda de Arga | -1,8087315 | 42,511252 | 82,5 | 80,8 | 79,4 | 77,6 | 79,7 | 80,5 | 79,8 | 79,7 | 80,4 | 79,1 | 80,1 | 80,0 | 86,1 |
| Falces | -1,7925482 | 42,409669 | 83,3 | 81,7 | 80,3 | 77,7 | 80,5 | 81,5 | 80,8 | 80,8 | 80,8 | 79,5 | 80,7 | 80,7 | 85,8 |
| Bargota | -2,2992201 | 42,477657 | 82,8 | 81,1 | 80,0 | 78,4 | 80,7 | 81,0 | 80,6 | 80,5 | 80,7 | 80,1 | 80,3 | 80,6 | 85,7 |
| Bardenas Reales | -1,5187546 | 42,295154 | 81,9 | 80,9 | 79,8 | 77,6 | 79,7 | 80,5 | 78,3 | 80,0 | 80,0 | 79,2 | 79,3 | 79,7 | 86,0 |
| Los Arcos | -2,1845206 | 42,539308 | 83,8 | 81,7 | 80,7 | 79,1 | 80,9 | 81,5 | 81,2 | 81,2 | 81,4 | 80,4 | 80,3 | 81,1 | 86,9 |

| | | | | | | | | | | | | | | |
|---|---|---|---|---|---|---|---|---|---|---|---|---|---|---|
| Sesma | -2,126631 | 42,473409 | 83,9 | 82,0 | 80,5 | 79,0 | 80,6 | 81,1 | 81,0 | 81,0 | 81,4 | 80,6 | 81,0 | 81,1 | 90,1 |
| Cadreita | -1,6556731 | 42,26433 | 79,8 | 78,4 | 78,5 | 74,7 | 77,4 | 79,7 | 76,1 | 78,3 | 77,6 | 77,6 | 77,6 | 77,8 | 82,6 |
| Bardenas Reales | -1,7183027 | 42,207768 | 81,6 | 80,4 | 79,4 | 75,2 | 79,5 | 80,5 | 78,7 | 79,5 | 79,7 | 78,1 | 80,1 | 79,3 | 79,3 |
| Sartaguda | -2,0512344 | 42,361948 | 82,3 | 81,1 | 79,8 | 76,8 | 79,8 | 80,7 | 79,2 | 80,1 | 80,1 | 79,0 | 80,2 | 79,9 | 78,2 |
| Olite | -1,662579 | 42,423779 | 81,6 | 79,9 | 79,6 | 77,4 | 78,9 | 80,9 | 78,1 | 79,8 | 79,7 | 79,1 | 78,7 | 79,4 | 85,7 |
| Murillo el Cuende | -1,6153521 | 42,361474 | 81,4 | 80,0 | 79,5 | 76,8 | 78,6 | 80,7 | 78,0 | 79,4 | 79,5 | 78,9 | 78,5 | 79,2 | 82,4 |
| Corella | -1,8398436 | 42,115577 | 82,2 | 81,0 | 78,7 | 72,2 | 79,6 | 79,4 | 79,7 | 79,6 | 80,0 | 78,6 | 78,6 | 79,1 | 81,2 |
| Funes | -1,8066789 | 42,287885 | 81,5 | 80,0 | 79,1 | 75,6 | 78,4 | 79,8 | 78,3 | 78,9 | 79,3 | 77,9 | 79,7 | 79,0 | 79,8 |
| Lerin | -1,9763006 | 42,503595 | 83,1 | 80,9 | 80,3 | 78,5 | 80,7 | 80,7 | 79,9 | 80,4 | 80,7 | 80,0 | 80,6 | 80,5 | 87,3 |
| Los Palacios y Villafranca | -5,9390554 | 37,179127 | 41,5 | 32,7 | 41,1 | 26,7 | 40,0 | 49,1 | 39,8 | 36,1 | 43,1 | 38,2 | 48,8 | 39,7 | 56,8 |
| Las cabezas de San Juan | -5,884722 | 37,01556 | 41,3 | 32,2 | 38,7 | 27,0 | 40,1 | 47,7 | 37,5 | 35,2 | 42,0 | 37,6 | 47,0 | 38,8 | 59,2 |
| Lebrija | -6,1261602 | 36,976641 | 39,5 | 30,4 | 38,3 | 24,7 | 38,4 | 47,6 | 37,4 | 34,5 | 40,2 | 36,6 | 45,2 | 37,5 | 63,1 |
| Aznalcazar | -6,2733503 | 37,151795 | 44,0 | 34,7 | 43,4 | 28,2 | 43,8 | 54,7 | 45,5 | 40,3 | 44,0 | 40,1 | 50,1 | 42,6 | 62,9 |
| Isla Mayor | -6,1512787 | 37,098521 | 41,0 | 32,5 | 39,3 | 26,4 | 39,7 | 49,9 | 38,7 | 35,1 | 41,7 | 38,2 | 46,7 | 39,0 | 58,0 |
| La puebla del Rio | -6,1338321 | 37,226032 | 41,5 | 31,9 | 40,4 | 26,7 | 40,2 | 49,7 | 39,0 | 35,4 | 42,3 | 38,1 | 48,2 | 39,4 | 60,7 |
| La puebla del Rio II | -6,0465691 | 37,080174 | 41,4 | 32,6 | 39,4 | 26,8 | 40,6 | 50,7 | 39,5 | 36,5 | 41,8 | 38,1 | 47,1 | 39,5 | 62,9 |
| Ecija | -5,0770704 | 37,592934 | 50,1 | 41,8 | 45,1 | 40,7 | 47,3 | 56,1 | 46,2 | 42,9 | 49,8 | 47,0 | 53,2 | 47,3 | 66,3 |
| La Luisiana | -5,2281407 | 37,525293 | 51,6 | 40,6 | 50,0 | 37,4 | 49,1 | 57,3 | 47,1 | 45,3 | 51,8 | 47,9 | 56,2 | 48,6 | 68,1 |
| Carmona | -5,587615 | 37,400903 | 44,5 | 35,1 | 43,5 | 29,2 | 44,0 | 52,5 | 42,4 | 37,7 | 44,9 | 41,6 | 50,2 | 42,3 | 63,8 |
| Osuna | -5,1348377 | 37,25503 | 49,0 | 40,5 | 48,0 | 36,0 | 48,2 | 57,1 | 46,4 | 44,2 | 50,8 | 47,2 | 54,5 | 47,4 | 64,9 |
| La Rinconada | -5,924839 | 37,456832 | 41,4 | 31,2 | 39,4 | 28,7 | 39,6 | 48,6 | 38,1 | 35,6 | 42,6 | 38,7 | 48,0 | 39,3 | 64,9 |
| Sanlucar La Mayor | -6,2550749 | 37,42179 | 47,9 | 36,4 | 46,5 | 30,5 | 46,8 | 55,9 | 47,7 | 42,9 | 47,4 | 42,5 | 53,0 | 45,2 | 66,6 |
| Villanueva del Rio y Minas | -5,6840093 | 37,613036 | 43,9 | 33,9 | 42,6 | 29,8 | 42,0 | 51,1 | 41,5 | 37,6 | 44,2 | 40,2 | 50,6 | 41,6 | 59,9 |
| Lora del Rio | -5,5407037 | 37,660906 | 49,1 | 39,6 | 47,9 | 34,2 | 46,7 | 55,4 | 46,2 | 43,2 | 49,3 | 45,6 | 55,0 | 46,6 | 65,9 |
| Los Molares | -5,6729697 | 37,176152 | 43,9 | 35,1 | 42,2 | 29,3 | 42,6 | 51,4 | 40,7 | 37,2 | 44,6 | 40,2 | 49,3 | 41,5 | 63,9 |
| Guillena | -6,06419 | 37,514568 | 44,0 | 34,2 | 41,1 | 29,4 | 41,9 | 50,0 | 40,1 | 37,5 | 43,7 | 39,5 | 49,2 | 41,0 | 64,0 |
| Puebla Cazalla | -5,3509152 | 37,218131 | 50,8 | 41,3 | 48,6 | 34,8 | 48,6 | 57,0 | 47,4 | 44,5 | 52,3 | 47,9 | 55,6 | 48,1 | 62,6 |
| Alcala del Rio | -5,9641033 | 37,512529 | 41,9 | 31,6 | 39,1 | 26,6 | 38,8 | 47,8 | 37,5 | 34,8 | 41,9 | 37,2 | 46,1 | 38,5 | 56,5 |
| San Javier | -0,819705 | 37,791664 | 37,5 | 34,6 | 38,5 | 19,7 | 41,0 | 48,6 | 37,3 | 33,6 | 39,9 | 38,2 | 42,1 | 37,4 | 56,5 |
| Torre Pacheco | -0,8985888 | 37,773803 | 35,8 | 33,4 | 36,1 | 18,9 | 39,2 | 46,3 | 35,8 | 32,6 | 38,5 | 35,7 | 41,2 | 35,8 | 63,7 |
| San Javier | -0,8836862 | 37,848045 | 38,3 | 35,4 | 39,6 | 20,6 | 42,2 | 48,5 | 38,2 | 34,0 | 41,0 | 38,8 | 43,2 | 38,2 | 57,9 |
| Torre Pacheco | -0,9316281 | 37,823827 | 40,5 | 37,3 | 41,0 | 21,5 | 42,7 | 49,6 | 40,1 | 36,1 | 42,7 | 40,0 | 45,0 | 39,7 | 59,6 |
| Torre Pacheco | -0,9867861 | 37,74765 | 37,9 | 33,9 | 38,1 | 19,5 | 40,2 | 47,8 | 36,7 | 32,9 | 39,8 | 37,2 | 42,5 | 37,0 | 63,7 |

| | | | | | | | | | | | | | | | |
|---|---|---|---|---|---|---|---|---|---|---|---|---|---|---|---|
| Pedralba | -0,7175861 | 39,567014 | 59,7 | 55,1 | 55,4 | 42,7 | 60,6 | 66,0 | 56,1 | 52,0 | 59,3 | 57,4 | 59,5 | 56,7 | 69,6 |
| Liria | -0,627062 | 39,691055 | 59,8 | 53,4 | 54,5 | 44,8 | 61,7 | 64,5 | 55,4 | 52,4 | 58,8 | 57,4 | 59,0 | 56,5 | 73,5 |
| Benifayo | -0,4618662 | 39,280627 | 43,7 | 44,4 | 44,6 | 28,8 | 49,2 | 57,6 | 45,7 | 41,4 | 49,1 | 46,3 | 49,6 | 45,5 | 60,1 |
| Cheste | -0,7444395 | 39,518889 | 66,0 | 59,4 | 61,8 | 49,0 | 66,3 | 70,2 | 61,5 | 59,2 | 64,5 | 62,5 | 63,3 | 62,2 | 67,3 |
| Tabernes de Valldigna | -0,2380292 | 39,095261 | 48,3 | 48,3 | 48,2 | 30,3 | 52,3 | 60,2 | 48,1 | 44,4 | 51,6 | 50,1 | 52,3 | 48,6 | 53,4 |
| Villanueva de Castellon | -0,5242892 | 39,065567 | 47,1 | 47,3 | 48,0 | 31,1 | 51,2 | 60,0 | 48,5 | 43,4 | 51,3 | 49,0 | 52,5 | 48,1 | 64,4 |
| Sagunto | -0,2663216 | 39,647534 | 48,3 | 48,4 | 48,9 | 32,2 | 53,1 | 60,4 | 49,4 | 45,9 | 52,3 | 51,6 | 53,6 | 49,5 | 63,2 |
| Benavites | -0,2162186 | 39,730391 | 51,3 | 52,2 | 52,6 | 34,4 | 55,8 | 64,0 | 52,7 | 48,9 | 55,5 | 54,2 | 56,1 | 52,5 | 61,1 |
| Moncada | -0,3989602 | 39,587729 | 49,3 | 49,9 | 50,4 | 33,4 | 54,3 | 61,4 | 50,2 | 46,6 | 53,8 | 52,6 | 54,5 | 50,6 | 64,9 |
| Carcagente | -0,4461657 | 39,113604 | 45,0 | 46,4 | 46,4 | 30,0 | 50,1 | 58,1 | 46,9 | 43,3 | 50,0 | 48,1 | 50,3 | 46,8 | 65,5 |
| Carlet | -0,5459462 | 39,22642 | 56,0 | 48,4 | 50,7 | 36,2 | 57,0 | 62,5 | 51,1 | 48,3 | 55,1 | 53,0 | 56,1 | 52,2 | 78,8 |
| Luchente | -0,3600825 | 38,938508 | 62,2 | 62,6 | 63,4 | 47,4 | 65,5 | 72,2 | 62,0 | 59,4 | 64,4 | 63,6 | 64,1 | 62,4 | 89,7 |
| Requena | -1,2323883 | 39,504667 | 78,6 | 75,9 | 75,6 | 72,2 | 77,5 | 78,4 | 75,7 | 74,1 | 76,5 | 75,8 | 74,9 | 75,9 | 62,1 |
| Algemesi | -0,4353656 | 39,216442 | 43,7 | 44,6 | 45,5 | 27,6 | 49,3 | 56,6 | 46,0 | 41,0 | 48,8 | 46,7 | 49,7 | 45,4 | 66,7 |
| Campo Arcis | -1,1622154 | 39,433357 | 77,5 | 74,0 | 74,3 | 70,3 | 75,9 | 77,6 | 74,1 | 71,9 | 75,1 | 73,9 | 73,5 | 74,4 | 64,2 |
| Betera | -0,4685258 | 39,597708 | 52,3 | 52,1 | 52,6 | 36,1 | 55,7 | 62,7 | 52,6 | 48,5 | 54,4 | 54,3 | 56,9 | 52,6 | 68,2 |
| Picasent | -0,4976324 | 39,362484 | 50,0 | 50,7 | 50,7 | 33,4 | 54,2 | 62,3 | 50,9 | 46,5 | 54,3 | 52,6 | 54,7 | 50,9 | 66,6 |
| Montesa | -0,6383798 | 38,954502 | 64,1 | 59,3 | 61,2 | 47,8 | 64,6 | 69,5 | 60,3 | 57,4 | 63,8 | 62,3 | 62,2 | 61,1 | 57,5 |
| Jativa | -0,5497109 | 38,998803 | 50,3 | 51,4 | 51,9 | 35,1 | 55,3 | 61,9 | 50,9 | 47,0 | 54,9 | 53,9 | 55,3 | 51,6 | 83,4 |
| Villalonga | -0,2042579 | 38,892111 | 49,3 | 49,6 | 51,1 | 34,4 | 55,3 | 61,9 | 50,8 | 46,4 | 54,4 | 53,4 | 54,6 | 51,0 | 61,9 |
| Gandia | -0,2506841 | 38,964297 | 54,4 | 55,1 | 55,2 | 39,4 | 58,4 | 66,0 | 54,6 | 50,9 | 57,7 | 57,2 | 58,5 | 55,2 | 60,7 |
| Bolbaite | -0,6901658 | 39,069153 | 65,1 | 59,6 | 61,6 | 49,3 | 66,3 | 70,8 | 61,3 | 57,6 | 64,5 | 62,5 | 62,8 | 61,9 | 70,2 |
| Chulilla | -0,8322414 | 39,67678 | 66,1 | 59,5 | 61,0 | 50,6 | 66,5 | 70,2 | 61,5 | 59,0 | 64,7 | 63,2 | 64,0 | 62,4 | 73,1 |
| Almoacid de la Sierra | -1,3299642 | 41,452078 | 77,7 | 75,1 | 74,4 | 71,3 | 73,4 | 77,3 | 73,0 | 72,2 | 75,6 | 73,2 | 73,1 | 74,2 | 83,0 |
| Belchite | -0,7216155 | 41,350306 | 75,9 | 73,8 | 72,4 | 69,4 | 72,1 | 76,1 | 71,0 | 71,7 | 74,8 | 72,4 | 71,7 | 72,9 | 85,1 |
| Quinto | -0,5186373 | 41,388348 | 69,1 | 63,3 | 63,9 | 69,5 | 66,7 | 74,5 | 64,7 | 61,6 | 68,8 | 66,4 | 68,5 | 67,0 | 81,7 |
| Fabara | 0,1540344 | 41,167877 | 69,2 | 65,1 | 64,9 | 67,5 | 67,3 | 75,1 | 65,0 | 62,5 | 68,8 | 67,0 | 68,3 | 67,3 | 81,1 |
| Epila | -1,2820466 | 41,583234 | 75,8 | 72,2 | 72,1 | 68,0 | 71,4 | 76,8 | 71,1 | 69,8 | 73,3 | 70,3 | 71,6 | 72,0 | 83,5 |
| Ejea de los Caballeros | -1,1961298 | 42,097715 | 79,5 | 77,0 | 76,3 | 74,9 | 76,2 | 78,7 | 76,1 | 75,2 | 77,9 | 76,4 | 75,8 | 76,7 | 85,8 |
| Sabada | -1,309387 | 42,267312 | 80,9 | 80,1 | 79,0 | 76,1 | 78,5 | 80,1 | 77,7 | 78,9 | 78,8 | 78,3 | 78,6 | 78,8 | 90,3 |
| Luna | -0,9359498 | 42,095761 | 80,9 | 78,2 | 77,8 | 76,8 | 76,9 | 79,6 | 77,8 | 77,5 | 78,9 | 77,7 | 76,3 | 78,0 | 89,1 |
| Santa Engracia | -1,3305062 | 41,921246 | 77,1 | 72,8 | 72,9 | 70,7 | 72,8 | 76,4 | 72,3 | 72,4 | 74,8 | 73,4 | 72,8 | 73,5 | 85,7 |
| Pastriz | -0,7461599 | 41,59514 | 76,5 | 74,1 | 73,0 | 69,9 | 71,9 | 76,3 | 70,4 | 72,4 | 74,6 | 71,8 | 72,3 | 73,0 | 81,1 |

| | | | | | | | | | | | | | | |
|---|---|---|---|---|---|---|---|---|---|---|---|---|---|---|
| Zaragoza | -0,823819 | 41,71363 | 77,7 | 74,9 | 74,1 | 72,3 | 73,3 | 76,9 | 72,7 | 73,0 | 75,7 | 74,1 | 73,4 | 74,4 | 80,4 |
| Calatayud | -1,6583593 | 41,332167 | 79,8 | 78,5 | 78,2 | 75,8 | 77,3 | 78,6 | 77,2 | 77,1 | 78,0 | 76,9 | 75,6 | 77,5 | 87,5 |
| Borja | -1,5076903 | 41,855146 | 78,3 | 76,3 | 74,9 | 72,5 | 75,0 | 78,2 | 74,2 | 74,2 | 76,7 | 74,9 | 73,9 | 75,4 | 86,2 |
| Tarazona | -1,7458841 | 41,916177 | 83,5 | 81,5 | 80,8 | 75,7 | 81,6 | 81,1 | 81,4 | 81,0 | 81,2 | 80,2 | 80,0 | 80,7 | 84,8 |
| Caspe | -0,0710973 | 41,303968 | 65,5 | 62,7 | 63,4 | 66,2 | 65,3 | 73,6 | 62,7 | 60,6 | 65,7 | 63,7 | 65,5 | 65,0 | 80,9 |
| Osera de Ebro | -0,5363755 | 41,545081 | 71,8 | 68,5 | 67,8 | 70,8 | 69,3 | 77,5 | 68,8 | 65,6 | 70,9 | 68,7 | 69,5 | 69,9 | 81,1 |
| Daroca | -1,4247095 | 41,108073 | 80,4 | 78,8 | 78,3 | 77,4 | 78,5 | 78,4 | 78,9 | 77,4 | 78,1 | 77,5 | 76,2 | 78,2 | 86,7 |
| Zuera | -0,7511391 | 41,869389 | 78,2 | 75,0 | 75,0 | 73,4 | 74,4 | 77,3 | 74,4 | 73,7 | 76,2 | 74,9 | 74,2 | 75,1 | 84,8 |
| El Bayo | -1,2487734 | 42,175713 | 80,1 | 77,2 | 76,9 | 75,3 | 76,4 | 79,8 | 77,4 | 76,1 | 77,7 | 76,6 | 75,9 | 77,2 | 86,4 |
| Tauste | -1,1428386 | 42,00023 | 77,2 | 73,5 | 73,0 | 71,3 | 72,8 | 76,6 | 72,3 | 71,5 | 75,1 | 73,1 | 72,7 | 73,5 | 85,9 |
| Boquiñeni | -1,2496818 | 41,843217 | 77,5 | 73,9 | 72,8 | 71,8 | 73,2 | 77,1 | 71,6 | 71,4 | 74,9 | 72,7 | 71,8 | 73,5 | 84,7 |
