# Supplementary material for: Agroclimatic Metrics for the Main Stone Fruit Producing Areas in Spain in Current and Future Climate Change Scenarios: Implications From an Adaptive Point of View
Source: Front Plant Sci. 2022 Jun 8;13:842628. doi: 10.3389/fpls.2022.842628 (PMC9213681; doi:10.3389/fpls.2022.842628)
Supplement: Supplementary file 4 [file Data_Sheet_4.PDF]

Supplementary Table 4. Mean accumulated portions (1st November - end February) for 2045-2065, RCP 8.5 at each location.
The last column shows the chilling accumulation for the current situation, for comparison purposes
M1: bcc-csm1-1-m; **M2:** BNU-ESM; **M3:** CanESM2; **M4:** CMCC-CM; **M5:** GFDL-ESM2G; **M6:** inmcm4
M7: IPSL-CM5A-LR; **M8:** MIROC-ESM; **M9:** MPI-ESM-LR; **M10:** MPI-ESM-MR; **M11:** MRI-CGCM3

Municipality	Longitude	Latitude	M1	M2	M3	M4	M5	M6	M7	M8	M9	M10	M11	MEAN	CURRENT
Campo de Mirra	-0,7729762	38,679366	71,8	72,8	70,7	65,4	72,9	76,6	69,0	69,5	69,3	68,6	71,7	70,8	81,4
Villajoyosa	-0,2561866	38,527917	34,9	33,0	34,7	15,3	42,2	46,9	31,1	31,0	34,1	35,6	39,1	34,3	52,9
Ondara	0,0065631	38,818581	37,7	36,8	36,8	18,9	45,3	52,1	34,6	33,8	37,7	38,4	42,5	37,7	56,1
Denia Gata	0,082579	38,792724	40,8	40,0	41,3	20,7	46,7	53,2	37,2	38,9	39,4	41,8	44,0	40,4	61,2
Pinoso	-1,060721	38,427413	70,1	69,7	68,4	61,1	71,2	74,9	65,8	67,5	66,6	65,0	70,5	68,2	81,6
Monforte del Cid	-0,7303963	38,398862	45,6	43,1	44,0	26,2	48,6	54,4	40,4	41,0	43,0	41,8	47,9	43,3	64,4
Crevillente	-0,7831581	38,240831	36,6	33,6	34,3	17,0	40,1	46,0	31,6	32,5	33,7	32,5	39,1	34,3	53,2
Almoradi	-0,7745396	38,031431	36,5	32,0	35,7	16,0	40,3	45,9	31,7	33,0	33,7	33,2	38,9	34,3	55,6
Callosa de Sarria	-0,1044988	38,650249	50,8	50,1	50,7	33,9	55,5	62,7	47,3	48,7	49,2	50,0	53,2	50,2	49,7
Pilar de la Horadada	-0,8125284	37,868588	32,3	28,3	31,4	12,5	37,2	43,2	27,7	28,2	30,0	29,2	36,3	30,6	55,7
Catral	-0,8055704	38,153124	35,6	31,4	34,2	15,0	39,1	44,1	30,5	31,3	32,6	32,5	38,2	33,1	56,3
Altea	-0,0795078	38,603643	42,1	41,5	42,7	23,5	48,3	54,3	38,0	40,4	41,2	43,2	44,5	41,8	50,2
Planes	-0,3529076	38,78476	63,5	62,4	63,5	50,3	65,0	71,8	59,3	60,5	61,0	61,3	63,8	62,1	72,1
Villena	-0,8753684	38,595491	61,1	60,3	59,4	48,1	62,3	67,3	55,7	57,3	58,0	55,1	62,4	58,8	79,1
Agost	-0,6498214	38,421512	49,4	47,1	48,1	31,2	51,9	58,3	44,6	45,0	46,8	46,1	51,6	47,3	65,2
Almansa	-1,1075837	38,903228	76,0	76,1	74,8	72,4	76,5	78,4	74,1	72,6	74,3	73,7	73,6	74,8	80,2
Ontur	-1,4957688	38,622866	65,8	65,8	63,3	54,2	66,6	70,8	61,0	61,8	62,5	60,7	67,4	63,6	82,1
Caudete	-0,9798818	38,734665	68,9	68,3	67,2	58,8	69,5	73,5	64,5	65,4	65,5	63,8	69,1	66,8	75,4
La Mojonera	-2,7043824	36,787318	38,6	37,1	35,8	19,3	39,8	46,2	31,7	39,3	35,6	35,3	40,5	36,3	44,0
Almeria	-2,4024534	36,835404	23,2	20,0	21,3	5,9	24,9	31,0	17,2	22,6	20,7	20,2	24,6	21,1	38,6
Nijar	-2,1580794	36,95057	33,2	31,4	31,3	12,8	34,7	42,1	27,2	33,1	30,9	30,1	35,6	31,1	62,7
Tabernas	-2,3023755	37,091315	53,2	52,7	50,3	37,0	52,3	59,1	45,4	51,7	48,1	47,1	54,4	50,1	71,1
Fiñana	-2,8388277	37,156718	71,4	71,4	70,6	69,4	70,2	74,5	69,0	70,3	69,9	68,1	71,0	70,5	86,1
Cuevas de Almanzora	-1,7704017	37,389125	39,3	39,3	35,9	19,8	39,9	47,5	32,9	39,9	36,9	35,4	41,5	37,1	64,7
Huercal-overa	-1,8842832	37,412428	47,4	47,3	43,9	28,9	46,4	53,9	41,1	47,3	43,3	42,7	49,4	44,7	64,4
Cuevas de Almanzora	-1,800522	37,256757	28,8	26,3	25,7	9,3	30,2	36,7	23,0	28,9	25,7	25,8	30,9	26,5	50,3
Adra	-2,9923491	36,746758	29,2	23,6	23,8	14,6	29,4	37,2	23,7	26,4	25,1	25,1	30,9	26,3	41,2
Tijola	-2,457021	37,371918	61,8	62,7	59,1	50,1	60,6	65,9	54,4	60,5	56,8	54,6	63,2	59,1	81,0

Totana	-1,5130934	37,732459	44,7	41,1	42,7	24,8	47,0	53,5	39,5	39,3	41,7	39,6	46,9	41,9	70,8
Alhama	-1,4167602	37,7922	44,3	41,4	43,2	23,9	46,8	53,2	39,2	39,9	41,4	39,9	46,9	41,8	65,8
Librilla	-1,3382889	37,899373	48,0	46,0	46,5	29,2	51,5	56,3	43,5	45,0	44,7	44,3	50,1	45,9	64,9
Mazarron	-1,4009916	37,56215	36,3	36,1	36,9	14,6	40,5	46,1	32,0	36,5	35,1	35,4	38,5	35,3	49,4
Mazarron	-1,3788416	37,614572	40,3	37,8	39,4	18,3	43,0	48,1	33,8	38,4	37,7	37,7	40,7	37,7	58,6
Zalamea de la Serena	-5,6910276	38,678704	64,2	56,1	60,5	50,7	60,8	66,9	57,9	57,1	62,4	57,8	66,5	60,1	78,2
Monterrubio de la Serena	-5,3836134	38,591582	67,8	61,4	63,3	55,0	64,1	69,7	60,7	60,5	64,9	60,6	68,5	63,3	83,3
Don Benito	-5,9062469	38,930491	57,4	50,9	54,6	44,6	58,2	62,1	54,1	51,7	58,1	53,5	62,3	55,2	75,4
Villagonzalo	-6,1858738	38,837247	61,3	49,1	55,9	50,7	58,3	64,0	57,0	51,9	58,5	53,0	65,9	56,9	73,8
Jerez de los Caballeros	-6,7369024	38,281336	59,6	48,5	54,3	41,6	54,3	63,1	54,1	50,6	54,2	50,1	61,2	53,8	75,3
Olivenza	-7,0578251	38,720921	60,1	47,7	54,7	42,3	55,0	64,2	53,3	50,8	54,9	50,4	62,5	54,1	73,5
Villafranca de los Barros	-6,3485695	38,575591	62,9	51,5	57,8	46,1	57,8	66,4	58,0	54,3	59,0	54,4	64,3	57,5	74,7
Merida	-6,3192869	38,845149	61,8	49,8	56,3	50,1	58,6	65,1	57,9	52,8	58,6	53,0	65,3	57,2	75,7
Azuaga	-5,7077922	38,391445	68,9	63,0	64,9	57,1	65,4	70,8	62,9	62,2	66,7	62,0	70,1	64,9	81,2
Puebla de alcocer	-5,0955855	39,074643	67,1	62,4	63,6	56,9	67,0	69,9	63,3	60,8	66,0	61,6	68,9	64,3	78,9
Don Benito	-5,858992	38,984723	57,3	49,4	54,4	45,3	55,7	62,3	53,4	50,7	58,4	52,7	61,5	54,7	74,0
Badajoz	-6,827838	38,877039	59,4	47,4	54,5	48,2	56,8	63,3	55,6	50,0	57,1	50,9	63,8	55,2	73,4
Pueblonuevo del Guadiana	-6,7328012	38,912998	59,5	47,7	54,4	49,1	56,7	63,2	55,6	50,1	56,7	51,2	64,2	55,3	73,3
Lobon	-6,6655535	38,860185	60,5	48,5	54,1	48,7	57,2	62,8	56,4	50,4	57,0	50,7	64,6	55,5	72,7
Arroyo de San Servan	-6,4728164	38,858246	62,0	50,2	56,0	51,1	58,7	65,1	57,9	52,3	58,5	53,5	65,4	57,3	74,4
Villar de Reina	-5,742601	39,102295	61,9	56,3	59,1	50,6	61,6	65,6	57,2	54,8	62,6	57,0	65,4	59,3	78,9
Cartagena	-0,9508754	37,688833	36,4	35,3	35,4	14,2	39,6	45,8	31,3	36,1	33,9	34,8	37,2	34,5	58,8
Murcia	-1,1227711	37,831265	43,6	42,5	42,7	24,0	48,7	53,6	39,3	41,6	41,4	40,9	46,7	42,3	62,8
Fuente alamo	-1,1292626	37,748269	34,1	30,6	33,4	14,6	38,8	44,1	29,2	30,4	32,6	31,6	37,8	32,5	63,7
Cartagena	-1,070786	37,676671	34,1	31,0	33,1	13,9	38,7	44,5	29,3	31,4	31,2	31,2	38,0	32,4	60,0
Cartagena	-0,8037931	37,611152	35,4	34,5	34,9	15,3	40,3	46,1	31,1	34,7	34,4	35,8	37,9	34,6	58,7
Fuente alamo	-1,2380371	37,699008	42,3	38,9	40,9	22,2	45,8	51,3	36,8	37,8	39,0	38,0	44,8	39,8	63,2
Casatejada	-5,6781	39,867824	63,7	57,7	60,5	54,2	63,9	66,6	59,2	58,3	64,4	59,0	68,0	61,4	81,3
Aldehuela del Jerte	-6,2302346	40,008316	64,3	56,0	60,5	56,8	61,1	66,4	61,2	57,1	63,6	56,4	70,1	61,2	79,5
Moraleja	-6,6759606	40,046357	62,9	53,6	59,3	53,5	60,4	66,6	60,5	51,6	61,7	55,1	66,7	59,3	80,0
Coria	-6,5458096	39,978094	63,5	54,8	60,0	55,8	61,3	67,0	61,1	56,0	63,2	55,5	69,9	60,7	78,4
Madrigalejo	-5,5954391	39,135847	63,3	56,6	59,2	51,2	62,8	67,5	58,5	56,2	63,0	57,5	66,1	60,2	80,7
Valdesalor	-6,4785825	39,377191	64,8	54,6	60,0	53,3	62,0	69,1	61,1	55,0	62,5	56,8	67,7	60,6	80,3

Peraleda de la Mata	-5,4639595	39,861132	66,8	60,7	63,9	57,2	66,0	69,7	62,7	61,0	66,5	61,9	69,0	64,1	82,4
Tejeda de tietar	-5,8600359	39,960042	67,6	68,0	66,2	59,6	69,4	71,1	66,3	67,4	68,9	63,2	74,0	67,4	77,6
Casar de Palomero	-6,3056933	40,298554	76,0	70,8	73,1	70,1	73,5	77,9	73,1	68,2	74,4	68,6	77,4	73,0	84,5
Madroñera	-5,7623097	39,464885	74,2	70,5	71,1	65,6	73,9	76,6	70,9	68,8	73,6	68,4	74,8	71,7	84,2
Guadalupe	-5,3482094	39,387141	72,5	68,6	70,6	64,4	72,7	75,6	69,4	68,2	72,5	67,5	74,4	70,6	85,3
Alcantara	-6,8981123	39,746563	62,0	50,3	56,4	52,0	58,9	65,7	59,1	53,0	60,0	54,2	66,2	58,0	78,5
Jarandilla de la Vega	-5,6463392	40,101413	77,7	77,2	75,6	72,7	77,5	78,2	75,8	76,0	76,5	72,5	79,8	76,3	84,0
Gargantilla	-5,9414268	40,239041	79,2	79,8	77,6	75,5	78,6	80,2	77,4	76,8	77,3	73,3	79,5	77,8	84,8
Talayuela	-5,5642306	40,011198	68,0	67,1	66,6	60,0	69,0	71,2	66,5	67,6	68,4	63,3	73,6	67,4	81,1
Valdastillas	-5,8687982	40,141215	81,7	81,1	80,2	78,6	80,5	81,3	79,7	76,6	79,1	76,2	80,1	79,5	83,5
Cieza	-1,3097249	38,235442	59,8	58,5	58,3	44,7	62,0	67,3	54,5	56,4	56,6	55,0	61,4	57,7	64,4
Ulea	-1,2578423	38,191392	50,4	49,4	49,0	32,1	54,0	59,0	46,1	47,5	48,1	46,7	53,2	48,7	71,6
Cieza	-1,4963438	38,283888	52,9	50,7	51,3	35,9	55,3	59,9	47,2	48,6	49,6	47,1	54,6	50,3	61,1
Calasparra	-1,6947638	38,253487	54,8	51,3	52,3	36,8	55,6	61,0	48,8	49,2	50,6	48,2	55,7	51,3	72,8
Calasparra	-1,5850018	38,269499	50,5	47,7	48,4	33,6	52,8	57,5	45,0	46,1	48,0	46,0	53,1	48,1	73,3
Caravaca	-1,980057	38,043911	70,6	69,3	68,9	57,4	71,0	73,7	66,1	67,7	67,4	67,1	68,1	67,9	88,1
Cehegin	-1,6828994	38,110901	62,3	60,9	60,1	47,7	63,3	68,1	57,4	58,7	58,2	56,8	62,9	59,7	73,0
Moratalla	-1,813186	38,196653	66,0	63,7	64,5	46,5	66,9	69,8	60,4	61,6	62,3	60,9	62,6	62,3	79,0
Cehegin	-1,7798922	38,104477	67,6	65,7	65,8	49,5	68,1	70,7	62,5	63,7	63,8	62,6	64,5	64,0	76,3
Moratalla	-2,0961142	38,1145	77,5	79,0	76,5	74,6	78,3	78,7	76,7	77,2	75,9	75,4	75,7	76,9	87,1
Vall de Uxo	-0,2304536	39,795861	45,9	45,3	46,5	28,9	52,5	59,4	43,2	42,1	45,8	45,8	50,1	46,0	64,2
Onda	-0,2444114	39,954016	52,9	54,4	50,8	38,0	55,9	63,1	48,6	44,5	51,7	49,7	54,4	51,3	69,2
San Rafael del Rio	0,3675272	40,594077	52,6	54,7	51,5	40,2	56,8	65,2	48,4	45,4	51,4	49,2	55,2	51,9	75,5
Benicarlo	0,4014538	40,411511	44,9	47,0	43,3	31,6	51,1	58,7	41,2	37,9	44,6	42,8	48,6	44,7	67,6
Castellon	-0,1191495	39,989342	51,1	51,9	48,8	37,4	55,0	62,8	45,5	43,6	48,3	46,3	52,8	49,4	70,3
Burriana	-0,1057138	39,887849	41,4	40,9	41,5	23,5	48,3	54,8	39,0	37,6	41,2	41,4	46,4	41,4	66,9
Ribera de Cabanes	0,1464314	40,133934	51,5	51,6	49,0	38,1	56,1	64,1	46,3	43,7	49,3	47,8	52,8	50,0	67,4
Nules	-0,1683946	39,877237	43,6	43,7	45,0	26,5	51,3	57,2	41,5	40,8	45,3	44,7	48,6	44,4	65,4
Segorbe	-0,4830876	39,817295	61,1	61,7	61,5	47,0	65,0	70,6	58,5	57,7	60,4	59,8	62,5	60,5	74,4
Baza	-2,7677154	37,564477	61,9	64,3	59,6	49,7	61,2	65,6	54,2	60,3	56,7	53,8	63,0	59,1	83,9
Puebla de Don Fadrique	-2,3817176	37,876115	75,1	75,8	73,9	68,5	75,9	76,9	73,0	73,4	72,9	72,6	72,9	73,7	82,3
Loja	-4,138128	37,1693	60,9	55,3	58,4	52,2	59,1	66,1	56,3	56,2	58,1	56,7	63,1	58,4	79,5
Iznalloz	-3,5514591	37,416406	64,5	62,8	60,3	57,4	60,4	68,0	57,9	61,1	59,8	58,2	64,7	61,4	86,0

Jerez del Marquesado	-3,1498644	37,190536	70,0	70,2	69,6	67,1	69,4	72,6	66,9	68,5	68,3	67,2	69,9	69,1	84,3
Cadiar	-3,183988	36,923123	64,6	63,0	62,5	58,7	61,7	68,0	59,6	62,4	61,0	60,1	65,2	62,4	81,4
Zafarraya	-4,1538389	36,990314	74,5	74,2	74,3	72,9	73,7	78,1	72,4	73,9	73,9	73,2	75,9	74,3	89,9
Padul	-3,600317	37,018743	63,9	62,2	60,1	57,1	60,3	67,7	57,6	61,1	59,4	58,9	64,6	61,2	80,0
Granada	-3,6385645	37,172054	61,7	58,8	56,8	50,8	57,9	65,8	55,0	57,8	56,3	55,8	61,5	58,0	77,9
Almuñecar	-3,6790578	36,751942	45,5	40,3	40,6	31,7	44,1	52,7	39,1	41,7	41,4	40,2	47,2	42,2	54,1
Gibraleon	-7,0278022	37,318328	35,5	27,6	33,7	16,5	34,7	45,2	33,9	33,1	31,1	28,5	38,9	32,6	59,5
Lepe	-7,2430825	37,302685	36,3	28,0	34,3	17,1	36,4	46,9	32,4	33,0	32,7	29,8	38,9	33,3	57,0
Gibraleon	-7,059841	37,412354	41,2	34,0	38,5	22,4	40,8	51,1	38,2	37,6	36,6	34,1	44,3	38,1	66,0
Moguer	-6,7925285	37,14648	38,2	30,3	35,0	18,9	38,3	47,2	34,3	34,6	33,5	31,5	40,8	34,8	62,3
Niebla	-6,7353478	37,347125	38,9	31,0	37,1	19,7	38,1	48,6	35,9	34,9	35,0	32,2	42,7	35,8	62,5
Aroche	-6,9449915	37,958077	62,1	51,3	55,8	42,7	56,8	64,9	55,9	52,4	56,8	51,1	62,4	55,7	71,4
La puebla de Guzman	-7,2483655	37,552176	48,5	41,8	45,9	29,8	47,5	57,9	45,2	44,4	44,2	41,3	51,3	45,2	69,5
El Campillo	-6,5992719	37,660989	50,3	42,4	46,9	32,5	48,4	57,6	46,7	45,3	46,7	43,1	53,5	46,7	74,5
La Palma del Condado	-6,5415566	37,366968	44,8	37,5	42,7	25,8	44,7	54,3	42,2	41,5	41,3	37,8	48,1	41,9	66,3
Almonte	-6,4765444	37,148345	36,0	27,3	33,8	17,0	34,8	45,9	33,1	32,3	31,8	29,9	39,6	32,9	60,1
Valfarta	-0,1478858	41,531503	70,0	69,7	67,1	70,1	67,6	76,6	66,1	64,2	67,2	64,9	69,0	68,4	83,5
Zaidin	0,2890014	41,637169	68,3	66,6	65,5	68,9	66,5	75,4	64,5	63,2	65,7	63,2	67,5	66,8	79,4
Alcolea de Cinca	0,0731411	41,74095	69,9	68,5	66,7	70,2	66,6	75,0	65,3	64,6	66,9	65,1	68,1	67,9	82,2
Tanarite de Litera	0,3771357	41,780947	70,7	69,7	67,4	70,5	67,4	75,2	65,5	64,7	66,9	65,3	68,4	68,3	81,9
Lanaja	-0,337846	41,786429	72,2	70,5	68,4	71,8	69,3	77,8	68,6	65,7	69,5	67,1	71,4	70,2	83,5
Monzon	0,1273494	41,957687	73,3	72,7	70,3	72,0	70,4	77,6	69,2	68,6	69,6	68,2	70,4	71,1	83,1
Barbastro	0,1126102	42,013471	74,1	74,6	69,9	71,8	71,4	77,5	70,5	68,8	70,5	68,7	71,4	71,8	84,0
Sariñena	-0,1766614	41,771411	71,4	70,1	68,3	70,5	68,2	76,3	66,5	64,8	67,4	65,3	69,8	69,0	83,1
Huesca	-0,3777068	42,105429	74,8	74,2	71,9	73,8	71,2	78,7	71,0	69,7	71,8	69,7	72,0	72,6	83,6
Candasnos	0,094436	41,45994	66,8	65,7	65,3	67,3	64,6	74,4	63,1	61,9	63,8	62,2	66,5	65,6	83,7
Grañen	-0,3560041	41,942469	74,8	74,5	70,4	72,5	71,1	78,4	69,7	68,0	70,2	69,0	71,6	71,8	82,9
Huerto	-0,1365362	41,966019	75,1	75,2	71,7	73,5	71,9	78,6	71,8	69,1	71,4	69,2	73,1	72,8	83,6
Gurrea de Gallego	-0,7311994	41,992829	77,0	75,8	73,7	73,7	73,7	77,3	71,8	71,8	74,2	71,7	73,5	74,0	85,9
Alfantega	0,1477817	41,821958	73,3	72,9	69,7	71,5	69,9	77,6	69,2	67,6	69,8	68,3	70,2	70,9	82,1
Fraga	0,3539314	41,495165	64,9	63,7	62,4	66,8	63,2	73,1	60,9	59,4	62,9	60,4	64,6	63,8	76,9
Tardienta	-0,5075831	41,969367	72,8	72,1	69,6	73,0	69,5	78,3	68,4	66,4	69,5	67,4	70,9	70,7	83,5
San Esteban de Litera	0,3042037	41,882938	74,4	73,6	70,8	73,6	70,8	77,7	70,0	69,2	70,6	69,1	71,3	71,9	82,0

Belver de Cinca	0,2318291	41,742536	71,4	70,3	66,9	71,6	67,6	76,5	66,6	65,2	68,0	65,9	69,5	69,0	83,8
Alberuela de Tubo	-0,2573084	41,883957	71,9	71,1	68,1	70,8	68,6	76,2	67,5	65,8	68,0	65,5	70,2	69,4	84,5
Jumilla	-1,4232837	38,394834	62,8	61,9	60,5	49,1	64,4	68,6	57,8	60,0	58,9	57,4	64,5	60,5	77,7
Yecla	-1,1859032	38,658948	69,2	69,3	66,6	58,7	69,9	73,6	64,9	65,4	66,0	63,8	68,9	66,9	85,6
Yecla	-1,1125211	38,562731	64,3	63,9	62,7	53,0	65,7	70,4	59,5	61,5	60,7	58,8	65,1	62,3	83,7
Jumilla	-1,2407841	38,392588	64,4	63,6	62,8	52,7	65,6	71,1	59,3	61,3	60,8	59,3	65,8	62,4	73,6
Jumilla	-1,3242866	38,31972	56,4	54,6	53,9	39,4	58,4	63,8	50,6	52,3	52,4	50,8	57,6	53,7	74,7
Aitona	0,4609093	41,486913	65,1	63,4	61,4	65,8	62,9	73,5	60,2	58,9	61,6	58,8	65,0	63,3	77,7
Albesa	0,6705502	41,760356	67,2	69,8	65,9	70,1	65,5	74,9	66,8	65,2	65,8	64,7	70,2	67,8	82,6
Alcarras	0,5506119	41,56508	68,2	67,3	65,2	66,6	66,7	75,4	63,0	63,0	65,2	62,6	65,8	66,3	81,5
Alfarras	0,5780224	41,819488	69,2	70,5	67,7	72,7	66,7	75,7	68,4	66,4	67,7	66,5	71,2	69,4	83,0
Algerri	0,6483717	41,801036	69,9	72,3	68,6	73,4	68,4	76,7	70,1	68,5	69,0	67,7	71,9	70,6	80,0
Alguaire	0,5361346	41,742812	72,9	72,6	69,8	72,8	69,6	76,9	68,6	68,8	70,2	68,3	70,6	71,0	84,8
Castellnou de Seana	0,9520619	41,65659	67,0	68,7	66,0	70,1	65,5	74,5	66,0	64,6	66,6	64,9	70,3	67,7	82,6
Cervera	1,2967772	41,662217	75,0	76,8	73,9	78,3	72,3	79,0	74,7	73,1	73,5	72,5	75,0	74,9	83,6
Gimenells i el Pla de la Font	0,3933398	41,658132	71,1	69,8	68,8	70,0	68,2	75,7	65,9	65,2	67,2	66,0	68,7	68,8	83,2
Golmes	0,9248038	41,63641	65,9	67,8	65,2	67,7	65,1	74,4	65,6	63,2	66,2	64,5	69,3	66,8	75,0
Raimat	0,4490319	41,683272	72,4	71,8	69,0	71,7	69,1	77,2	67,9	66,8	68,6	67,2	70,1	70,2	83,1
Balaguer- Monasterio Avellanas	0,7613663	41,879114	72,2	73,5	70,9	74,1	69,5	77,8	72,2	69,6	70,1	69,2	72,8	72,0	83,3
El Canos	1,2041447	41,689385	73,6	75,5	72,5	77,0	71,0	78,8	73,0	71,1	71,7	70,8	74,1	73,5	84,6
El Poal	0,8777387	41,672786	65,8	67,8	65,2	67,6	65,1	74,4	65,6	63,2	66,2	64,5	69,3	66,8	81,3
Sant Marti de Riucorb	1,0885432	41,572353	69,8	71,7	70,0	73,7	67,5	77,3	70,1	67,7	69,5	68,4	71,8	70,7	85,1
Seros	0,4279758	41,463784	65,0	63,7	62,5	65,0	62,5	72,7	58,7	59,1	62,1	59,2	63,9	63,1	78,2
Tarrega	1,1626814	41,666945	70,1	72,0	69,6	74,6	67,7	76,4	69,2	68,5	69,6	68,1	72,4	70,7	80,5
Tornabous	1,0451011	41,68834	68,2	70,6	67,6	71,0	66,6	76,0	67,3	66,3	67,9	66,5	71,2	69,0	80,2
Vallfogona de Balaguer	0,8293888	41,784868	67,4	70,3	66,7	71,1	66,1	75,4	67,4	66,2	67,5	66,4	70,5	68,6	82,5
Vilanova de Segria	0,628392	41,714499	65,6	66,8	65,4	69,6	64,4	72,5	65,4	64,0	65,2	63,5	69,1	66,5	81,9
Lorca	-1,6294551	37,601733	52,0	50,7	51,8	32,1	54,3	58,8	45,5	52,0	49,4	48,7	52,3	49,8	72,8
Lorca	-1,6938893	37,50379	47,7	46,0	43,6	29,1	46,8	54,5	40,5	46,5	42,7	41,9	48,3	44,3	73,8
Agoncillo	-2,2904337	42,468182	80,2	81,5	77,8	76,7	79,3	80,5	77,3	77,9	78,6	76,9	80,0	78,8	89,7
Albelda de Iregua	-2,4718558	42,380733	81,0	81,4	78,4	78,9	79,5	80,3	79,5	78,6	79,3	78,2	80,1	79,6	90,3
Asenjo	-2,1533164	42,340952	81,0	82,2	78,7	78,9	79,8	81,2	78,8	78,8	80,0	78,1	81,2	79,9	91,1
Logroño	-2,5136369	42,43969	81,5	82,1	78,5	78,7	79,7	80,9	78,9	79,1	79,5	77,4	80,2	79,7	91,5

Santa Engracia del Juvera	-2,2629377	42,368971	80,8	81,5	78,3	78,1	79,8	80,8	78,6	78,7	79,8	77,3	80,8	79,5	86,3
Aldea Nueva de Ebro	-1,9048679	42,222598	77,4	79,3	75,9	73,1	77,4	78,8	74,8	74,7	76,4	74,2	78,6	76,4	86,4
Alfaro	-1,7776916	42,152119	79,4	80,6	76,7	70,1	78,5	78,7	77,0	76,7	77,2	74,9	77,6	77,0	88,6
Calahorra	-2,001826	42,334834	77,8	80,0	77,0	74,1	77,5	79,7	74,8	76,6	76,8	74,1	79,4	77,1	90,9
Corvera (Cabreton)	-1,8924542	42,006904	79,4	80,3	77,3	73,3	78,8	80,0	78,1	76,8	78,0	75,8	79,0	77,9	86,1
Igea	-1,9937535	42,05775	80,5	81,4	78,9	77,0	79,4	80,8	79,7	78,1	78,8	78,2	78,9	79,3	88,5
Quel	-2,037178	42,252488	79,2	80,8	77,1	76,3	78,7	80,1	77,0	76,9	78,4	75,5	79,9	78,2	85,4
Rincon de Soto	-1,8508464	42,251583	77,4	79,4	75,9	73,1	77,3	78,7	74,8	74,8	76,4	74,2	78,6	76,4	85,8
Aguilas	-1,5921627	37,418665	33,9	32,7	34,0	13,5	37,6	44,4	28,9	32,9	32,1	33,3	36,7	32,7	48,9
Lorca	-1,8177885	37,855634	69,2	68,2	68,5	54,0	70,7	72,5	65,2	66,9	65,8	66,2	66,4	66,7	83,5
Lorca	-1,623984	37,4878	48,2	47,0	47,4	26,9	51,1	57,7	42,7	48,8	45,3	46,1	47,6	46,3	59,9
Puerto Lumbreras	-1,7255508	37,590472	47,5	46,3	43,9	29,4	46,1	53,3	39,8	46,3	42,9	42,0	48,0	44,1	68,9
Mula	-1,4294482	38,065871	51,9	50,2	50,2	33,4	54,9	60,7	46,8	47,6	48,6	47,1	53,7	49,6	61,4
Mula	-1,46674	38,041031	53,7	51,9	52,3	37,2	56,3	62,2	48,4	49,5	51,2	49,7	56,9	51,7	60,6
Torres de Cotillas	-1,3025362	38,006971	41,8	38,3	40,2	21,5	45,8	50,6	36,2	36,4	39,0	38,0	44,4	39,3	64,2
Molina del Segura	-1,2206884	38,127483	43,4	39,6	42,6	23,7	46,3	52,8	37,9	38,3	40,7	40,0	46,5	41,1	62,5
Molina del Segura	-1,2336707	38,071139	41,5	38,0	40,2	20,5	44,4	50,4	36,1	37,2	38,6	37,6	44,3	39,0	64,6
Abanilla	-1,0655079	38,170041	41,6	38,8	40,3	20,8	44,9	50,9	36,9	37,6	37,9	37,7	43,7	39,2	64,5
Fortuna	-1,1526819	38,161028	42,4	39,2	40,4	22,6	45,4	50,5	36,7	37,9	38,9	37,5	45,6	39,7	63,7
Ojos	-1,3394287	38,113316	49,0	46,6	47,6	30,4	51,3	57,2	43,3	44,2	46,4	44,7	51,3	46,5	52,7
Beniel	-0,9997837	38,034507	39,4	36,0	38,1	19,3	44,0	49,3	34,3	35,5	36,4	35,5	42,7	37,3	63,2
Murcia	-1,2682702	37,898166	51,5	49,4	49,8	32,8	54,7	59,9	45,7	47,8	47,8	47,0	52,9	49,0	48,9
Murcia	-0,9840042	37,977528	41,8	39,4	40,9	21,3	45,7	52,3	36,9	38,4	38,6	38,1	44,7	39,8	56,4
Murcia	-1,1347189	37,940075	46,2	43,3	44,7	26,5	49,2	55,2	41,2	42,5	42,6	42,0	48,9	43,9	61,4
Fitero	-1,8426437	42,046077	80,3	81,5	77,6	73,2	79,3	80,0	78,3	77,6	78,6	76,4	79,1	78,4	85,4
Cascante	-1,7239555	42,034371	78,6	79,8	75,7	69,1	77,5	78,9	75,4	74,9	76,6	74,3	77,0	76,2	86,2
Ablitas	-1,6447131	41,996446	74,5	73,2	71,9	70,1	71,7	77,2	68,5	68,6	71,5	68,4	72,0	71,6	85,9
Murillo el fruto	-1,4871859	42,38498	77,5	79,1	76,0	74,7	76,9	80,2	74,2	75,7	76,2	74,4	77,4	76,6	84,5
Miranda de Arga	-1,8087315	42,511252	79,8	80,8	76,6	75,6	78,3	79,9	76,9	76,9	78,1	76,4	78,9	78,0	86,1
Falces	-1,7925482	42,409669	80,0	81,8	78,1	76,3	79,2	81,2	77,7	78,4	78,2	76,0	80,0	78,8	85,8
Bargota	-2,2992201	42,477657	80,2	81,5	77,8	76,7	79,3	80,5	77,3	77,9	78,6	76,9	80,0	78,8	85,7
Bardenas Reales	-1,5187546	42,295154	78,6	80,1	77,0	76,3	77,6	80,5	74,7	77,6	77,5	75,6	78,5	77,6	86,0
Los Arcos	-2,1845206	42,539308	81,0	81,8	78,3	78,5	80,1	80,8	78,8	78,7	79,4	77,2	80,8	79,6	86,9

Sesma	-2,126631	42,473409	81,0	81,9	77,8	77,8	79,6	81,1	78,7	78,9	79,4	78,0	80,7	79,5	90,1
Cadreita	-1,6556731	42,26433	76,4	77,2	75,1	72,6	76,0	79,1	72,1	75,0	75,4	72,8	76,5	75,3	82,6
Bardenas Reales	-1,7183027	42,207768	77,8	80,0	76,6	73,6	77,6	79,7	74,6	76,6	77,0	74,3	79,9	77,1	79,3
Sartaguda	-2,0512344	42,361948	78,5	80,8	77,5	74,8	78,7	79,8	76,6	77,5	77,6	75,1	79,6	77,9	78,2
Olite	-1,662579	42,423779	78,1	79,2	76,5	75,4	77,5	80,2	74,4	76,8	76,9	75,4	78,0	77,1	85,7
Murillo el Cuende	-1,6153521	42,361474	77,4	79,1	76,6	74,8	77,2	80,1	74,3	76,2	76,5	74,9	77,8	76,8	82,4
Corella	-1,8398436	42,115577	79,8	80,9	76,6	70,6	78,9	78,8	76,9	77,1	77,8	75,8	77,5	77,3	81,2
Funes	-1,8066789	42,287885	77,7	79,5	76,0	73,7	77,1	79,4	74,8	75,3	76,7	73,3	79,0	76,6	79,8
Lerin	-1,9763006	42,503595	80,1	80,9	77,5	76,8	79,2	80,2	77,4	77,7	78,3	76,8	79,8	78,6	87,3
Los Palacios y Villafranca	-5,9390554	37,179127	37,3	30,9	36,2	19,9	37,0	45,4	31,7	34,0	34,4	32,3	42,5	34,7	56,8
Las cabezas de San Juan	-5,884722	37,01556	36,6	29,8	34,5	19,2	35,6	44,4	30,3	33,8	33,5	32,1	41,0	33,7	59,2
Lebrija	-6,1261602	36,976641	34,6	28,1	34,1	16,6	34,6	43,4	30,2	32,5	32,2	30,6	39,4	32,4	63,1
Aznalcazar	-6,2733503	37,151795	38,8	31,5	38,0	20,4	39,4	50,3	37,7	37,2	35,7	33,2	43,6	36,9	62,9
Isla Mayor	-6,1512787	37,098521	36,2	29,9	34,9	19,2	36,1	45,1	30,8	33,8	33,0	31,8	40,7	33,8	58,0
La puebla del Rio	-6,1338321	37,226032	36,6	29,8	34,8	19,3	36,4	45,1	31,3	33,2	33,6	32,5	41,9	34,0	60,7
La puebla del Rio II	-6,0465691	37,080174	36,9	29,8	34,7	19,5	36,5	45,7	31,0	33,8	33,3	32,2	41,2	34,0	62,9
Ecija	-5,0770704	37,592934	46,1	37,4	40,7	33,1	42,9	52,9	37,9	39,2	41,3	40,3	47,8	41,8	66,3
La Luisiana	-5,2281407	37,525293	45,9	38,7	44,5	30,2	45,0	53,5	39,6	41,4	43,7	41,6	49,9	43,1	68,1
Carmona	-5,587615	37,400903	40,1	33,1	38,5	22,2	39,3	48,4	34,5	35,9	36,7	34,4	44,4	37,1	63,8
Osuna	-5,1348377	37,25503	44,2	37,0	42,3	28,5	43,6	52,6	38,5	41,4	41,9	40,1	48,2	41,7	64,9
La Rinconada	-5,924839	37,456832	37,0	30,0	35,5	21,1	36,0	45,2	30,7	33,4	34,0	32,7	42,8	34,4	64,9
Sanlucar La Mayor	-6,2550749	37,42179	42,4	34,9	41,0	23,1	42,4	51,7	40,6	39,9	39,3	35,3	46,3	39,7	66,6
Villanueva del Rio y Minas	-5,6840093	37,613036	39,3	31,5	37,3	22,5	38,7	47,3	33,9	35,8	36,1	34,0	44,0	36,4	59,9
Lora del Rio	-5,5407037	37,660906	44,0	37,0	42,3	26,9	42,7	51,4	38,6	41,2	41,0	38,7	48,5	41,1	65,9
Los Molares	-5,6729697	37,176152	39,8	32,7	38,0	22,0	38,7	47,5	33,3	36,4	36,4	34,4	43,0	36,6	63,9
Guillena	-6,06419	37,514568	39,4	32,4	36,8	22,4	37,7	45,8	32,8	35,8	35,5	33,5	43,5	36,0	64,0
Puebla Cazalla	-5,3509152	37,218131	46,0	38,1	43,3	28,7	44,3	52,5	39,4	42,2	43,1	40,5	49,6	42,5	62,6
Alcala del Rio	-5,9641033	37,512529	36,9	29,1	34,9	19,7	34,7	43,9	30,3	32,7	33,6	31,6	40,4	33,4	56,5
San Javier	-0,819705	37,791664	32,4	28,8	32,4	12,7	38,1	42,6	28,3	29,5	30,3	31,1	35,6	31,1	56,5
Torre Pacheco	-0,8985888	37,773803	31,0	27,3	29,8	12,1	36,0	41,1	26,6	28,5	28,6	28,2	34,5	29,4	63,7
San Javier	-0,8836862	37,848045	33,5	30,2	32,5	13,2	39,2	44,2	28,6	29,0	31,6	31,3	36,9	31,8	57,9
Torre Pacheco	-0,9316281	37,823827	35,1	31,6	34,6	14,8	39,6	45,2	30,6	31,2	33,0	32,1	38,7	33,3	59,6
Torre Pacheco	-0,9867861	37,74765	32,4	28,4	31,9	12,7	37,1	43,4	28,1	28,6	30,3	29,8	35,8	30,8	63,7

Pedralba	-0,7175861	39,567014	54,2	49,8	50,4	38,4	55,0	61,7	47,5	46,2	50,8	49,1	54,9	50,7	69,6
Liria	-0,627062	39,691055	54,6	49,6	48,9	40,3	55,1	60,5	48,1	47,0	50,8	49,4	54,6	50,8	73,5
Benifayo	-0,4618662	39,280627	38,5	39,2	39,0	22,1	46,4	53,4	37,1	35,8	40,0	39,3	45,6	39,7	60,1
Cheste	-0,7444395	39,518889	59,9	55,8	56,4	44,3	61,4	66,9	53,4	52,9	57,1	55,2	59,3	56,6	67,3
Tabernes de Valldigna	-0,2380292	39,095261	42,4	42,1	42,9	23,8	49,4	55,9	39,4	39,1	41,8	42,1	46,9	42,3	53,4
Villanueva de Castellon	-0,5242892	39,065567	41,8	42,1	41,9	24,4	49,0	55,3	40,1	38,8	42,4	41,3	47,3	42,2	64,4
Sagunto	-0,2663216	39,647534	43,1	42,4	43,3	25,2	50,1	56,1	40,5	39,7	43,5	43,6	48,2	43,2	63,2
Benavites	-0,2162186	39,730391	46,5	47,0	46,9	28,3	52,6	59,9	43,4	43,6	46,2	46,0	49,7	46,4	61,1
Moncada	-0,3989602	39,587729	44,3	44,3	44,9	25,9	51,3	57,9	41,7	40,5	44,6	44,2	48,6	44,4	64,9
Carcagente	-0,4461657	39,113604	40,2	40,9	40,3	22,7	48,1	53,9	38,0	37,6	40,8	41,0	46,6	40,9	65,5
Carlet	-0,5459462	39,22642	49,3	44,5	45,1	30,4	51,3	57,8	42,6	42,4	46,3	44,0	51,4	45,9	78,8
Luchente	-0,3600825	38,938508	56,9	56,9	57,9	41,9	61,6	68,3	53,8	53,8	56,2	56,5	59,3	56,7	89,7
Requena	-1,2323883	39,504667	74,7	74,3	72,4	70,3	74,7	77,0	71,9	70,0	72,5	71,8	73,1	73,0	62,1
Algemesi	-0,4353656	39,216442	38,4	39,0	39,4	20,6	46,8	53,0	36,8	35,7	39,9	39,8	44,6	39,5	66,7
Campo Arcis	-1,1622154	39,433357	73,5	72,0	70,6	68,3	72,8	76,1	70,8	68,1	70,9	70,1	72,0	71,4	64,2
Betera	-0,4685258	39,597708	46,8	47,0	46,9	29,6	52,6	59,4	43,9	42,7	46,2	46,2	51,6	46,6	68,2
Picasent	-0,4976324	39,362484	44,0	45,1	45,2	26,9	51,4	57,8	42,0	40,8	45,0	44,5	49,9	44,8	66,6
Montesa	-0,6383798	38,954502	58,9	55,1	55,7	42,4	59,6	65,0	52,6	51,9	55,5	54,1	57,7	55,3	57,5
Jativa	-0,5497109	38,998803	44,3	45,5	46,0	27,6	52,5	58,0	42,3	42,6	45,6	45,7	50,1	45,5	83,4
Villalonga	-0,2042579	38,892111	44,3	43,5	44,8	27,2	51,7	57,4	41,4	41,0	45,0	45,6	49,6	44,7	61,9
Gandia	-0,2506841	38,964297	49,2	48,5	49,2	32,0	55,0	61,4	45,5	45,0	48,5	48,8	53,0	48,7	60,7
Bolbaite	-0,6901658	39,069153	60,0	56,4	56,1	43,6	60,7	66,6	54,1	52,1	56,5	54,0	58,6	56,2	70,2
Chulilla	-0,8322414	39,67678	60,9	56,0	56,3	46,6	61,4	66,7	54,0	53,6	56,7	54,8	60,6	57,1	73,1
Almoacid de la Sierra	-1,3299642	41,452078	74,4	73,7	71,3	69,6	71,4	76,6	69,4	68,1	71,0	67,5	71,0	71,3	83,0
Belchite	-0,7216155	41,350306	72,1	71,4	69,8	67,3	69,3	75,5	65,7	66,1	69,3	66,7	69,0	69,3	85,1
Quinto	-0,5186373	41,388348	65,2	61,8	62,1	67,7	60,9	72,1	58,5	58,0	62,3	59,4	65,0	63,0	81,7
Fabara	0,1540344	41,167877	64,7	62,7	61,8	64,9	62,1	72,8	58,3	58,0	62,7	60,3	64,5	63,0	81,1
Epila	-1,2820466	41,583234	72,6	70,1	69,2	65,8	68,6	74,9	65,9	65,5	68,2	65,0	69,3	68,6	83,5
Ejea de los Caballeros	-1,1961298	42,097715	76,7	75,7	73,7	73,5	74,2	78,1	72,3	71,7	74,5	72,1	74,6	74,3	85,8
Sabada	-1,309387	42,267312	77,0	79,1	75,6	74,8	77,0	79,7	73,3	75,4	76,6	74,5	77,6	76,4	90,3
Luna	-0,9359498	42,095761	78,3	77,8	75,5	75,1	75,4	78,5	74,2	73,8	76,4	73,9	76,0	75,9	89,1
Santa Engracia	-1,3305062	41,921246	73,2	70,9	70,0	68,8	70,4	75,5	67,8	67,8	70,3	68,3	70,7	70,3	85,7
Pastriz	-0,7461599	41,59514	72,6	71,6	69,6	68,1	68,6	75,5	65,4	67,3	69,5	66,2	70,0	69,5	81,1

Zaragoza	-0,823819	41,71363	74,9	73,4	71,1	70,8	70,5	75,9	68,6	69,2	71,3	68,5	71,5	71,4	80,4
Calatayud	-1,6583593	41,332167	77,7	78,2	75,0	74,9	75,5	77,4	75,6	74,4	75,7	73,6	74,7	75,7	87,5
Borja	-1,5076903	41,855146	74,5	74,5	71,9	70,6	72,5	76,9	69,3	69,3	72,0	69,1	72,4	72,1	86,2
Tarazona	-1,7458841	41,916177	80,5	82,1	78,8	74,1	80,1	80,7	78,8	78,2	79,7	77,5	79,6	79,1	84,8
Caspe	-0,0710973	41,303968	61,0	59,4	60,5	63,8	59,2	70,9	55,7	56,4	59,7	57,1	61,7	60,5	80,9
Osera de Ebro	-0,5363755	41,545081	68,3	65,8	65,1	68,5	65,1	75,4	62,4	61,3	65,3	62,2	66,1	66,0	81,1
Daroca	-1,4247095	41,108073	78,3	78,6	76,4	77,6	77,3	77,2	78,5	75,8	76,3	76,0	77,1	77,2	86,7
Zuera	-0,7511391	41,869389	75,1	74,1	72,4	71,9	72,3	76,5	70,3	70,0	72,7	70,3	72,6	72,6	84,8
El Bayo	-1,2487734	42,175713	77,1	76,2	74,1	73,4	74,8	78,5	73,7	72,5	74,8	72,2	75,0	74,7	86,4
Tauste	-1,1428386	42,00023	73,5	71,6	70,1	69,6	69,9	75,7	67,8	66,8	70,7	67,2	70,8	70,3	85,9
Boquiñeni	-1,2496818	41,843217	73,7	71,7	70,6	69,3	69,9	75,9	67,1	66,8	70,3	66,7	70,0	70,2	84,7
