# Supplementary material for: Agroclimatic Metrics for the Main Stone Fruit Producing Areas in Spain in Current and Future Climate Change Scenarios: Implications From an Adaptive Point of View
Source: Front Plant Sci. 2022 Jun 8;13:842628. doi: 10.3389/fpls.2022.842628 (PMC9213681; doi:10.3389/fpls.2022.842628)
Supplement: Supplementary file 5 [file Data_Sheet_5.PDF]

Supplementary Table 5. Mean accumulated GDHs (1st January - Beginning April) for 2025-2045, RCP 4.5 at each location.

The last column shows the heat accumulation for the current situation, for comparison purposes

M1: bcc-csm1-1-m; **M2:** BNU-ESM; **M3:** CanESM2; **M4:** CMCC-CM; **M5:** GFDL-ESM2G; **M6:** inmcm4

M7: IPSL-CM5A-LR; **M8:** MIROC-ESM; **M9:** MPI-ESM-LR; **M10:** MPI-ESM-MR; **M11:** MRI-CGCM3

Municipality	Longitude	Latitude	M1	M2	M3	M4	M5	M6	M7	M8	M9	M10	M11	MEAN	CURRENT
Campo de Mirra	-0,7729762	38,679366	10379	10857	10061	13526	9939	9481	11559	12369	10285	11894	10733	11008	9222
Villajoyosa	-0,2561866	38,527917	21680	22126	21183	25594	20300	20412	22077	23791	21734	22510	21447	22078	19022
Ondara	0,0065631	38,818581	21336	21426	20881	25236	19712	19686	21298	22884	21116	22135	20605	21483	17381
Denia Gata	0,082579	38,792724	20304	20455	19686	24292	18928	18842	20543	22015	20266	21191	19951	20588	16297
Pinoso	-1,060721	38,427413	11556	12029	11206	15074	11060	10697	12688	13530	11568	13174	11685	12206	10446
Monforte del Cid	-0,7303963	38,398862	19053	19794	18818	23618	18546	18370	20053	21431	19345	20676	19115	19893	15732
Crevillente	-0,7831581	38,240831	21550	22318	21288	26087	21017	20836	22367	23668	21947	23150	21527	22341	20193
Almoradi	-0,7745396	38,031431	21839	22515	21166	26281	21106	21080	22449	23896	22003	23241	21693	22479	19077
Callosa de Sarria	-0,1044988	38,650249	17488	17634	16841	21258	16085	15923	17857	19085	17333	18469	17247	17747	19540
Pilar de la Horadada	-0,8125284	37,868588	22779	23593	22227	27138	22175	21971	23557	25014	23059	24280	22714	23501	19235
Catral	-0,8055704	38,153124	21892	22651	21392	26480	21346	21263	22725	24103	22154	23361	21874	22658	17734
Altea	-0,0795078	38,603643	19879	20022	19182	23919	18399	18382	20131	21438	19835	20823	19684	20154	18704
Planes	-0,3529076	38,78476	13438	13638	12934	17120	12374	12136	14193	15294	13281	14573	13539	13865	12544
Villena	-0,8753684	38,595491	14058	14629	13834	17904	13623	13237	15225	16191	14130	15639	14124	14781	10401
Agost	-0,6498214	38,421512	17881	18660	17607	22285	17422	17051	18948	20199	18115	19520	18038	18702	14888
Almansa	-1,1075837	38,903228	7977	8755	8049	11325	7751	7441	9153	9996	8234	9725	8667	8825	8608
Ontur	-1,4957688	38,622866	12637	13236	12281	16597	12378	12023	13941	14777	12492	14169	12753	13390	9229
Caudete	-0,9798818	38,734665	11887	12563	11464	15545	11610	11138	13174	14059	11820	13465	12132	12623	10377
La Mojonera	-2,7043824	36,787318	21596	21696	21264	25533	20799	20897	22806	22911	21811	23229	21760	22209	20088
Almeria	-2,4024534	36,835404	26074	26253	25503	29511	25309	25298	27043	27524	26276	27460	26261	26592	22117
Nijar	-2,1580794	36,95057	23025	23211	22674	26935	22363	22221	24155	24536	23293	24619	23067	23645	17941
Tabernas	-2,3023755	37,091315	17257	17383	17088	21058	16831	16536	18834	18833	17614	19109	17371	17992	13428
Fiñana	-2,8388277	37,156718	9891	10810	10132	11952	9640	9169	11450	11905	9993	11442	10252	10603	9292
Cuevas de Almanzora	-1,7704017	37,389125	21215	21184	21245	25132	20610	20412	22358	22601	21621	22943	21374	21881	17179
Huercal-overa	-1,8842832	37,412428	19029	19099	19114	22839	18619	18212	20290	20590	19468	20812	19169	19749	14947
Cuevas de Almanzora	-1,800522	37,256757	24567	24590	24261	28225	23686	23553	25522	25839	24836	26113	24537	25066	18782
Adra	-2,9923491	36,746758	24219	25020	24479	26813	23450	23223	25051	26091	24817	25728	23976	24806	9714
Tijola	-2,457021	37,371918	14084	14246	13870	17812	13653	13262	15844	15437	14153	15885	14416	14788	21485

Totana	-1,5130934	37,732459	19552	20432	19306	23970	19271	19111	20575	21896	19813	21136	19554	20420	15888
Alhama	-1,4167602	37,7922	19621	20430	19218	24120	19351	19039	20624	22040	19772	21141	19522	20444	15937
Librilla	-1,3382889	37,899373	18628	19257	18269	22790	17973	17938	19493	20768	18774	20110	18461	19315	16941
Mazarron	-1,4009916	37,56215	22049	22085	21352	26395	21078	21240	22580	23680	22063	23096	22091	22519	19904
Mazarron	-1,3788416	37,614572	21097	21389	20694	25330	20196	20362	21746	22890	21333	22335	21291	21697	17834
Zalamea de la Serena	-5,6910276	38,678704	14032	16368	13854	17843	13920	13819	15703	17087	13997	15683	13879	15108	11553
Monterrubio de la Serena	-5,3836134	38,591582	12788	14951	12600	16516	12892	12704	14668	15707	12816	14502	12813	13905	10402
Don Benito	-5,9062469	38,930491	16579	18196	16477	19876	16037	16142	17338	19068	16121	17438	15681	17177	11831
Villagonzalo	-6,1858738	38,837247	15711	18147	15876	18690	15689	15706	16614	18796	15879	17482	14872	16678	12917
Jerez de los Caballeros	-6,7369024	38,281336	15777	18162	15626	20116	15855	15611	17183	18617	16218	17907	15590	16969	12068
Olivenza	-7,0578251	38,720921	15872	18271	15821	20066	15944	15628	17148	18764	16647	18193	15481	17076	12264
Villafranca de los Barros	-6,3485695	38,575591	14720	17282	14659	18963	14995	14639	16267	17925	15149	16861	14729	16017	11971
Merida	-6,3192869	38,845149	15920	17984	15939	18787	15715	15772	16726	18782	15844	17510	15028	16728	12588
Azuaga	-5,7077922	38,391445	12626	14908	12477	16436	12838	12572	14617	15572	12523	14355	12703	13784	8697
Puebla de alcocer	-5,0955855	39,074643	13417	14992	13441	16767	13137	13037	14684	16015	13094	14687	13150	14220	11005
Don Benito	-5,858992	38,984723	16520	18434	16383	19782	16066	15953	17464	19147	15979	17466	15713	17173	11755
Badajoz	-6,827838	38,877039	16364	18469	16306	19186	16090	16039	17094	19253	16263	17809	15329	17109	12939
Pueblonuevo del Guadiana	-6,7328012	38,912998	16276	18326	16402	19204	16015	16110	17079	19090	16313	17840	15407	17097	13133
Lobon	-6,6655535	38,860185	16251	18355	16495	19249	16129	16333	17069	19084	16486	17951	15330	17157	13035
Arroyo de San Servan	-6,4728164	38,858246	15666	17744	15730	18644	15502	15523	16452	18481	15714	17289	14873	16511	13290
Villar de Reina	-5,742601	39,102295	15169	16760	15194	18551	14910	14821	16213	17888	14788	16347	14665	15937	11883
Cartagena	-0,9508754	37,688833	22236	22233	21808	26780	21330	21521	22753	23853	22475	23312	22491	22799	17708
Murcia	-1,1227711	37,831265	19870	20254	19299	24064	18905	18943	20477	21652	19731	21096	19552	20349	17019
Fuente alamo	-1,1292626	37,748269	22232	23022	21679	26677	21627	21674	22956	24495	22378	23578	22113	22948	17111
Cartagena	-1,070786	37,676671	22421	23083	21966	26916	21739	21622	23114	24523	22733	23923	22165	23109	18270
Cartagena	-0,8037931	37,611152	22053	22055	21645	26208	20952	21087	22470	23671	22029	22818	22242	22476	18375
Fuente alamo	-1,2380371	37,699008	20196	20998	19785	24698	19614	19576	21110	22475	20512	21764	20157	20989	16850
Casatejada	-5,6781	39,867824	14629	16541	14807	17812	14410	14442	15933	17279	14383	15932	14046	15474	10709
Aldehuela del Jerte	-6,2302346	40,008316	15240	17051	15632	17719	15308	15205	15940	17394	14916	16696	14133	15930	11790
Moraleja	-6,6759606	40,046357	15209	17124	15250	18106	15115	14987	15552	18232	14916	16596	14275	15942	11406
Coria	-6,5458096	39,978094	15435	17240	15527	17844	15281	15198	15885	17687	14852	16696	14173	15983	11523
Madrigalejo	-5,5954391	39,135847	14960	16943	15050	18396	14698	14625	16104	17789	14665	16216	14603	15823	11419
Valdesalor	-6,4785825	39,377191	14710	16802	14640	17963	14748	14474	15586	17734	14673	16251	14255	15622	9862

Peraleda de la Mata	-5,4639595	39,861132	14007	15885	14079	17075	13858	13669	15017	16534	13731	15213	13556	14784	10370
Tejeda de tietar	-5,8600359	39,960042	13911	14661	14223	16861	13223	13657	14466	14827	13223	15010	12753	14256	10910
Casar de Palomero	-6,3056933	40,298554	10421	12119	10580	13293	10723	10333	11556	13488	10048	12048	10267	11352	8771
Madroñera	-5,7623097	39,464885	11030	12909	11124	14532	11155	10686	12743	13893	10612	12614	11350	12059	9024
Guadalupe	-5,3482094	39,387141	11443	13333	11355	14672	11281	11041	12972	14081	10873	12878	11298	12293	8835
Alcantara	-6,8981123	39,746563	15869	17935	15944	18848	15780	15688	16597	18863	15822	17344	15109	16709	11894
Jarandilla de la Vega	-5,6463392	40,101413	9939	11237	10558	12972	9893	10014	11374	11638	9633	11624	9917	10800	9619
Gargantilla	-5,9414268	40,239041	8381	9350	8381	11316	8263	8067	9813	9933	7861	10027	8490	9080	10237
Talayuela	-5,5642306	40,011198	13404	14268	13785	16401	12779	13146	14116	14577	12832	14714	12511	13866	11239
Valdastillas	-5,8687982	40,141215	6825	8446	7174	9815	7215	6976	8532	9588	6594	8649	7773	7962	10911
Cieza	-1,3097249	38,235442	15050	15639	14801	19267	14599	14302	16190	17190	15185	16766	15021	15819	16973
Ulea	-1,2578423	38,191392	17702	18096	17340	22099	17007	16892	18576	19700	17726	19183	17502	18347	16196
Cieza	-1,4963438	38,283888	16918	17722	16750	21423	16689	16477	18113	19224	17220	18685	16938	17833	15774
Calasparra	-1,6947638	38,253487	16532	17464	16367	21051	16449	16130	17913	19141	16885	18324	16572	17530	14071
Calasparra	-1,5850018	38,269499	17372	17983	17065	21683	16793	16780	18277	19425	17444	18879	17131	18076	15712
Caravaca	-1,980057	38,043911	10631	11654	10003	15297	10049	10199	12044	12732	10523	12111	11612	11532	7108
Cehegin	-1,6828994	38,110901	14294	15009	14097	18424	14014	13665	15524	16545	14484	16061	14349	15133	11849
Moratalla	-1,813186	38,196653	12905	13791	12148	18539	12178	12501	14214	15085	12886	14428	13855	13866	12201
Cehegin	-1,7798922	38,104477	12402	13293	11740	17804	11699	11967	13688	14546	12412	13923	13311	13344	12661
Moratalla	-2,0961142	38,1145	5988	6660	5390	9138	5563	5343	7341	7767	5608	7121	7001	6629	6230
Vall de Uxo	-0,2304536	39,795861	19053	19054	18522	22606	17675	17325	19058	20844	18661	19752	18332	19171	16062
Onda	-0,2444114	39,954016	17440	17537	17734	20862	16366	16355	18166	19763	17146	18511	17154	17912	15457
San Rafael del Rio	0,3675272	40,594077	17451	17341	17488	20469	16508	16097	18120	19885	17335	18740	16995	17857	12433
Benicarlo	0,4014538	40,411511	19650	19670	19754	22568	18353	18060	20188	22042	19414	20751	19031	19953	15350
Castellon	-0,1191495	39,989342	18102	18168	18355	21192	16990	16688	18769	20327	18141	19457	17570	18523	14403
Burriana	-0,1057138	39,887849	20305	20251	19783	23887	18866	18678	20300	22041	20028	21075	19503	20429	14492
Ribera de Cabanes	0,1464314	40,133934	17900	17910	18015	20840	16593	16391	18561	20238	17828	19066	17533	18261	15515
Nules	-0,1683946	39,877237	19706	19434	19003	23121	17976	17852	19500	21126	19025	20126	18854	19611	15342
Segorbe	-0,4830876	39,817295	14475	14336	13789	17924	13199	12918	14661	16046	13867	15202	14105	14593	12538
Baza	-2,7677154	37,564477	13848	13924	13732	18182	13672	13323	15900	15412	14209	16058	14447	14792	8746
Puebla de Don Fadrique	-2,3817176	37,876115	7674	8559	7147	11689	7353	7344	9167	9623	7353	9010	8746	8515	6059
Loja	-4,138128	37,1693	14875	16608	14827	17795	14528	14219	16281	17637	15208	16545	14710	15748	11528
Iznalloz	-3,5514591	37,416406	13269	14619	13976	16322	13509	12707	15523	16180	13952	15537	13722	14483	7813

Jerez del Marquesado	-3,1498644	37,190536	10089	11036	10355	12363	10016	9508	11861	12336	10242	11695	10504	10910	6034
Cadiar	-3,183988	36,923123	13242	14342	13777	15787	13121	12595	15028	15573	13649	15091	13496	14155	8834
Zafarraya	-4,1538389	36,990314	9748	10934	9622	11918	9396	9133	11238	11834	9607	11114	10018	10415	6981
Padul	-3,600317	37,018743	13785	15080	14587	16600	13994	13230	15905	16570	14424	16030	14048	14932	10682
Granada	-3,6385645	37,172054	14415	15717	15245	17753	14635	13751	16455	17320	15265	16799	14732	15644	9930
Almuñecar	-3,6790578	36,751942	19972	20959	20341	22603	19457	19073	21254	21994	20341	21583	19855	20676	19144
Gibraleon	-7,0278022	37,318328	22670	24133	22224	26252	22384	21789	23031	24802	23255	24286	22035	23351	17580
Lepe	-7,2430825	37,302685	22300	23567	21772	25776	21643	21143	22959	24355	22650	23868	21757	22890	18243
Gibraleon	-7,059841	37,412354	21074	22469	20805	24622	20833	20205	21959	23379	21538	22724	20631	21840	16505
Moguer	-6,7925285	37,14648	21919	23395	21589	25730	21576	21080	22803	24153	22419	23598	21434	22700	16529
Niebla	-6,7353478	37,347125	21429	23002	20980	25126	21036	20590	22148	23673	21799	22997	20807	22144	15475
Aroche	-6,9449915	37,958077	15521	17934	15579	19841	15824	15433	17302	18456	15818	17774	15550	16821	13062
La puebla de Guzman	-7,2483655	37,552176	18998	20530	18738	22650	18968	18203	20211	21552	19454	20798	18757	19896	14447
El Campillo	-6,5992719	37,660989	18411	19990	17963	22043	18290	17665	19512	20894	18578	20119	18199	19242	14634
La Palma del Condado	-6,5415566	37,366968	20006	21549	19488	23649	19604	19020	20846	22262	20168	21518	19450	20687	16323
Almonte	-6,4765444	37,148345	22241	23786	21868	25808	21796	21234	22932	24400	22720	23845	21651	22935	17632
Valfarta	-0,1478858	41,531503	11859	11301	11555	12908	10577	10047	12222	13506	12157	13470	11243	11895	7854
Zaidin	0,2890014	41,637169	12428	11903	12097	13367	11048	10581	12578	13911	12790	14007	11696	12401	8991
Alcolea de Cinca	0,0731411	41,74095	11735	11245	11701	12773	10785	10259	12220	13443	12235	13370	11343	11919	9264
Tanarite de Litera	0,3771357	41,780947	11238	10902	11001	12426	10331	10002	11962	13259	11965	13149	11233	11588	8032
Lanaja	-0,337846	41,786429	10846	10590	10887	11979	10030	9465	11328	12862	11154	12347	10442	11084	7542
Monzon	0,1273494	41,957687	10240	9839	9869	11544	9435	8845	10866	12058	11011	12253	10153	10556	7986
Barbastro	0,1126102	42,013471	10152	9704	10052	11492	9450	8905	10757	12120	10671	11921	10004	10475	7606
Sariñena	-0,1766614	41,771411	11417	11070	11264	12723	10646	9965	12091	13268	12038	13265	11249	11727	8429
Huesca	-0,3777068	42,105429	9778	9537	9393	10951	9099	8418	10563	11685	10210	11558	9947	10104	7354
Candasnos	0,094436	41,45994	13067	12497	12430	14049	11716	11260	13198	14464	13251	14468	12333	12976	8357
Grañen	-0,3560041	41,942469	9823	9484	9744	11417	9340	8475	10743	11939	10523	11766	9969	10293	7471
Huerto	-0,1365362	41,966019	9696	9386	9725	11364	9145	8478	10528	11957	10471	11798	9778	10211	7532
Gurrea de Gallego	-0,7311994	41,992829	9047	9111	9240	11951	9941	8828	10771	12001	10439	11935	10080	10304	7380
Alfantega	0,1477817	41,821958	10151	9697	10105	11745	9516	8993	10993	12180	10935	12164	10319	10618	8660
Fraga	0,3539314	41,495165	13575	12983	13395	14328	12315	11816	13905	14929	13922	15097	13031	13572	10630
Tardienta	-0,5075831	41,969367	10719	10345	10458	11680	10077	9220	11357	12619	11142	12357	10423	10945	7498
San Esteban de Litera	0,3042037	41,882938	9969	9680	9773	11030	9166	8717	10687	11879	10610	11835	10045	10308	7885

Belver de Cinca	0,2318291	41,742536	11399	10908	11298	12141	10280	9762	12088	12844	11895	13126	10978	11520	8268
Alberuela de Tubo	-0,2573084	41,883957	11211	10768	11018	12402	10267	9625	11725	12960	11669	12883	10851	11398	8239
Jumilla	-1,4232837	38,394834	14022	14421	13862	18009	13559	13355	15138	16015	14042	15694	14018	14740	12831
Yecla	-1,1859032	38,658948	11769	12203	11546	15448	11430	11027	13001	13826	11643	13346	11992	12476	9356
Yecla	-1,1125211	38,562731	13136	13621	12865	16991	12725	12328	14322	15207	13189	14788	13232	13855	10386
Jumilla	-1,2407841	38,392588	13257	13823	13006	17258	12807	12564	14535	15430	13320	14938	13350	14026	14005
Jumilla	-1,3242866	38,31972	15838	16552	15626	20243	15483	15205	17088	18136	16055	17586	15876	16699	14101
Aitona	0,4609093	41,486913	13840	13431	13867	14757	12566	12117	14151	15371	14429	15581	13126	13931	10472
Albesa	0,6705502	41,760356	12111	11389	11477	12785	11445	10252	11833	13164	11652	12801	10562	11770	7850
Alcarras	0,5506119	41,56508	12608	11867	12310	13954	11247	10953	13088	14285	13215	14446	12355	12757	9669
Alfarras	0,5780224	41,819488	11127	10573	10539	11896	10612	9452	11079	12262	10749	11965	9886	10922	7900
Algerri	0,6483717	41,801036	11060	10337	10410	11731	10242	9291	10898	11944	10535	11803	9815	10733	7835
Alguaire	0,5361346	41,742812	10251	9841	9854	11305	9328	8849	10945	12025	10851	12125	10296	10516	7300
Castellnou de Seana	0,9520619	41,65659	12273	11560	11463	13025	11208	10225	11867	13161	11629	12907	10799	11829	8101
Cervera	1,2967772	41,662217	8507	8180	7826	9335	7917	6944	8737	9653	8009	9308	7817	8385	5778
Gimenells i el Pla de la Font	0,3933398	41,658132	11491	10919	11153	12592	10300	9966	11912	13193	12005	13236	11080	11622	8217
Golmes	0,9248038	41,63641	12593	11780	11890	13458	11882	10698	12190	13632	11923	13079	10942	12188	7850
Raimat	0,4490319	41,683272	10713	10310	10666	12020	9891	9323	11524	12587	11373	12632	10611	11059	7949
Balaguer- Monasterio Avellanas	0,7613663	41,879114	10132	9536	9447	11024	9599	8419	9977	11199	9705	10931	9089	9914	6546
El Canos	1,2041447	41,689385	9366	8897	8756	10149	8684	7655	9438	10533	8876	10227	8526	9192	6424
El Poal	0,8777387	41,672786	12592	11779	11889	13458	11881	10697	12189	13631	11922	13079	10941	12187	7942
Sant Marti de Riucorb	1,0885432	41,572353	10686	10091	9728	11396	9996	8828	10538	11633	10089	11312	9566	10351	7173
Seros	0,4279758	41,463784	13551	13193	13436	14865	12368	11986	14222	15481	14395	15728	13241	13861	10702
Tarrega	1,1626814	41,666945	10417	9915	9663	11070	9629	8601	10447	11460	9857	11186	9355	10145	7631
Tornabous	1,0451011	41,68834	11521	10969	10890	12301	10881	9610	11401	12614	10964	12231	10041	11220	7694
Vallfogona de Balaguer	0,8293888	41,784868	11725	10982	10981	12336	10768	9670	11425	12531	11144	12380	10220	11288	8188
Vilanova de Segria	0,628392	41,714499	12689	11939	11965	13339	11829	10864	12439	13567	12201	13382	11210	12311	8126
Lorca	-1,6294551	37,601733	17460	17628	17077	21807	16792	16810	18356	18764	17516	18682	17793	18062	14349
Lorca	-1,6938893	37,50379	18710	18840	18818	22588	18346	17861	20114	20242	19261	20588	18932	19482	14445
Agoncillo	-2,2904337	42,468182	7812	7574	7519	10914	7638	7305	9076	9453	8130	9724	7845	8454	7321
Albelda de Iregua	-2,4718558	42,380733	6910	6809	6726	9810	6977	6344	8088	8654	7091	8663	7132	7564	6251
Asenjo	-2,1533164	42,340952	7339	7317	7113	10440	7380	6789	8876	9280	7657	9257	7295	8068	6115
Logroño	-2,5136369	42,43969	7317	7190	6994	10408	7344	6780	8786	9037	7830	9326	7405	8038	6630

Santa Engracia del Juvera	-2,2629377	42,368971	7581	7321	7181	10660	7542	6969	8984	9278	7860	9457	7537	8215	5469
Aldea Nueva de Ebro	-1,9048679	42,222598	9394	8929	8921	12753	9171	8610	10866	11061	9739	11345	8999	9981	7993
Alfaro	-1,7776916	42,152119	8338	8006	8491	13571	8969	8296	10066	10096	9279	11078	9484	9607	7539
Calahorra	-2,001826	42,334834	9122	8793	8683	12506	9065	8442	10669	10904	9578	11152	8817	9794	7768
Corvera (Cabreton)	-1,8924542	42,006904	7334	7133	7238	11867	7793	7064	8848	9103	7920	9696	8239	8385	7174
Igea	-1,9937535	42,05775	6093	6237	6018	10051	6423	5889	7436	7993	6516	8209	7103	7088	6635
Quel	-2,037178	42,252488	8191	8131	7984	11424	8291	7731	9815	10285	8632	10192	8189	8988	6982
Rincon de Soto	-1,8508464	42,251583	9394	8929	8921	12753	9171	8610	10866	11060	9738	11345	8998	9980	7998
Aguilas	-1,5921627	37,418665	22815	22972	22342	26797	21827	21953	23322	24703	22946	23885	22742	23300	20895
Lorca	-1,8177885	37,855634	11729	12551	10999	16607	10863	11173	12911	13656	11607	13135	12590	12529	10245
Lorca	-1,623984	37,4878	19055	19015	18497	23428	18123	17998	19617	20351	19085	20123	19396	19517	17680
Puerto Lumbreras	-1,7255508	37,590472	18659	18791	18703	22451	18318	17969	20023	20206	19168	20505	18836	19421	14926
Mula	-1,4294482	38,065871	17504	18127	17273	21971	17044	16841	18609	19753	17717	19180	17566	18326	15868
Mula	-1,46674	38,041031	16958	17645	16581	21123	16397	16144	18013	19087	16990	18493	16786	17656	17461
Torres de Cotillas	-1,3025362	38,006971	20078	21042	19818	24885	19801	19663	21272	22697	20483	21741	20094	21052	18136
Molina del Segura	-1,2206884	38,127483	19641	20659	19236	24288	19333	19132	20785	22267	19726	21116	19604	20526	17740
Molina del Segura	-1,2336707	38,071139	20305	21107	19932	24994	19897	19799	21236	22661	20578	21871	20238	21147	16562
Abanilla	-1,0655079	38,170041	20222	20965	19736	24813	19685	19486	21017	22366	20396	21735	20140	20960	16357
Fortuna	-1,1526819	38,161028	20019	20788	19755	24564	19529	19389	21004	22202	20420	21763	19831	20842	18035
Ojos	-1,3394287	38,113316	18264	19044	17922	22649	17921	17681	19403	20664	18365	19918	18319	19105	20152
Beniel	-0,9997837	38,034507	20913	21595	20446	25395	20338	20204	21634	23011	21145	22403	20648	21612	17121
Murcia	-1,2682702	37,898166	17770	18154	17356	21900	17082	16940	18588	19782	17875	19271	17629	18395	20591
Murcia	-0,9840042	37,977528	20127	20708	19671	24769	19517	19370	20910	22319	20387	21713	20097	20872	19020
Murcia	-1,1347189	37,940075	19148	19758	18765	23467	18510	18408	19974	21253	19380	20654	19006	19848	18376
Fitero	-1,8426437	42,046077	7315	7045	7410	12055	7765	7100	8805	8801	7906	9634	8277	8374	7523
Cascante	-1,7239555	42,034371	8627	8166	8707	13705	9015	8485	10243	10357	9392	11149	9623	9770	8404
Ablitas	-1,6447131	41,996446	10420	10436	10735	13357	11217	9787	12077	13235	11792	13204	11234	11590	8014
Murillo el fruto	-1,4871859	42,38498	8846	8612	8378	11610	8334	8353	10354	10558	9050	10460	9098	9423	7240
Miranda de Arga	-1,8087315	42,511252	8300	8120	8073	11513	8303	7720	9717	10141	8502	10125	8349	8988	6931
Falces	-1,7925482	42,409669	8349	8217	8330	11884	8457	7857	9894	10209	8906	10493	8359	9178	7716
Bargota	-2,2992201	42,477657	7812	7574	7519	10913	7638	7305	9076	9453	8130	9724	7845	8454	7798
Bardenas Reales	-1,5187546	42,295154	8591	8239	8173	11288	8301	8327	10424	10043	8939	10328	8945	9236	7954
Los Arcos	-2,1845206	42,539308	7075	7103	6924	10170	7104	6633	8538	8968	7378	8998	7280	7834	7131

Sesma	-2,126631	42,473409	7639	7424	7456	10947	7751	7151	9157	9430	8009	9581	7626	8379	7237
Cadreita	-1,6556731	42,26433	9543	8949	8718	12236	9032	8987	11181	10852	9905	11304	9491	10018	7244
Bardenas Reales	-1,7183027	42,207768	9402	9069	8996	12723	9171	8630	10922	11076	9850	11393	9127	10033	7246
Sartaguda	-2,0512344	42,361948	8805	8598	8662	12177	8812	8266	10321	10572	9241	10829	8733	9547	6674
Olite	-1,662579	42,423779	8651	8364	8164	11345	8295	8407	10351	10185	8963	10327	8988	9276	6528
Murillo el Cuende	-1,6153521	42,361474	8804	8275	8027	11402	8309	8277	10239	10115	9060	10439	9072	9275	7035
Corella	-1,8398436	42,115577	8285	7829	8454	13233	8753	8155	9885	9634	8904	10660	9229	9366	6379
Funes	-1,8066789	42,287885	9361	9183	9176	12698	9263	8769	10995	11281	9810	11428	9245	10110	6172
Lerin	-1,9763006	42,503595	8257	7989	7925	11254	8105	7598	9631	9914	8467	10003	8192	8849	6293
Los Palacios y Villafranca	-5,9390554	37,179127	21709	23375	21229	25186	21250	20947	22753	24134	22102	22988	20806	22407	16993
Las cabezas de San Juan	-5,884722	37,01556	21867	23560	21642	25365	21509	21256	23144	24284	22337	23378	21290	22694	17551
Lebrija	-6,1261602	36,976641	22399	24075	21908	25767	21922	21700	23205	24638	22772	23833	21630	23077	16160
Aznalcazar	-6,2733503	37,151795	21498	22752	20807	25040	20759	20210	21888	23495	21778	22922	20658	21982	16078
Isla Mayor	-6,1512787	37,098521	21652	23362	21467	25149	21402	20995	22800	24072	22298	23249	21021	22497	17349
La puebla del Rio	-6,1338321	37,226032	21607	23409	21366	25052	21321	20880	22686	24155	22110	23121	20933	22422	16764
La puebla del Rio II	-6,0465691	37,080174	21693	23247	21353	25228	21212	20696	22634	24072	22232	23175	21082	22420	16884
Ecija	-5,0770704	37,592934	18832	20744	19590	21920	18981	18319	20425	22132	19981	20705	18774	20036	14839
La Luisiana	-5,2281407	37,525293	19013	20879	18677	22619	18966	18365	20371	21787	19517	20615	18555	19942	14321
Carmona	-5,587615	37,400903	20769	22442	20440	24402	20521	20011	21678	23408	21352	22247	20184	21587	15593
Osuna	-5,1348377	37,25503	19388	21138	19135	22911	19182	18660	20595	21898	19837	20972	18898	20238	15003
La Rinconada	-5,924839	37,456832	21531	23498	21373	24633	21304	20823	22642	24003	22084	22936	20633	22315	16711
Sanlucar La Mayor	-6,2550749	37,42179	20515	22190	19984	24277	20171	19574	21267	22805	20797	22120	19806	21228	16128
Villanueva del Rio y Minas	-5,6840093	37,613036	20712	22476	20537	24260	20579	20107	21763	23483	21389	22206	20027	21594	16285
Lora del Rio	-5,5407037	37,660906	19592	21389	19229	23303	19476	18926	20632	22242	20196	21155	18846	20453	15889
Los Molares	-5,6729697	37,176152	21022	22790	20893	24596	20863	20398	22205	23638	21643	22642	20488	21925	15547
Guillena	-6,06419	37,514568	20960	22705	20879	24570	20748	20478	22253	23647	21605	22599	20466	21901	17550
Puebla Cazalla	-5,3509152	37,218131	19175	21028	18871	23118	19017	18608	20646	21952	19521	20856	18856	20150	16263
Alcala del Rio	-5,9641033	37,512529	21623	23403	21417	25053	21487	20970	22810	24217	22307	23149	21099	22503	16502
San Javier	-0,819705	37,791664	22994	23635	22193	27262	21981	22022	23546	25035	23062	24175	22882	23526	18431
Torre Pacheco	-0,8985888	37,773803	23243	23736	22666	27557	22424	22366	23701	25271	23487	24655	23001	23828	16515
San Javier	-0,8836862	37,848045	22627	23314	21880	26921	21765	21835	23270	24863	22668	23792	22518	23223	17803
Torre Pacheco	-0,9316281	37,823827	22076	22776	21558	26647	21510	21348	22783	24325	22256	23483	21909	22788	17435
Torre Pacheco	-0,9867861	37,74765	22873	23734	22220	27519	22236	22049	23612	25237	23058	24316	22703	23596	17124

Pedralba	-0,7175861	39,567014	16402	17365	16787	20694	16102	15680	17478	18908	17210	18487	16751	17442	15377
Liria	-0,627062	39,691055	16562	17626	17279	20471	16178	16040	17569	18838	17371	18561	16823	17575	13811
Benifayo	-0,4618662	39,280627	20995	20986	20537	24503	19446	19287	20856	22375	20733	21758	20067	21049	17141
Cheste	-0,7444395	39,518889	14995	15975	15280	19237	14557	14215	16040	17282	15505	16905	15331	15939	14095
Tabernes de Valldigna	-0,2380292	39,095261	20107	20039	19582	23915	18563	18478	20137	21731	19819	20976	19443	20254	18680
Villanueva de Castellon	-0,5242892	39,065567	20111	20109	19615	23885	18687	18385	20024	21663	19820	20937	19233	20224	16526
Sagunto	-0,2663216	39,647534	19996	19926	19460	23397	18376	18251	19942	21575	19636	20655	19136	20032	17992
Benavites	-0,2162186	39,730391	19222	19153	18630	22827	17813	17441	19190	20894	18691	19860	18568	19299	15340
Moncada	-0,3989602	39,587729	19595	19501	19015	23242	18078	17990	19500	21306	19141	20211	18878	19678	16163
Carcagente	-0,4461657	39,113604	20594	20420	20123	24221	18966	19027	20370	21882	20304	21348	19690	20632	15405
Carlet	-0,5459462	39,22642	17972	19178	18477	22762	17671	17411	19078	20538	19019	20241	18261	19146	16337
Luchente	-0,3600825	38,938508	15778	15558	15147	19496	14180	13952	15825	17109	15415	16657	15125	15840	12858
Requena	-1,2323883	39,504667	8420	9379	8623	11989	8442	8087	9752	10675	8752	10292	9081	9408	6847
Algemesi	-0,4353656	39,216442	21065	20915	20421	24714	19472	19398	20808	22441	20597	21625	20151	21055	16576
Campo Arcis	-1,1622154	39,433357	8991	10018	9200	12569	8966	8460	10203	11357	9429	10963	9563	9974	8411
Betera	-0,4685258	39,597708	18903	18829	18334	22453	17477	17196	18849	20568	18582	19695	18001	18990	15132
Picasent	-0,4976324	39,362484	19416	19402	18860	23168	17903	17714	19433	21034	19010	20104	18588	19512	15768
Montesa	-0,6383798	38,954502	15376	16291	15582	19925	15000	14774	16415	17752	16041	17389	15790	16394	15324
Jativa	-0,5497109	38,998803	19145	19057	18559	22960	17538	17552	19028	20565	18675	19731	18210	19184	15352
Villalonga	-0,2042579	38,892111	19426	19401	18783	23043	17622	17552	19395	20811	18863	20035	18690	19420	17148
Gandia	-0,2506841	38,964297	17973	18038	17522	21814	16559	16341	18197	19707	17697	18980	17456	18208	16252
Bolbaite	-0,6901658	39,069153	15202	16114	15553	19706	14804	14564	16349	17596	15875	17239	15638	16240	14060
Chulilla	-0,8322414	39,67678	14532	15707	15033	18815	14379	13931	15780	17034	15299	16654	14994	15651	12949
Almoacid de la Sierra	-1,3299642	41,452078	10781	10861	10796	13538	11418	10157	12400	13570	11830	13358	11567	11843	9393
Belchite	-0,7216155	41,350306	11470	11359	11603	14284	11911	10828	12935	14163	12616	14076	12144	12490	8981
Quinto	-0,5186373	41,388348	13699	13596	13737	14397	12672	12030	14268	15322	14206	15435	12987	13850	9761
Fabara	0,1540344	41,167877	13646	13269	13379	14656	12322	11823	14150	15241	14002	15362	13078	13721	9435
Epila	-1,2820466	41,583234	11388	11504	11825	14680	12244	10975	12988	14429	13195	14512	12113	12714	9369
Ejea de los Caballeros	-1,1961298	42,097715	9021	9264	9235	11796	9766	8600	10555	11998	10249	11651	9940	10189	7350
Sabada	-1,309387	42,267312	8823	8269	8134	11581	8428	8484	10525	10338	9026	10444	9110	9378	6618
Luna	-0,9359498	42,095761	8295	8422	8616	11247	9038	7837	9913	11108	9542	10915	9210	9468	6204
Santa Engracia	-1,3305062	41,921246	11053	11140	11351	13818	11532	10609	12446	13903	12298	13699	11788	12149	8541
Pastriz	-0,7461599	41,59514	11357	11180	11709	14227	12105	10780	13002	14287	12947	14309	12049	12541	9507

Zaragoza	-0,823819	41,71363	10275	10352	10639	13194	11190	9753	12092	13289	11884	13305	11137	11555	9114
Calatayud	-1,6583593	41,332167	7416	7511	7395	10386	7993	6888	8960	9919	8588	9991	8410	8496	7377
Borja	-1,5076903	41,855146	10372	10287	10381	13121	10803	9650	11758	13048	11427	12882	11166	11354	8278
Tarazona	-1,7458841	41,916177	7378	7142	7353	11845	7649	7071	8551	9104	7887	9608	8264	8350	7465
Caspe	-0,0710973	41,303968	14511	14001	14012	15325	12975	12663	14736	16061	14969	16175	13841	14479	9995
Osera de Ebro	-0,5363755	41,545081	12730	12583	12501	13608	11715	11058	13370	14724	13281	14589	12395	12960	9589
Daroca	-1,4247095	41,108073	5728	5975	5744	8224	6159	5410	7064	7950	6496	7924	6677	6668	6089
Zuera	-0,7511391	41,869389	9712	9793	9989	12550	10538	9380	11292	12632	11064	12486	10511	10904	9049
El Bayo	-1,2487734	42,175713	8697	8900	8973	11888	9488	8125	10194	11658	10119	11477	9511	9912	7044
Tauste	-1,1428386	42,00023	10463	10656	10845	13536	11319	9962	12108	13495	11876	13308	11177	11704	8384
Boquiñeni	-1,2496818	41,843217	10629	10873	11100	13754	11651	10362	12529	13976	12407	13880	11743	12082	9069
