# Supplementary material for: Agroclimatic Metrics for the Main Stone Fruit Producing Areas in Spain in Current and Future Climate Change Scenarios: Implications From an Adaptive Point of View
Source: Front Plant Sci. 2022 Jun 8;13:842628. doi: 10.3389/fpls.2022.842628 (PMC9213681; doi:10.3389/fpls.2022.842628)
Supplement: Supplementary file 6 [file Data_Sheet_6.PDF]

Supplementary Table 6. Mean accumulated GDHs (1st January - Beginning April) for 2025-2045, RCP 8.5 at each location.

The last column shows the heat accumulation for the current situation, for comparison purposes

M1: bcc-csm1-1-m; **M2:** BNU-ESM; **M3:** CanESM2; **M4:** CMCC-CM; **M5:** GFDL-ESM2G; **M6:** inmcm4

M7: IPSL-CM5A-LR; **M8:** MIROC-ESM; **M9:** MPI-ESM-LR; **M10:** MPI-ESM-MR; **M11:** MRI-CGCM3

Municipality	Longitude	Latitude	M1	M2	M3	M4	M5	M6	M7	M8	M9	M10	M11	MEAN	CURRENT
Campo de Mirra	-0,7729762	38,679366	11652	10307	11542	13994	10711	10320	11685	13083	10622	11420	11905	11567	9222
Villajoyosa	-0,2561866	38,527917	23205	22176	22925	26449	21315	21369	22948	24811	21563	22023	22306	22826	19022
Ondara	0,0065631	38,818581	22747	21306	22519	26106	20551	20655	22250	24054	21024	21542	21414	22197	17381
Denia Gata	0,082579	38,792724	21817	20521	21418	25163	19883	19775	21392	23187	20087	20559	20756	21323	16297
Pinoso	-1,060721	38,427413	12903	11572	12757	15495	11847	11641	12973	14300	11833	12693	12831	12804	10446
Monforte del Cid	-0,7303963	38,398862	20756	19576	20451	24244	19264	19422	20605	22370	19526	20290	20225	20612	15732
Crevillente	-0,7831581	38,240831	23205	22231	22773	26729	21736	21760	22983	24666	22108	22766	22485	23040	20193
Almoradi	-0,7745396	38,031431	23468	22527	22659	26921	21887	21938	23237	24825	22191	22803	22826	23207	19077
Callosa de Sarria	-0,1044988	38,650249	18950	17458	18578	22042	16971	16900	18540	20215	17233	17945	18166	18454	19540
Pilar de la Horadada	-0,8125284	37,868588	24461	23605	23773	27862	22960	22906	24156	25940	23267	23826	23838	24236	19235
Catral	-0,8055704	38,153124	23532	22537	22905	27107	22079	22098	23356	25003	22371	22974	22917	23353	17734
Altea	-0,0795078	38,603643	21443	19994	20890	24737	19251	19360	21063	22564	19752	20290	20491	20894	18704
Planes	-0,3529076	38,78476	14893	13345	14670	17857	13269	12971	14713	16339	13393	14086	14519	14551	12544
Villena	-0,8753684	38,595491	15580	14171	15330	18468	14326	14116	15632	17067	14418	15189	15285	15416	10401
Agost	-0,6498214	38,421512	19524	18394	19204	22944	18094	18014	19440	21194	18288	19039	19108	19386	14888
Almansa	-1,1075837	38,903228	8937	8065	9452	11864	8410	8235	9198	10844	8494	9191	9740	9311,8	8608
Ontur	-1,4957688	38,622866	14112	12717	13755	17012	12956	12911	14259	15537	12950	13754	13992	13996	9229
Caudete	-0,9798818	38,734665	13286	12047	12970	16037	12277	12073	13427	14831	12203	13064	13325	13231	10377
La Mojonera	-2,7043824	36,787318	23196	21412	22736	26155	21764	21975	23559	23823	22214	22776	23021	22966	20088
Almeria	-2,4024534	36,835404	27537	26151	26887	30099	26256	26166	27904	28325	26567	26980	27348	27293	22117
Nijar	-2,1580794	36,95057	24543	22998	24084	27573	23243	23173	24969	25375	23590	24108	24153	24346	17941
Tabernas	-2,3023755	37,091315	18708	16988	18532	21741	17748	17616	19335	19682	17914	18649	18569	18680	13428
Fiñana	-2,8388277	37,156718	10803	10136	11466	12487	10571	9873	11410	12715	10197	11096	11626	11125	9292
Cuevas de Almanzora	-1,7704017	37,389125	22687	20937	22630	25915	21451	21440	23015	23517	21876	22459	22574	22591	17179
Huercal-overa	-1,8842832	37,412428	20428	18757	20480	23584	19496	19224	20812	21408	19711	20273	20313	20408	14947
Cuevas de Almanzora	-1,800522	37,256757	26019	24426	25635	28926	24628	24502	26300	26724	25097	25660	25613	25775	18782
Adra	-2,9923491	36,746758	25292	24748	25785	27498	24325	23997	25752	27140	24744	25178	25040	25409	9714
Tijola	-2,457021	37,371918	15534	13576	15382	18466	14451	14337	16107	16271	14661	15493	15742	15456	21485

Totana	-1,5130934	37,732459	21144	20220	20719	24551	19882	19932	21107	22729	20096	20794	20669	21077	15888
Alhama	-1,4167602	37,7922	21272	20203	20630	24745	19968	19906	21305	22816	20096	20759	20677	21125	15937
Librilla	-1,3382889	37,899373	20133	19073	19876	23401	18781	18953	20090	21495	18983	19718	19678	20016	16941
Mazarron	-1,4009916	37,56215	23748	21986	22940	27147	21852	22174	23595	24567	22118	22685	23112	23266	19904
Mazarron	-1,3788416	37,614572	22754	21252	22175	26081	20831	21189	22719	23884	21387	21889	22302	22406	17834
Zalamea de la Serena	-5,6910276	38,678704	15431	15365	15239	18098	14700	14662	15684	18122	14468	15362	15237	15670	11553
Monterrubio de la Serena	-5,3836134	38,591582	14106	14035	13970	16833	13530	13474	14656	16650	13311	14219	14062	14440	10402
Don Benito	-5,9062469	38,930491	17806	17184	17613	20130	16610	16998	17584	19899	16361	17115	16871	17652	11831
Villagonzalo	-6,1858738	38,837247	16886	17149	16980	19013	16234	16547	16890	19715	16162	17072	15931	17144	12917
Jerez de los Caballeros	-6,7369024	38,281336	17202	17357	17074	20378	16553	16467	17122	19657	16757	17669	16737	17543	12068
Olivenza	-7,0578251	38,720921	17110	17594	17204	20375	16577	16377	17306	19787	16922	17817	16432	17591	12264
Villafranca de los Barros	-6,3485695	38,575591	16139	16364	16064	19201	15691	15449	16183	18844	15683	16544	15800	16542	11971
Merida	-6,3192869	38,845149	17002	17114	17041	19083	16304	16642	16933	19704	16200	17151	16102	17207	12588
Azuaga	-5,7077922	38,391445	13946	13950	13877	16641	13458	13275	14395	16545	13088	14038	14060	14298	8697
Puebla de alcocer	-5,0955855	39,074643	14629	13877	14638	16943	13675	13952	14690	16885	13561	14349	14463	14696	11005
Don Benito	-5,858992	38,984723	17825	17412	17589	20056	16750	16903	17675	20143	16399	17100	16847	17700	11755
Badajoz	-6,827838	38,877039	17454	17590	17429	19456	16728	16991	17323	20169	16588	17467	16345	17594	12939
Pueblonuevo del Guadiana	-6,7328012	38,912998	17467	17508	17625	19419	16676	16962	17350	20151	16605	17478	16419	17605	13133
Lobon	-6,6655535	38,860185	17339	17443	17629	19520	16684	17159	17365	20046	16749	17635	16372	17631	13035
Arroyo de San Servan	-6,4728164	38,858246	16758	16770	16951	18816	16058	16320	16762	19540	16010	16894	15995	16989	13290
Villar de Reina	-5,742601	39,102295	16326	15698	16314	18765	15458	15663	16448	18730	15149	15965	15841	16396	11883
Cartagena	-0,9508754	37,688833	23870	22143	23274	27538	22089	22269	23543	24699	22519	22969	23432	23486	17708
Murcia	-1,1227711	37,831265	21315	20219	20865	24733	19662	19940	21156	22590	19926	20638	20706	21068	17019
Fuente alamo	-1,1292626	37,748269	23972	22967	23175	27272	22412	22531	23663	25338	22629	23186	23179	23666	17111
Cartagena	-1,070786	37,676671	23992	23048	23466	27527	22514	22580	23793	25380	22906	23444	23263	23810	18270
Cartagena	-0,8037931	37,611152	23659	22051	23176	26999	21684	21921	23373	24627	22049	22544	23230	23210	18375
Fuente alamo	-1,2380371	37,699008	21833	20827	21333	25329	20351	20546	21849	23414	20670	21302	21370	21711	16850
Casatejada	-5,6781	39,867824	15801	15334	15963	18058	15041	15210	16091	18150	14753	15478	15210	15917	10709
Aldehuela del Jerte	-6,2302346	40,008316	16047	15865	16575	18082	15981	15941	16161	18149	15321	16364	15293	16344	11790
Moraleja	-6,6759606	40,046357	16144	16007	16387	18497	15770	15795	15951	19039	15172	16155	15340	16387	11406
Coria	-6,5458096	39,978094	16304	16101	16570	18214	15940	15924	16181	18357	15224	16378	15349	16413	11523
Madrigalejo	-5,5954391	39,135847	16262	15819	16301	18558	15331	15504	16287	18631	15079	15864	15734	16306	11419
Valdesalor	-6,4785825	39,377191	15859	15781	15889	18141	15280	15146	15828	18653	15060	16009	15332	16089	9862

Peraleda de la Mata	-5,4639595	39,861132	15117	14715	15254	17307	14445	14423	15184	17412	14069	14796	14750	15225	10370
Tejeda de tietar	-5,8600359	39,960042	14727	13210	15104	17122	13939	14463	14559	15508	13761	14598	14036	14639	10910
Casar de Palomero	-6,3056933	40,298554	11308	10883	11658	13606	11273	10993	11505	14150	10647	11778	11567	11761	8771
Madroñera	-5,7623097	39,464885	12162	11577	12430	14730	11775	11381	12438	14709	11303	12246	12912	12515	9024
Guadalupe	-5,3482094	39,387141	12623	12024	12615	14861	11876	11892	12793	14990	11563	12468	12787	12772	8835
Alcantara	-6,8981123	39,746563	16918	17013	17140	19114	16380	16512	16823	19817	16166	17057	16057	17182	11894
Jarandilla de la Vega	-5,6463392	40,101413	10816	9761	11425	13158	10639	10720	11107	12211	10348	11294	11291	11161	9619
Gargantilla	-5,9414268	40,239041	9234	8009	9444	11486	8806	8784	9414	10627	8626	9706	9851	9453,5	10237
Talayuela	-5,5642306	40,011198	14216	12865	14657	16776	13678	13896	14244	15094	13339	14257	13814	14258	11239
Valdastillas	-5,8687982	40,141215	7650	7035	8063	10008	7769	7646	8124	10248	7312	8384	9157	8308,6	10911
Cieza	-1,3097249	38,235442	16560	15293	16420	19826	15336	15297	16599	18038	15446	16300	16229	16486	16973
Ulea	-1,2578423	38,191392	19223	17893	18943	22732	17733	17896	19193	20650	17964	18716	18645	19053	16196
Cieza	-1,4963438	38,283888	18490	17382	18273	21927	17281	17443	18680	19992	17572	18309	18086	18494	15774
Calasparra	-1,6947638	38,253487	18172	17152	17806	21547	17056	16995	18335	19892	17269	18028	17815	18188	14071
Calasparra	-1,5850018	38,269499	18849	17670	18549	22168	17389	17767	18948	20259	17793	18583	18301	18752	15712
Caravaca	-1,980057	38,043911	11862	11134	11480	15911	10799	11067	12223	13590	10735	11638	12964	12128	7108
Cehegin	-1,6828994	38,110901	15763	14563	15652	18924	14716	14611	15895	17308	14855	15586	15537	15764	11849
Moratalla	-1,813186	38,196653	14318	13453	13775	19256	12994	13485	14528	16007	13019	13982	15066	14535	12201
Cehegin	-1,7798922	38,104477	13741	12966	13357	18505	12488	12890	13955	15430	12580	13481	14532	13993	12661
Moratalla	-2,0961142	38,1145	6814	6067	6593	9548	6229	5953	7269	8326	5961	6815	8307	7080,1	6230
Vall de Uxo	-0,2304536	39,795861	20406	18948	20153	23503	18511	18186	20052	21891	18742	19313	19149	19896	16062
Onda	-0,2444114	39,954016	18696	17057	19045	21772	16888	17197	18708	20761	17451	18147	18120	18531	15457
San Rafael del Rio	0,3675272	40,594077	18879	16994	18949	21467	16983	16944	18740	20934	17658	18329	18074	18541	12433
Benicarlo	0,4014538	40,411511	21026	19313	21249	23654	19031	19031	20818	23007	19693	20338	20111	20661	15350
Castellon	-0,1191495	39,989342	19352	17781	19815	22221	17618	17532	19392	21437	18376	18969	18630	19193	14403
Burriana	-0,1057138	39,887849	21618	20121	21383	24772	19672	19557	21230	23026	20047	20616	20358	21127	14492
Ribera de Cabanes	0,1464314	40,133934	19174	17563	19491	21861	17370	17244	19234	21259	17963	18690	18658	18955	15515
Nules	-0,1683946	39,877237	20975	19278	20621	24015	18755	18801	20488	22185	19091	19658	19726	20327	15342
Segorbe	-0,4830876	39,817295	15691	14030	15363	18748	13931	13778	15428	17102	14091	14728	15027	15265	12538
Baza	-2,7677154	37,564477	15464	13196	15175	18739	14450	14449	16169	16165	14815	15630	15856	15464	8746
Puebla de Don Fadrique	-2,3817176	37,876115	8668	7884	8421	12164	7959	8076	9196	10319	7651	8603	10085	9002,5	6059
Loja	-4,138128	37,1693	16268	15900	16475	18395	15330	15164	16555	18788	15431	16244	16064	16419	11528
Iznalloz	-3,5514591	37,416406	14481	13968	15461	16845	14464	13670	15471	17147	14260	15188	15213	15106	7813

Jerez del Marquesado	-3,1498644	37,190536	11026	10344	11653	12821	10942	10255	11756	13129	10517	11379	11871	11427	6034
Cadiar	-3,183988	36,923123	14267	13676	15211	16383	14100	13429	14976	16509	13858	14717	14919	14731	8834
Zafarraya	-4,1538389	36,990314	10757	10099	11075	12385	10204	9864	11250	12723	9925	10798	11458	10958	6981
Padul	-3,600317	37,018743	14965	14416	16047	17204	15024	14092	15817	17516	14716	15631	15543	15543	10682
Granada	-3,6385645	37,172054	15623	15147	16763	18346	15652	14654	16527	18347	15491	16367	16214	16284	9930
Almuñecar	-3,6790578	36,751942	21111	20627	21765	23217	20343	19886	21674	23016	20457	21105	21117	21302	19144
Gibraleon	-7,0278022	37,318328	23949	23796	23486	26745	23331	22472	23538	25693	23603	24142	22891	23968	17580
Lepe	-7,2430825	37,302685	23564	23245	23147	26213	22662	21897	23522	25284	23063	23670	22782	23550	18243
Gibraleon	-7,059841	37,412354	22397	22058	22094	25115	21762	20947	22239	24200	21983	22554	21608	22451	16505
Moguer	-6,7925285	37,14648	23279	23095	22897	26096	22511	21731	23165	25114	22773	23370	22440	23315	16529
Niebla	-6,7353478	37,347125	22822	22630	22290	25597	22011	21310	22456	24521	22232	22931	21814	22783	15475
Aroche	-6,9449915	37,958077	16910	17099	17019	20115	16437	16165	17020	19494	16463	17455	16852	17366	13062
La puebla de Guzman	-7,2483655	37,552176	20232	20111	20156	23125	19977	18882	20366	22436	20017	20683	19918	20537	14447
El Campillo	-6,5992719	37,660989	19761	19410	19361	22517	19119	18383	19545	21725	19275	20078	19430	19873	14634
La Palma del Condado	-6,5415566	37,366968	21365	21078	20894	24186	20515	19762	21100	23097	20701	21434	20560	21335	16323
Almonte	-6,4765444	37,148345	23580	23491	23189	26295	22857	21957	23379	25324	23094	23700	22609	23589	17632
Valfarta	-0,1478858	41,531503	12342	10750	12631	13625	11134	10590	13091	14711	11786	12843	11961	12315	7854
Zaidin	0,2890014	41,637169	12846	11324	13190	14066	11686	11099	13544	15089	12333	13399	12336	12810	8991
Alcolea de Cinca	0,0731411	41,74095	12212	10664	12792	13435	11418	10792	13104	14560	11824	12831	11958	12326	9264
Tanarite de Litera	0,3771357	41,780947	11725	10266	12072	13119	10982	10542	12797	14377	11574	12546	11798	11982	8032
Lanaja	-0,337846	41,786429	11357	9955	11947	12650	10597	9989	12130	14073	10887	11847	11168	11509	7542
Monzon	0,1273494	41,957687	10770	9257	10961	12147	9989	9369	11726	12992	10777	11776	10832	10963	7986
Barbastro	0,1126102	42,013471	10669	9069	11116	12035	9997	9401	11429	13019	10519	11473	10734	10860	7606
Sariñena	-0,1766614	41,771411	11888	10380	12338	13416	11201	10572	13032	14401	11744	12758	11872	12146	8429
Huesca	-0,3777068	42,105429	10281	8782	10450	11593	9664	9041	11205	12766	10128	11022	10663	10508	7354
Candasnos	0,094436	41,45994	13470	11965	13545	14767	12230	11782	14102	15686	12815	13845	13051	13387	8357
Grañen	-0,3560041	41,942469	10340	8951	10831	12095	9942	9097	11433	13026	10409	11306	10705	10740	7471
Huerto	-0,1365362	41,966019	10242	8733	10712	11942	9637	8996	11331	12880	10360	11319	10517	10606	7532
Gurrea de Gallego	-0,7311994	41,992829	9869	8346	10180	12599	10340	9572	11174	12586	10785	11535	11393	10762	7380
Alfantega	0,1477817	41,821958	10686	9119	11180	12335	10101	9509	11775	13157	10776	11707	10940	11026	8660
Fraga	0,3539314	41,495165	14067	12336	14528	15109	13027	12445	14703	16190	13386	14415	13632	13985	10630
Tardienta	-0,5075831	41,969367	11289	9622	11500	12321	10512	9825	12150	13738	10883	11780	11191	11346	7498
San Esteban de Litera	0,3042037	41,882938	10429	9045	10848	11637	9819	9242	11472	12925	10385	11283	10674	10705	7885

Belver de Cinca	0,2318291	41,742536	11764	10220	12392	12836	11057	10334	12975	14069	11457	12436	11597	11922	8268
Alberuela de Tubo	-0,2573084	41,883957	11687	10253	12079	13037	10771	10167	12545	14150	11339	12362	11520	11810	8239
Jumilla	-1,4232837	38,394834	15436	14005	15430	18534	14273	14335	15616	16833	14359	15228	15242	15390	12831
Yecla	-1,1859032	38,658948	13140	11764	13010	15866	12095	11901	13298	14576	12070	12892	13228	13076	9356
Yecla	-1,1125211	38,562731	14529	13202	14440	17467	13445	13309	14680	15980	13528	14352	14388	14484	10386
Jumilla	-1,2407841	38,392588	14719	13367	14620	17693	13527	13495	14958	16217	13631	14458	14493	14652	14005
Jumilla	-1,3242866	38,31972	17439	16267	17144	20772	16192	16118	17596	18999	16364	17165	17068	17375	14101
Aitona	0,4609093	41,486913	14226	12785	14998	15598	13340	12748	15244	16720	13895	14974	13673	14382	10472
Albesa	0,6705502	41,760356	12915	10714	12548	13431	11874	10912	12413	13964	11400	12437	11208	12165	7850
Alcarras	0,5506119	41,56508	13078	11322	13476	14619	11962	11628	14023	15419	12787	13762	12893	13179	9669
Alfarras	0,5780224	41,819488	11868	9840	11551	12461	10999	10068	11640	13172	10633	11595	10478	11300	7900
Algerri	0,6483717	41,801036	11801	9602	11385	12275	10653	9904	11507	12802	10472	11359	10415	11107	7835
Alguaire	0,5361346	41,742812	10728	9124	10866	11926	9908	9467	11695	13096	10631	11547	10889	10898	7300
Castellnou de Seana	0,9520619	41,65659	13105	10770	12523	13705	11650	10921	12596	14055	11497	12441	11391	12241	8101
Cervera	1,2967772	41,662217	9284	7288	8797	9815	8445	7610	9231	10455	8082	8856	8608	8770,1	5778
Gimenells i el Pla de la Font	0,3933398	41,658132	11932	10358	12135	13206	10986	10495	12880	14330	11651	12566	11700	12022	8217
Golmes	0,9248038	41,63641	13334	11092	12858	14137	12400	11366	12753	14520	11702	12619	11508	12572	7850
Raimat	0,4490319	41,683272	11198	9650	11759	12685	10546	9861	12408	13699	11030	12061	11204	11464	7949
Balaguer- Monasterio Avellanas	0,7613663	41,879114	10814	8835	10460	11558	10088	9006	10540	11996	9649	10535	9768	10295	6546
El Canos	1,2041447	41,689385	10165	8050	9770	10686	9226	8335	9963	11301	8916	9767	9250	9584,4	6424
El Poal	0,8777387	41,672786	13334	11091	12857	14136	12400	11365	12752	14519	11701	12619	11507	12571	7942
Sant Marti de Riucorb	1,0885432	41,572353	11417	9346	10857	12032	10603	9467	11025	12598	9990	10909	10161	10764	7173
Seros	0,4279758	41,463784	14206	12512	14498	15567	13067	12675	15243	16585	13951	15022	13806	14285	10702
Tarrega	1,1626814	41,666945	11203	9077	10712	11687	10072	9268	11108	12344	9802	10720	10003	10545	7631
Tornabous	1,0451011	41,68834	12296	10249	11979	12973	11402	10339	11924	13544	10841	11694	10624	11624	7694
Vallfogona de Balaguer	0,8293888	41,784868	12429	10261	11973	12983	11269	10311	12053	13408	10984	11872	10791	11667	8188
Vilanova de Segria	0,628392	41,714499	13424	11304	12986	14054	12213	11447	13059	14478	11983	12964	11743	12696	8126
Lorca	-1,6294551	37,601733	19059	17282	18483	22546	17355	17789	19159	19711	17713	18288	18877	18751	14349
Lorca	-1,6938893	37,50379	20170	18582	20203	23345	19165	18850	20688	21135	19473	20058	20052	20156	14445
Agoncillo	-2,2904337	42,468182	8333	6617	8243	11571	7995	7942	9762	9852	8557	9346	8926	8831,3	7321
Albelda de Iregua	-2,4718558	42,380733	7330	5942	7486	10379	7353	6912	8453	9007	7549	8293	8138	7894,9	6251
Asenjo	-2,1533164	42,340952	7847	6401	7911	11050	7690	7372	9279	9626	8055	8878	8299	8400,8	6115
Logroño	-2,5136369	42,43969	7829	6274	7764	11042	7620	7438	9157	9431	8168	8989	8399	8373,7	6630

Santa Engracia del Juvera	-2,2629377	42,368971	8105	6448	7998	11295	7918	7572	9397	9642	8360	9126	8591	8586,6	5469
Aldea Nueva de Ebro	-1,9048679	42,222598	9944	8034	9788	13502	9506	9227	11537	11409	10230	11036	10078	10390	7993
Alfaro	-1,7776916	42,152119	9103	6880	9445	14211	9538	8923	10216	10276	9781	10802	10781	9995,9	7539
Calahorra	-2,001826	42,334834	9733	7873	9476	13209	9427	9123	11250	11223	10013	10841	9911	10189	7768
Corvera (Cabreton)	-1,8924542	42,006904	8013	6047	8171	12429	8207	7648	9087	9362	8497	9471	9575	8773,4	7174
Igea	-1,9937535	42,05775	6714	5196	6879	10518	6928	6467	7648	8296	7111	7946	8332	7457,8	6635
Quel	-2,037178	42,252488	8735	7239	8815	12110	8677	8318	10262	10624	9085	9897	9270	9366,7	6982
Rincon de Soto	-1,8508464	42,251583	9943	8034	9788	13502	9506	9226	11536	11408	10230	11035	10077	10390	7998
Aguilas	-1,5921627	37,418665	24445	22982	23901	27496	22799	22796	24328	25559	22963	23447	23767	24044	20895
Lorca	-1,8177885	37,855634	13045	12165	12590	17314	11713	12132	13216	14602	11733	12620	13826	13178	10245
Lorca	-1,623984	37,4878	20546	18885	20047	24191	18837	18908	20383	21381	19141	19696	20483	20227	17680
Puerto Lumbreras	-1,7255508	37,590472	20118	18471	20050	23208	19113	18921	20693	21124	19402	19990	19979	20097	14926
Mula	-1,4294482	38,065871	19041	17855	18881	22590	17738	17804	19173	20651	17987	18711	18738	19015	15868
Mula	-1,46674	38,041031	18467	17313	18159	21711	17167	17130	18609	19979	17297	18082	17954	18352	17461
Torres de Cotillas	-1,3025362	38,006971	21884	20907	21261	25455	20486	20525	21853	23541	20818	21476	21295	21773	18136
Molina del Segura	-1,2206884	38,127483	21337	20513	20710	24857	20022	20101	21300	23092	20090	20780	20763	21233	17740
Molina del Segura	-1,2336707	38,071139	21947	21023	21361	25602	20671	20627	21885	23484	20879	21528	21312	21847	16562
Abanilla	-1,0655079	38,170041	21928	20916	21237	25450	20378	20438	21699	23295	20684	21303	21236	21687	16357
Fortuna	-1,1526819	38,161028	21611	20629	21323	25173	20266	20383	21685	23095	20538	21351	20879	21539	18035
Ojos	-1,3394287	38,113316	19965	18878	19498	23263	18578	18625	19906	21508	18693	19489	19505	19810	20152
Beniel	-0,9997837	38,034507	22571	21510	22015	26009	21010	21205	22352	23850	21374	22021	21780	22336	17121
Murcia	-1,2682702	37,898166	19251	18022	18968	22550	17902	17909	19159	20648	18060	18817	18756	19095	20591
Murcia	-0,9840042	37,977528	21809	20657	21265	25402	20291	20368	21626	23246	20633	21270	21234	21618	19020
Murcia	-1,1347189	37,940075	20713	19565	20381	24097	19264	19349	20614	22110	19571	20244	20193	20555	18376
Fitero	-1,8426437	42,046077	7988	5969	8278	12672	8216	7729	9061	9108	8458	9419	9577	8770,6	7523
Cascante	-1,7239555	42,034371	9399	7132	9616	14281	9582	9138	10462	10528	9912	10907	10890	10168	8404
Ablitas	-1,6447131	41,996446	11235	9665	11679	14089	11589	10615	12553	13944	12017	12794	12522	12064	8014
Murillo el fruto	-1,4871859	42,38498	9366	7710	9188	12315	8734	8944	11043	11126	9259	10045	10154	9807,6	7240
Miranda de Arga	-1,8087315	42,511252	8822	7192	8857	12148	8689	8331	10302	10461	8996	9808	9444	9368,3	6931
Falces	-1,7925482	42,409669	8956	7296	9122	12520	8739	8454	10346	10551	9259	10068	9362	9515,6	7716
Bargota	-2,2992201	42,477657	8333	6617	8243	11571	7995	7942	9762	9852	8557	9346	8926	8831,2	7798
Bardenas Reales	-1,5187546	42,295154	9112	7379	9021	11911	8760	8855	10987	10667	9095	9820	9953	9596,3	7954
Los Arcos	-2,1845206	42,539308	7589	6133	7692	10755	7411	7232	9027	9326	7797	8674	8357	8181,1	7131

Sesma	-2,126631	42,473409	8108	6498	8201	11547	8020	7744	9553	9800	8443	9181	8722	8710,6	7237
Cadreita	-1,6556731	42,26433	10073	8027	9606	13012	9456	9618	11970	11465	10000	10735	10490	10405	7244
Bardenas Reales	-1,7183027	42,207768	9938	8196	9857	13435	9473	9250	11618	11440	10217	11008	10288	10429	7246
Sartaguda	-2,0512344	42,361948	9383	7673	9495	12823	9152	8911	10802	10892	9678	10449	9734	9908,4	6674
Olite	-1,662579	42,423779	9168	7434	9035	11997	8828	8920	11038	10795	9058	9789	10010	9642,9	6528
Murillo el Cuende	-1,6153521	42,361474	9307	7386	8962	12064	8755	8827	10929	10728	9216	9893	10119	9653,3	7035
Corella	-1,8398436	42,115577	8983	6732	9378	13871	9317	8754	10097	9913	9439	10447	10527	9768,8	6379
Funes	-1,8066789	42,287885	9920	8277	9952	13411	9687	9391	11524	11610	10275	11078	10348	10498	6172
Lerin	-1,9763006	42,503595	8749	7122	8746	11868	8449	8255	10213	10268	8871	9685	9203	9220,8	6293
Los Palacios y Villafranca	-5,9390554	37,179127	23153	22960	22514	25688	22046	21851	23055	24962	22369	22928	21720	23022	16993
Las cabezas de San Juan	-5,884722	37,01556	23360	23046	22923	25822	22318	22134	23498	25221	22641	23250	22374	23326	17551
Lebrija	-6,1261602	36,976641	23889	23672	23204	26341	22656	22485	23640	25620	22970	23649	22642	23706	16160
Aznalcazar	-6,2733503	37,151795	22864	22499	22130	25556	21852	20977	22366	24390	22147	22752	21630	22651	16078
Isla Mayor	-6,1512787	37,098521	23095	22953	22744	25614	22100	21751	23266	24943	22541	23072	22015	23099	17349
La puebla del Rio	-6,1338321	37,226032	23097	22937	22613	25669	22132	21735	23085	25025	22414	22974	21989	23061	16764
La puebla del Rio II	-6,0465691	37,080174	23110	22864	22769	25768	22052	21551	23242	25016	22473	22991	22153	23090	16884
Ecija	-5,0770704	37,592934	20293	20275	20810	22556	19702	19282	20993	22993	19994	20526	19862	20662	14839
La Luisiana	-5,2281407	37,525293	20517	20254	19981	23098	19749	19210	20666	22586	19826	20523	19605	20547	14321
Carmona	-5,587615	37,400903	22215	21919	21699	24930	21246	20836	22177	24224	21607	22218	21190	22205	15593
Osuna	-5,1348377	37,25503	20896	20570	20539	23395	19923	19526	21012	22850	20136	20826	20026	20882	15003
La Rinconada	-5,924839	37,456832	23037	22952	22552	25231	22033	21786	23013	24892	22310	22796	21557	22924	16711
Sanlucar La Mayor	-6,2550749	37,42179	21875	21805	21268	24866	21138	20396	21424	23614	21330	22024	20835	21870	16128
Villanueva del Rio y Minas	-5,6840093	37,613036	22236	21973	21731	24815	21331	20953	22286	24223	21618	22168	20979	22210	16285
Lora del Rio	-5,5407037	37,660906	20991	20830	20566	23851	20203	19703	21002	23037	20391	21141	19845	21051	15889
Los Molares	-5,6729697	37,176152	22460	22298	22119	25152	21626	21245	22708	24504	21963	22481	21577	22558	15547
Guillena	-6,06419	37,514568	22478	22186	22206	25060	21616	21430	22675	24460	21854	22525	21470	22542	17550
Puebla Cazalla	-5,3509152	37,218131	20671	20432	20222	23533	19778	19482	20932	22918	19915	20743	19999	20784	16263
Alcala del Rio	-5,9641033	37,512529	23085	22933	22638	25564	22324	21809	23337	25087	22443	23047	22038	23119	16502
San Javier	-0,819705	37,791664	24599	23724	23803	27890	22853	22983	24312	26037	23195	23673	24032	24282	18431
Torre Pacheco	-0,8985888	37,773803	24839	23801	24204	28182	23272	23225	24580	26137	23586	24163	24108	24554	16515
San Javier	-0,8836862	37,848045	24293	23305	23454	27586	22558	22781	23965	25648	22918	23458	23606	23961	17803
Torre Pacheco	-0,9316281	37,823827	23753	22753	23069	27304	22254	22278	23525	25178	22462	23079	23021	23516	17435
Torre Pacheco	-0,9867861	37,74765	24546	23680	23734	28081	23093	22958	24304	26031	23317	23899	23783	24311	17124

Pedralba	-0,7175861	39,567014	17737	16986	18372	21568	16744	16721	18022	20046	17107	17909	17719	18085	15377
Liria	-0,627062	39,691055	17729	17175	18788	21363	16977	16942	17976	19958	17280	17998	17745	18175	13811
Benifayo	-0,4618662	39,280627	22399	20827	22109	25336	20175	20189	21777	23514	20692	21211	20868	21736	17141
Cheste	-0,7444395	39,518889	16221	15596	16897	20113	15281	15244	16477	18341	15573	16335	16370	16586	14095
Tabernes de Valldigna	-0,2380292	39,095261	21485	19912	21252	24799	19421	19439	21084	22821	19811	20342	20278	20968	18680
Villanueva de Castellon	-0,5242892	39,065567	21480	20015	21196	24735	19409	19269	20872	22759	19872	20404	20083	20918	16526
Sagunto	-0,2663216	39,647534	21256	19787	21128	24320	19320	19190	20810	22617	19634	20058	20023	20740	17992
Benavites	-0,2162186	39,730391	20471	19021	20272	23720	18646	18314	20056	21859	18783	19390	19377	19992	15340
Moncada	-0,3989602	39,587729	20958	19417	20647	24118	18916	18788	20462	22321	19215	19688	19610	20376	16163
Carcagente	-0,4461657	39,113604	21863	20386	21710	25042	19694	19900	21307	22970	20240	20760	20438	21301	15405
Carlet	-0,5459462	39,22642	19321	18771	20097	23706	18395	18506	19653	21741	18850	19642	19224	19810	16337
Luchente	-0,3600825	38,938508	17120	15325	16891	20335	14971	15026	16656	18295	15444	16017	16082	16560	12858
Requena	-1,2323883	39,504667	9402	8593	10002	12543	8971	8824	9750	11399	9111	9858	10165	9874,4	6847
Algemesi	-0,4353656	39,216442	22337	20875	21997	25510	20198	20349	21806	23502	20557	21091	20905	21739	16576
Campo Arcis	-1,1622154	39,433357	10000	9288	10582	13181	9457	9151	10316	12131	9697	10438	10640	10444	8411
Betera	-0,4685258	39,597708	20176	18671	19946	23368	18288	18077	19769	21615	18634	19143	18800	19681	15132
Picasent	-0,4976324	39,362484	20800	19242	20544	24062	18692	18696	20280	22176	19123	19580	19498	20245	15768
Montesa	-0,6383798	38,954502	16740	15813	17129	20777	15700	15813	16817	18967	15986	16813	16777	17030	15324
Jativa	-0,5497109	38,998803	20527	18941	20158	23757	18245	18560	19967	21628	18736	19232	19007	19887	15352
Villalonga	-0,2042579	38,892111	20806	19267	20515	23962	18491	18691	20384	22098	18910	19453	19493	20188	17148
Gandia	-0,2506841	38,964297	19369	17913	19245	22693	17327	17336	19002	20858	17761	18385	18284	18925	16252
Bolbaite	-0,6901658	39,069153	16467	15652	17237	20570	15494	15543	16605	18775	15869	16678	16705	16872	14060
Chulilla	-0,8322414	39,67678	15847	15166	16596	19651	14976	14910	16112	18084	15398	16144	15976	16260	12949
Almoacid de la Sierra	-1,3299642	41,452078	11603	10148	11848	14213	11869	11078	12732	14375	12296	12984	12903	12368	9393
Belchite	-0,7216155	41,350306	12290	10536	12663	15067	12321	11750	13462	14862	12748	13663	13379	12977	8981
Quinto	-0,5186373	41,388348	14070	12973	14802	15306	13257	12749	15347	16843	13696	14707	13654	14309	9761
Fabara	0,1540344	41,167877	14234	12679	14582	15554	12900	12580	15232	16596	13552	14687	13702	14209	9435
Epila	-1,2820466	41,583234	12379	10808	12875	15556	12796	11930	13494	15329	13544	14147	13282	13285	9369
Ejea de los Caballeros	-1,1961298	42,097715	9826	8436	10208	12384	10176	9313	11096	12628	10541	11290	11260	10651	7350
Sabada	-1,309387	42,267312	9323	7441	9038	12298	8854	9037	11281	10877	9192	9996	10126	9769,4	6618
Luna	-0,9359498	42,095761	9170	7657	9621	11856	9499	8604	10342	11690	9882	10537	10453	9937,4	6204
Santa Engracia	-1,3305062	41,921246	11791	10370	12320	14571	11782	11404	12938	14547	12478	13237	13154	12599	8541
Pastriz	-0,7461599	41,59514	12156	10504	12737	15123	12692	11612	13596	14956	13139	13925	13273	13065	9507

Zaragoza	-0,823819	41,71363	11057	9669	11749	13967	11794	10636	12643	14012	12179	12792	12397	12081	9114
Calatayud	-1,6583593	41,332167	8118	6763	8433	11007	8419	7666	9170	10611	9022	9574	9645	8947,9	7377
Borja	-1,5076903	41,855146	11214	9525	11376	13862	11165	10461	12267	13714	11704	12543	12507	11849	8278
Tarazona	-1,7458841	41,916177	7988	6097	8289	12394	8094	7673	8740	9438	8377	9328	9541	8723,5	7465
Caspe	-0,0710973	41,303968	14886	13454	15179	16202	13650	13291	15799	17458	14444	15461	14379	14928	9995
Osera de Ebro	-0,5363755	41,545081	13228	12069	13593	14488	12337	11670	14404	15985	12993	13980	12985	13430	9589
Daroca	-1,4247095	41,108073	6391	5199	6629	8671	6645	6043	7109	8442	7035	7599	7907	7061	6089
Zuera	-0,7511391	41,869389	10539	8970	10918	13204	10911	10129	11799	13154	11326	12109	11794	11350	9049
El Bayo	-1,2487734	42,175713	9579	8175	10020	12539	9948	8977	10655	12380	10460	11052	10855	10422	7044
Tauste	-1,1428386	42,00023	11388	9837	11960	14222	11848	10871	12619	14183	12289	12921	12440	12234	8384
Boquiñeni	-1,2496818	41,843217	11518	10096	12119	14586	12154	11227	13074	14676	12728	13447	13104	12612	9069
