# Supplementary material for: Agroclimatic Metrics for the Main Stone Fruit Producing Areas in Spain in Current and Future Climate Change Scenarios: Implications From an Adaptive Point of View
Source: Front Plant Sci. 2022 Jun 8;13:842628. doi: 10.3389/fpls.2022.842628 (PMC9213681; doi:10.3389/fpls.2022.842628)
Supplement: Supplementary file 7 [file Data_Sheet_7.PDF]

Supplementary Table 7. Mean accumulated GDHs (1st January - Beginning April) for 2045-2065, RCP 4.5 at each location.

The last column shows the heat accumulation for the current situation, for comparison purposes

M1: bcc-csm1-1-m; **M2:** BNU-ESM; **M3:** CanESM2; **M4:** CMCC-CM; **M5:** GFDL-ESM2G; **M6:** inmcm4

M7: IPSL-CM5A-LR; **M8:** MIROC-ESM; **M9:** MPI-ESM-LR; **M10:** MPI-ESM-MR; **M11:** MRI-CGCM3

Municipality	Longitude	Latitude	M1	M2	M3	M4	M5	M6	M7	M8	M9	M10	M11	MEAN	CURRENT
Campo de Mirra	-0,7729762	38,679366	11251	10979	11227	14710	10365	10582	12287	13553	10779	11344	11454	11685	9222
Villajoyosa	-0,2561866	38,527917	23175	22630	22726	27010	21105	21616	23392	25519	22192	22269	21945	23053	19022
Ondara	0,0065631	38,818581	22774	21606	22306	26667	20410	20819	22637	24589	21582	21944	21277	22419	17381
Denia Gata	0,082579	38,792724	21792	20863	21181	25681	19730	20078	21858	23782	20715	20872	20519	21552	16297
Pinoso	-1,060721	38,427413	12487	12183	12421	16205	11456	11927	13535	14724	12078	12616	12476	12919	10446
Monforte del Cid	-0,7303963	38,398862	20275	20174	20160	24975	19109	19647	21172	22796	19866	20419	19948	20776	15732
Crevillente	-0,7831581	38,240831	22796	22842	22576	27534	21774	22039	23617	25163	22401	22960	22270	23270	20193
Almoradi	-0,7745396	38,031431	23048	23061	22505	27662	21919	22243	23702	25348	22550	23102	22554	23427	19077
Callosa de Sarria	-0,1044988	38,650249	18810	17924	18292	22604	16727	17185	18983	20812	17778	18045	17841	18636	19540
Pilar de la Horadada	-0,8125284	37,868588	24018	24163	23546	28583	22969	23164	24772	26477	23685	24146	23483	24455	19235
Catral	-0,8055704	38,153124	23106	23207	22734	27930	22196	22423	23957	25560	22702	23306	22684	23619	17734
Altea	-0,0795078	38,603643	21371	20415	20633	25304	19098	19624	21405	23244	20351	20511	20208	21106	18704
Planes	-0,3529076	38,78476	14653	13894	14306	18439	12961	13314	15170	16931	13784	14080	14153	14699	12544
Villena	-0,8753684	38,595491	15085	14914	15009	19201	14062	14360	16214	17515	14610	15193	14948	15555	10401
Agost	-0,6498214	38,421512	19127	19043	18974	23665	17940	18284	20076	21722	18675	19176	18846	19593	14888
Almansa	-1,1075837	38,903228	8883	8820	9274	12548	8071	8446	9676	11266	8688	9139	9359	9470	8608
Ontur	-1,4957688	38,622866	13511	13458	13404	17799	12662	13195	14831	15940	13002	13675	13667	14104	9229
Caudete	-0,9798818	38,734665	12777	12798	12627	16743	11990	12342	13976	15247	12387	12953	12944	13344	10377
La Mojonera	-2,7043824	36,787318	22660	22030	22549	26985	21603	22025	23920	24266	22384	22748	22632	23073	20088
Almeria	-2,4024534	36,835404	27165	26614	26800	30867	26235	26241	28203	28878	26795	27101	26919	27438	22117
Nijar	-2,1580794	36,95057	24130	23585	23934	28287	23138	23148	25376	25861	23832	24194	23885	24488	17941
Tabernas	-2,3023755	37,091315	18105	17586	18309	22298	17446	17525	19863	20028	18191	18519	18189	18733	13428
Fiñana	-2,8388277	37,156718	10536	10889	11259	13043	9836	9891	11870	13059	10515	10706	10893	11136	9292
Cuevas de Almanzora	-1,7704017	37,389125	22265	21498	22536	26481	21253	21433	23528	23956	22199	22492	22114	22705	17179
Huercal-overa	-1,8842832	37,412428	19933	19295	20320	24233	19201	19219	21372	21710	19965	20243	19868	20487	14947
Cuevas de Almanzora	-1,800522	37,256757	25622	24910	25513	29578	24527	24522	26700	27259	25336	25708	25246	25902	18782
Adra	-2,9923491	36,746758	25333	25234	25807	28062	24002	23925	26154	27621	25151	25263	24535	25554	9714
Tijola	-2,457021	37,371918	14795	14384	15100	19098	14055	14312	16688	16574	14811	15174	15270	15478	21485

Totana	-1,5130934	37,732459	20680	20796	20509	25253	19737	20188	21635	23147	20272	20890	20344	21223	15888
Alhama	-1,4167602	37,7922	20792	20906	20412	25542	19902	20163	21819	23354	20267	20920	20418	21318	15937
Librilla	-1,3382889	37,899373	19786	19698	19549	24259	18563	19223	20683	21942	19256	19860	19276	20190	16941
Mazarron	-1,4009916	37,56215	23445	22435	22665	27695	21810	22419	24003	25057	22649	22868	22706	23432	19904
Mazarron	-1,3788416	37,614572	22440	21718	21962	26668	20890	21439	23155	24320	21841	22042	21970	22586	17834
Zalamea de la Serena	-5,6910276	38,678704	14896	17037	15138	19289	14396	14701	16527	18712	14440	15450	14852	15949	11553
Monterrubio de la Serena	-5,3836134	38,591582	13637	15478	13781	17864	13252	13535	15412	17295	13306	14286	13713	14687	10402
Don Benito	-5,9062469	38,930491	17493	18925	17648	21088	16319	17062	18635	20656	16275	17558	16566	18020	11831
Villagonzalo	-6,1858738	38,837247	16553	18661	17008	19909	15943	16525	17738	20392	16188	17462	15721	17464	12917
Jerez de los Caballeros	-6,7369024	38,281336	16664	18796	16918	21407	16212	16565	17934	20143	16632	17719	16333	17757	12068
Olivenza	-7,0578251	38,720921	16777	18842	17024	21358	16379	16401	17974	20380	17050	18045	16083	17847	12264
Villafranca de los Barros	-6,3485695	38,575591	15577	17861	15895	20306	15397	15587	16965	19421	15662	16698	15483	16805	11971
Merida	-6,3192869	38,845149	16716	18526	17088	20036	15997	16649	17746	20322	16160	17466	15859	17506	12588
Azuaga	-5,7077922	38,391445	13391	15455	13729	17782	13131	13472	15197	17026	13075	14131	13639	14548	8697
Puebla de alcocer	-5,0955855	39,074643	14208	15635	14608	17973	13301	14082	15540	17627	13444	14637	14069	15011	11005
Don Benito	-5,858992	38,984723	17444	19016	17503	21063	16414	16938	18646	20831	16304	17414	16660	18021	11755
Badajoz	-6,827838	38,877039	17215	19015	17398	20395	16372	16945	18101	20700	16464	17801	16175	17871	12939
Pueblonuevo del Guadiana	-6,7328012	38,912998	17165	18850	17581	20384	16299	17064	18172	20690	16578	17802	16173	17887	13133
Lobon	-6,6655535	38,860185	17048	18922	17652	20462	16376	17183	18104	20601	16722	18030	16102	17927	13035
Arroyo de San Servan	-6,4728164	38,858246	16487	18302	16903	19845	15727	16369	17441	20088	15970	17227	15757	17283	13290
Villar de Reina	-5,742601	39,102295	15973	17445	16319	19751	15056	15659	17255	19586	15036	16318	15578	16725	11883
Cartagena	-0,9508754	37,688833	23586	22646	23079	28052	22098	22639	24045	25310	23058	23160	23085	23705	17708
Murcia	-1,1227711	37,831265	20973	20769	20647	25474	19545	20230	21670	23104	20221	20822	20430	21262	17019
Fuente alamo	-1,1292626	37,748269	23521	23621	22895	28057	22432	22769	24260	25876	22901	23463	22886	23880	17111
Cartagena	-1,070786	37,676671	23630	23645	23300	28383	22539	22844	24315	26007	23222	23748	22984	24056	18270
Cartagena	-0,8037931	37,611152	23483	22419	22944	27544	21645	22321	23760	25188	22535	22677	22808	23393	18375
Fuente alamo	-1,2380371	37,699008	21431	21466	21116	26108	20270	20780	22358	23852	20985	21587	21006	21905	16850
Casatejada	-5,6781	39,867824	15359	17328	15970	18992	14613	15291	17014	19084	14645	15922	14936	16287	10709
Aldehuela del Jerte	-6,2302346	40,008316	15757	17706	16685	18788	15181	16021	16978	19163	15074	16571	14943	16624	11790
Moraleja	-6,6759606	40,046357	15816	17798	16347	19257	15072	15732	16662	19950	14970	16520	15067	16654	11406
Coria	-6,5458096	39,978094	15984	17852	16603	18941	15198	15912	16986	19415	15042	16605	14982	16684	11523
Madrigalejo	-5,5954391	39,135847	15803	17586	16247	19652	14973	15599	17279	19453	14972	16179	15477	16657	11419
Valdesalor	-6,4785825	39,377191	15393	17345	15780	19275	14837	15288	16545	19265	14952	16249	15093	16366	9862

Peraleda de la Mata	-5,4639595	39,861132	14726	16576	15211	18295	13990	14577	16109	18318	13954	15183	14455	15581	10370
Tejeda de tietar	-5,8600359	39,960042	14526	15281	15324	17949	13138	14666	15580	16742	13445	14836	13733	15020	10910
Casar de Palomero	-6,3056933	40,298554	10784	12664	11680	14364	10544	11114	12043	15032	10432	11829	11329	11983	8771
Madroñera	-5,7623097	39,464885	11570	13436	12353	15891	11203	11656	13210	15524	11136	12366	12472	12801	9024
Guadalupe	-5,3482094	39,387141	12093	13768	12511	15889	11369	12056	13523	15714	11388	12642	12461	13038	8835
Alcantara	-6,8981123	39,746563	16632	18496	17115	20035	16009	16464	17515	20467	16029	17392	15928	17462	11894
Jarandilla de la Vega	-5,6463392	40,101413	10334	11781	11507	14018	9724	10919	11957	13319	10021	11360	10957	11445	9619
Gargantilla	-5,9414268	40,239041	8666	9636	9370	12405	8242	8939	10086	11353	8378	9585	9603	9660,2	10237
Talayuela	-5,5642306	40,011198	13970	15139	14906	17561	12732	14088	15294	16501	12898	14408	13466	14633	11239
Valdastillas	-5,8687982	40,141215	7078	8606	8059	10826	7027	7771	8649	10821	7187	8184	8825	8457,7	10911
Cieza	-1,3097249	38,235442	16124	15843	16079	20547	15036	15529	17243	18434	15681	16269	15876	16606	16973
Ulea	-1,2578423	38,191392	18804	18578	18649	23450	17524	18157	19776	21100	18213	18838	18387	19225	16196
Cieza	-1,4963438	38,283888	18051	18038	17990	22795	17096	17674	19351	20407	17694	18358	17768	18657	15774
Calasparra	-1,6947638	38,253487	17572	17829	17532	22433	16880	17245	18944	20270	17407	18026	17497	18330	14071
Calasparra	-1,5850018	38,269499	18347	18380	18266	23046	17336	17948	19481	20602	17954	18593	18082	18912	15712
Caravaca	-1,980057	38,043911	11541	11747	11257	16700	10473	11280	12747	13999	10934	11420	12345	12222	7108
Cehegin	-1,6828994	38,110901	15303	15291	15318	19691	14473	14823	16489	17766	15009	15597	15198	15905	11849
Moratalla	-1,813186	38,196653	13973	14043	13528	20063	12695	13721	15059	16498	13367	13920	14530	14672	12201
Cehegin	-1,7798922	38,104477	13431	13515	13122	19257	12214	13143	14469	15927	12864	13380	14035	14123	12661
Moratalla	-2,0961142	38,1145	6513	6650	6463	10239	5760	6239	7657	8720	6114	6529	7603	7135,2	6230
Vall de Uxo	-0,2304536	39,795861	20312	19377	19970	24003	18188	18519	20326	22411	19094	19671	18920	20072	16062
Onda	-0,2444114	39,954016	18551	17712	18832	22280	16756	17504	19240	20947	17605	18384	17938	18705	15457
San Rafael del Rio	0,3675272	40,594077	18563	17611	18628	22013	16853	17340	19291	21087	17763	18766	17782	18700	12433
Benicarlo	0,4014538	40,411511	20742	19948	20943	24230	18805	19366	21368	23189	19834	20751	19756	20812	15350
Castellon	-0,1191495	39,989342	19263	18416	19548	22646	17365	17850	19928	21588	18597	19353	18356	19355	14403
Burriana	-0,1057138	39,887849	21549	20635	21191	25265	19403	19843	21589	23556	20491	21012	20143	21334	14492
Ribera de Cabanes	0,1464314	40,133934	19044	18272	19337	22402	17136	17619	19780	21548	18329	19010	18226	19155	15515
Nules	-0,1683946	39,877237	20914	19734	20382	24500	18426	19076	20815	22634	19427	19995	19471	20488	15342
Segorbe	-0,4830876	39,817295	15565	14474	15042	19185	13538	14061	15727	17468	14332	14838	14828	15369	12538
Baza	-2,7677154	37,564477	14591	13964	14818	19532	14153	14408	16861	16385	14918	15432	15398	15497	8746
Puebla de Don Fadrique	-2,3817176	37,876115	8352	8578	8253	12910	7623	8328	9606	10717	7796	8310	9485	9087	6059
Loja	-4,138128	37,1693	15920	16922	16219	19321	14941	15129	17072	19216	15600	16058	15537	16540	11528
Iznalloz	-3,5514591	37,416406	14119	14845	15274	17704	13672	13558	16126	17511	14456	14833	14525	15148	7813

Jerez del Marquesado	-3,1498644	37,190536	10713	11190	11437	13448	10196	10261	12280	13448	10726	11014	11195	11446	6034
Cadiar	-3,183988	36,923123	14007	14519	14997	17022	13366	13354	15554	16847	14107	14320	14202	14754	8834
Zafarraya	-4,1538389	36,990314	10359	11089	10815	13070	9630	9892	11659	13152	10064	10374	10806	10992	6981
Padul	-3,600317	37,018743	14611	15297	15865	18022	14180	14002	16471	17902	14915	15226	14858	15577	10682
Granada	-3,6385645	37,172054	15390	16022	16618	19279	14868	14637	17237	18728	15726	16078	15554	16376	9930
Almuñecar	-3,6790578	36,751942	20981	21115	21654	23878	19812	19786	22013	23401	20797	20858	20574	21352	19144
Gibraleon	-7,0278022	37,318328	23704	24786	23420	27452	23029	22420	24002	26160	23415	24203	22572	24106	17580
Lepe	-7,2430825	37,302685	23387	24075	23061	27020	22358	21858	23945	25763	23023	23628	22409	23684	18243
Gibraleon	-7,059841	37,412354	22003	22979	21963	25854	21401	20894	22761	24646	21863	22522	21225	22556	16505
Moguer	-6,7925285	37,14648	22991	23979	22763	26888	22244	21737	23727	25502	22695	23400	22095	23456	16529
Niebla	-6,7353478	37,347125	22495	23623	22127	26377	21747	21211	23014	25067	22121	22879	21376	22913	15475
Aroche	-6,9449915	37,958077	16311	18474	16841	21215	16058	16395	17775	19969	16365	17417	16405	17566	13062
La puebla de Guzman	-7,2483655	37,552176	19948	21052	19948	24033	19490	18960	20936	22935	19781	20546	19422	20641	14447
El Campillo	-6,5992719	37,660989	19252	20526	19173	23490	18706	18388	20100	22190	18970	19755	18911	19951	14634
La Palma del Condado	-6,5415566	37,366968	20950	22091	20714	24974	20134	19801	21639	23574	20565	21292	20150	21444	16323
Almonte	-6,4765444	37,148345	23313	24363	23085	27043	22574	21892	23811	25790	23029	23712	22264	23716	17632
Valfarta	-0,1478858	41,531503	12722	11458	12649	14241	11174	10892	13196	14830	12286	12996	11511	12541	7854
Zaidin	0,2890014	41,637169	13269	12034	13164	14735	11750	11410	13644	15222	12841	13616	11887	13052	8991
Alcolea de Cinca	0,0731411	41,74095	12587	11482	12768	14133	11382	11079	13193	14739	12303	12962	11525	12559	9264
Tanarite de Litera	0,3771357	41,780947	12064	11112	12146	13705	10898	10899	12915	14370	11984	12857	11355	12210	8032
Lanaja	-0,337846	41,786429	11644	10767	11885	13310	10511	10234	12219	14193	11289	12016	10724	11708	7542
Monzon	0,1273494	41,957687	11018	9940	10948	12789	10068	9675	11737	13195	11120	11856	10344	11154	7986
Barbastro	0,1126102	42,013471	10791	9931	11011	12694	10016	9734	11481	13270	10845	11642	10223	11058	7606
Sariñena	-0,1766614	41,771411	12310	11087	12327	13943	11237	10781	13000	14542	12185	12939	11455	12346	8429
Huesca	-0,3777068	42,105429	10481	9629	10436	12203	9613	9321	11305	12818	10434	11293	10166	10700	7354
Candasnos	0,094436	41,45994	13901	12667	13503	15388	12258	12040	14147	15833	13304	14009	12610	13605	8357
Grañen	-0,3560041	41,942469	10567	9681	10815	12726	9867	9406	11547	13178	10753	11532	10207	10934	7471
Huerto	-0,1365362	41,966019	10371	9532	10756	12625	9716	9335	11356	13107	10673	11534	10029	10821	7532
Gurrea de Gallego	-0,7311994	41,992829	9602	9361	10193	13245	10106	10033	11620	12642	10504	11906	10705	10902	7380
Alfantega	0,1477817	41,821958	10941	9978	11169	13043	10135	9886	11862	13413	11167	11978	10446	11274	8660
Fraga	0,3539314	41,495165	14471	13201	14452	15719	12991	12703	14829	16335	13967	14528	13233	14221	10630
Tardienta	-0,5075831	41,969367	11508	10452	11507	12943	10633	10029	12244	13865	11264	11979	10649	11552	7498
San Esteban de Litera	0,3042037	41,882938	10646	9880	10853	12244	9717	9595	11580	13016	10665	11533	10228	10905	7885

Belver de Cinca	0,2318291	41,742536	12246	11073	12397	13391	11027	10511	13044	14189	11892	12653	11199	12148	8268
Alberuela de Tubo	-0,2573084	41,883957	12031	10950	12098	13700	10840	10405	12604	14247	11779	12541	11119	12029	8239
Jumilla	-1,4232837	38,394834	15009	14655	15091	19317	13971	14594	16209	17262	14589	15235	14942	15534	12831
Yecla	-1,1859032	38,658948	12580	12446	12738	16658	11758	12158	13847	15014	12201	12822	12910	13194	9356
Yecla	-1,1125211	38,562731	14099	13884	14100	18236	13119	13536	15265	16398	13684	14330	14047	14609	10386
Jumilla	-1,2407841	38,392588	14285	14041	14207	18509	13261	13745	15539	16638	13851	14425	14167	14788	14005
Jumilla	-1,3242866	38,31972	16933	16924	16816	21662	16008	16403	18206	19446	16592	17185	16788	17542	14101
Aitona	0,4609093	41,486913	14889	13528	14994	16163	13349	12937	15262	16834	14486	15237	13365	14640	10472
Albesa	0,6705502	41,760356	12917	11455	12448	13956	11782	11193	12563	14225	11929	12905	10820	12381	7850
Alcarras	0,5506119	41,56508	13624	12076	13477	15316	11985	11923	14138	15564	13333	14106	12468	13455	9669
Alfarras	0,5780224	41,819488	11862	10629	11467	13077	10969	10381	11690	13410	10973	11896	10185	11503	7900
Algerri	0,6483717	41,801036	11768	10370	11335	12812	10642	10227	11540	13031	10839	11740	10054	11305	7835
Alguaire	0,5361346	41,742812	11002	9932	10890	12513	9887	9793	11775	13184	10975	11736	10482	11106	7300
Castellnou de Seana	0,9520619	41,65659	13142	11613	12493	14210	11634	11190	12666	14321	11958	12848	11034	12464	8101
Cervera	1,2967772	41,662217	9177	8044	8731	10364	8320	7756	9331	10720	8294	9053	8146	8903,3	5778
Gimenells i el Pla de la Font	0,3933398	41,658132	12325	11068	12186	13879	11078	10821	12940	14505	12196	12899	11255	12286	8217
Golmes	0,9248038	41,63641	13430	11934	12785	14732	12212	11639	12923	14801	12222	13189	11129	12818	7850
Raimat	0,4490319	41,683272	11600	10472	11760	13297	10569	10093	12490	13835	11526	12275	10759	11698	7949
Balaguer- Monasterio Avellanas	0,7613663	41,879114	10770	9552	10364	12141	10010	9305	10608	12279	9968	10859	9347	10473	6546
El Canos	1,2041447	41,689385	10089	8841	9732	11270	9171	8553	10095	11634	9178	10064	8787	9765	6424
El Poal	0,8777387	41,672786	13429	11933	12784	14731	12211	11638	12923	14800	12221	13189	11129	12817	7942
Sant Marti de Riucorb	1,0885432	41,572353	11432	10129	10710	12590	10469	9676	11113	12982	10388	11246	9759	10954	7173
Seros	0,4279758	41,463784	14603	13192	14581	16314	13133	12930	15416	16847	14469	15227	13351	14551	10702
Tarrega	1,1626814	41,666945	11129	9851	10642	12235	10054	9509	11216	12676	10158	10994	9635	10736	7631
Tornabous	1,0451011	41,68834	12380	11017	11874	13537	11316	10542	12111	13814	11237	12172	10253	11841	7694
Vallfogona de Balaguer	0,8293888	41,784868	12509	11034	11932	13463	11219	10611	12162	13651	11381	12280	10440	11880	8188
Vilanova de Segria	0,628392	41,714499	13468	12092	12913	14446	12189	11812	13195	14712	12498	13311	11421	12914	8126
Lorca	-1,6294551	37,601733	18659	17704	18156	23047	17263	17967	19634	20027	18107	18343	18526	18858	14349
Lorca	-1,6938893	37,50379	19735	19082	20058	23875	18906	18800	21204	21489	19849	20047	19680	20248	14445
Agoncillo	-2,2904337	42,468182	8208	7600	8343	11860	7763	8338	9835	10303	8445	9554	8618	8987,8	7321
Albelda de Iregua	-2,4718558	42,380733	7224	6885	7550	10761	6995	7333	8606	9468	7446	8435	7856	8050,8	6251
Asenjo	-2,1533164	42,340952	7646	7391	8008	11304	7439	7888	9429	10077	7978	9060	8062	8571,2	6115
Logroño	-2,5136369	42,43969	7649	7279	7828	11305	7388	7857	9359	9804	8080	9176	8183	8537,1	6630

Santa Engracia del Juvera	-2,2629377	42,368971	7953	7455	8094	11617	7578	8053	9511	10111	8171	9278	8352	8743	5469
Aldea Nueva de Ebro	-1,9048679	42,222598	9828	9151	9812	13794	9278	9756	11657	11983	9940	11157	9883	10567	7993
Alfaro	-1,7776916	42,152119	8787	8383	9539	14856	9052	9434	10614	10836	9593	10826	10349	10206	7539
Calahorra	-2,001826	42,334834	9572	8959	9575	13510	9117	9620	11460	11731	9811	11035	9636	10366	7768
Corvera (Cabreton)	-1,8924542	42,006904	7726	7312	8250	13057	7971	7983	9432	9803	8343	9484	9011	8942,8	7174
Igea	-1,9937535	42,05775	6432	6247	6898	11167	6607	6748	7899	8624	6979	8008	7876	7589,6	6635
Quel	-2,037178	42,252488	8618	8324	8879	12434	8328	8828	10416	11124	8864	10072	9043	9539,1	6982
Rincon de Soto	-1,8508464	42,251583	9827	9150	9811	13794	9277	9756	11657	11983	9940	11156	9883	10567	7998
Aguilas	-1,5921627	37,418665	24161	23418	23694	28079	22756	23101	24720	26163	23430	23648	23314	24226	20895
Lorca	-1,8177885	37,855634	12723	12717	12340	18022	11368	12320	13716	15061	12016	12457	13277	13274	10245
Lorca	-1,623984	37,4878	20262	19245	19783	24756	18700	19213	20837	21795	19654	19793	20091	20375	17680
Puerto Lumbreras	-1,7255508	37,590472	19706	18999	19900	23837	18885	18881	21138	21504	19674	19963	19649	20194	14926
Mula	-1,4294482	38,065871	18631	18442	18568	23327	17496	18058	19779	21098	18217	18796	18424	19167	15868
Mula	-1,46674	38,041031	18069	17948	17898	22466	16951	17333	19137	20436	17470	18086	17619	18492	17461
Torres de Cotillas	-1,3025362	38,006971	21289	21660	21070	26389	20511	20734	22488	23989	20980	21675	20979	21979	18136
Molina del Segura	-1,2206884	38,127483	20797	21258	20488	25666	19992	20309	21911	23621	20253	20980	20473	21432	17740
Molina del Segura	-1,2336707	38,071139	21496	21661	21177	26469	20596	20932	22394	24002	21071	21718	21087	22055	16562
Abanilla	-1,0655079	38,170041	21467	21507	21041	26230	20414	20682	22218	23840	20958	21468	20986	21892	16357
Fortuna	-1,1526819	38,161028	21216	21283	21060	26001	20224	20617	22272	23606	20911	21496	20677	21760	18035
Ojos	-1,3394287	38,113316	19388	19547	19192	24004	18450	18919	20548	21965	18915	19571	19193	19972	20152
Beniel	-0,9997837	38,034507	22124	22077	21778	26811	20990	21432	22889	24416	21635	22255	21489	22536	17121
Murcia	-1,2682702	37,898166	18859	18556	18688	23205	17734	18162	19718	21116	18355	18929	18454	19252	20591
Murcia	-0,9840042	37,977528	21313	21238	21023	26178	20258	20577	22152	23701	20971	21594	20895	21809	19020
Murcia	-1,1347189	37,940075	20359	20200	20096	24985	19160	19695	21189	22636	19864	20460	19828	20770	18376
Fitero	-1,8426437	42,046077	7633	7229	8371	13297	7887	8057	9416	9550	8280	9499	9106	8938,6	7523
Cascante	-1,7239555	42,034371	9043	8486	9711	15054	9230	9629	10900	11124	9709	11005	10472	10397	8404
Ablitas	-1,6447131	41,996446	10973	10651	11732	14655	11370	11007	13107	14008	11830	13132	11889	12214	8014
Murillo el fruto	-1,4871859	42,38498	9283	8673	9338	12718	8572	9352	11179	11552	9276	10217	9711	9988,2	7240
Miranda de Arga	-1,8087315	42,511252	8604	8307	8945	12497	8355	8779	10434	10921	8826	9985	9174	9529,9	6931
Falces	-1,7925482	42,409669	8769	8339	9232	12826	8506	8954	10599	11084	9155	10251	9104	9710,9	7716
Bargota	-2,2992201	42,477657	8208	7600	8343	11859	7763	8337	9835	10302	8445	9554	8617	8987,7	7798
Bardenas Reales	-1,5187546	42,295154	9109	8385	9144	12374	8576	9239	11204	11063	9113	9982	9509	9790,7	7954
Los Arcos	-2,1845206	42,539308	7401	7128	7750	11150	7257	7602	9186	9807	7789	8911	8023	8364	7131

Sesma	-2,126631	42,473409	8005	7532	8302	11885	7775	8157	9768	10318	8351	9430	8423	8904,1	7237
Cadreita	-1,6556731	42,26433	10073	9165	9684	13323	9308	10056	12099	11904	10124	10983	10088	10619	7244
Bardenas Reales	-1,7183027	42,207768	9819	9216	9941	13727	9214	9862	11718	11941	10062	11193	9989	10607	7246
Sartaguda	-2,0512344	42,361948	9236	8780	9569	13118	8826	9427	11039	11435	9500	10592	9563	10098	6674
Olite	-1,662579	42,423779	9179	8499	9112	12406	8571	9304	11199	11221	9149	9956	9553	9831,8	6528
Murillo el Cuende	-1,6153521	42,361474	9304	8381	9018	12460	8636	9211	11133	11141	9230	10110	9690	9846,8	7035
Corella	-1,8398436	42,115577	8672	8253	9460	14540	8840	9217	10476	10426	9213	10531	10040	9969,8	6379
Funes	-1,8066789	42,287885	9767	9387	10047	13749	9406	9948	11781	12145	10014	11267	10062	10688	6172
Lerin	-1,9763006	42,503595	8609	8181	8840	12178	8188	8709	10323	10702	8767	9821	8915	9384,9	6293
Los Palacios y Villafranca	-5,9390554	37,179127	22859	23916	22332	26562	21998	21618	23675	25488	22319	23015	21394	23198	16993
Las cabezas de San Juan	-5,884722	37,01556	23038	24160	22778	26798	22118	22028	24089	25643	22669	23299	21938	23505	17551
Lebrija	-6,1261602	36,976641	23571	24705	23113	27151	22557	22393	24254	26032	23061	23758	22290	23899	16160
Aznalcazar	-6,2733503	37,151795	22660	23364	22037	26323	21613	20909	22904	24935	22062	22871	21292	22816	16078
Isla Mayor	-6,1512787	37,098521	22866	23825	22692	26603	21997	21642	23799	25404	22562	23149	21699	23294	17349
La puebla del Rio	-6,1338321	37,226032	22758	24046	22517	26511	21941	21564	23622	25491	22415	22992	21591	23223	16764
La puebla del Rio II	-6,0465691	37,080174	22888	23757	22637	26712	21939	21448	23705	25456	22545	23056	21751	23263	16884
Ecija	-5,0770704	37,592934	20082	21137	20741	23547	19529	19085	21460	23514	20233	20546	19451	20848	14839
La Luisiana	-5,2281407	37,525293	20086	21465	19830	24031	19536	19107	21269	23145	19796	20538	19234	20731	14321
Carmona	-5,587615	37,400903	21846	23023	21580	25806	21089	20783	22674	24770	21585	22300	20891	22395	15593
Osuna	-5,1348377	37,25503	20532	21712	20364	24368	19675	19491	21477	23282	20119	20694	19649	21033	15003
La Rinconada	-5,924839	37,456832	22745	24101	22464	26068	21982	21519	23620	25396	22326	22934	21202	23123	16711
Sanlucar La Mayor	-6,2550749	37,42179	21509	22834	21130	25595	20829	20336	22013	24123	21163	21989	20406	21993	16128
Villanueva del Rio y Minas	-5,6840093	37,613036	21909	23133	21666	25740	21197	20753	22707	24754	21613	22274	20625	22397	16285
Lora del Rio	-5,5407037	37,660906	20721	22035	20446	24756	20026	19644	21572	23561	20425	21156	19492	21258	15889
Los Molares	-5,6729697	37,176152	22150	23399	22003	26083	21431	21135	23247	25027	21896	22516	21209	22736	15547
Guillena	-6,06419	37,514568	22109	23344	22095	26086	21418	21192	23197	25009	21924	22567	21148	22735	17550
Puebla Cazalla	-5,3509152	37,218131	20311	21675	20149	24566	19569	19447	21449	23326	19836	20607	19673	20964	16263
Alcala del Rio	-5,9641033	37,512529	22763	24023	22569	26502	22160	21596	23790	25572	22482	23222	21786	23315	16502
San Javier	-0,819705	37,791664	24246	24214	23699	28674	22839	23298	24798	26711	23583	23988	23660	24519	18431
Torre Pacheco	-0,8985888	37,773803	24478	24370	24064	28990	23295	23577	25092	26748	24010	24578	23820	24820	16515
San Javier	-0,8836862	37,848045	23835	23870	23199	28387	22549	23097	24498	26268	23225	23759	23268	24178	17803
Torre Pacheco	-0,9316281	37,823827	23286	23283	22861	28091	22281	22563	24016	25718	22777	23374	22721	23725	17435
Torre Pacheco	-0,9867861	37,74765	24126	24344	23522	28886	23090	23212	24920	26592	23616	24265	23462	24549	17124

Pedralba	-0,7175861	39,567014	17672	17626	18203	22187	16516	16798	18523	20444	17533	18079	17488	18279	15377
Liria	-0,627062	39,691055	17837	17989	18686	21988	16614	17094	18555	20420	17742	18204	17458	18417	13811
Benifayo	-0,4618662	39,280627	22392	21260	21892	25855	19985	20412	22245	23948	21124	21589	20741	21949	17141
Cheste	-0,7444395	39,518889	16249	16205	16631	20763	15023	15336	17112	18836	16015	16455	16001	16784	14095
Tabernes de Valldigna	-0,2380292	39,095261	21451	20335	20956	25193	19140	19619	21497	23365	20254	20680	20144	21149	18680
Villanueva de Castellon	-0,5242892	39,065567	21460	20411	20982	25215	19160	19512	21322	23180	20251	20744	20007	21113	16526
Sagunto	-0,2663216	39,647534	21281	20234	20886	24736	18993	19351	21166	23105	19982	20396	19796	20902	17992
Benavites	-0,2162186	39,730391	20522	19368	19988	24215	18299	18578	20412	22419	19157	19698	19130	20162	15340
Moncada	-0,3989602	39,587729	20953	19823	20389	24584	18594	19095	20872	22831	19575	20076	19520	20574	16163
Carcagente	-0,4461657	39,113604	21896	20691	21437	25498	19392	20127	21725	23393	20694	21095	20450	21491	15405
Carlet	-0,5459462	39,22642	19380	19490	19983	24340	18170	18578	20226	22222	19381	19884	18991	20059	16337
Luchente	-0,3600825	38,938508	17117	15662	16494	20836	14660	15188	17078	18769	15826	16192	15885	16701	12858
Requena	-1,2323883	39,504667	9237	9519	9811	13191	8713	9104	10229	11804	9195	9838	9862	10046	6847
Algemesi	-0,4353656	39,216442	22357	21305	21734	25952	19980	20481	22121	23957	21016	21502	20842	21932	16576
Campo Arcis	-1,1622154	39,433357	9883	10156	10416	13810	9212	9456	10869	12589	9815	10403	10381	10635	8411
Betera	-0,4685258	39,597708	20133	19109	19660	23740	17983	18320	20089	22036	18973	19430	18670	19831	15132
Picasent	-0,4976324	39,362484	20781	19650	20249	24546	18408	18875	20716	22654	19377	19895	19335	20408	15768
Montesa	-0,6383798	38,954502	16752	16633	17024	21331	15508	15901	17342	19462	16403	16968	16486	17255	15324
Jativa	-0,5497109	38,998803	20513	19308	19853	24220	18037	18734	20391	22161	19076	19580	18933	20073	15352
Villalonga	-0,2042579	38,892111	20810	19643	20172	24413	18233	18784	20756	22564	19332	19722	19352	20344	17148
Gandia	-0,2506841	38,964297	19317	18318	18960	23154	17049	17568	19518	21303	18151	18627	18144	19101	16252
Bolbaite	-0,6901658	39,069153	16548	16433	17105	21185	15229	15637	17214	19251	16272	16784	16431	17099	14060
Chulilla	-0,8322414	39,67678	15759	15934	16426	20279	14724	15057	16718	18511	15659	16291	15687	16459	12949
Almoacid de la Sierra	-1,3299642	41,452078	11436	11168	11854	14811	11540	11390	13186	14286	11972	13279	12246	12470	9393
Belchite	-0,7216155	41,350306	12185	11562	12665	15615	12042	12186	13974	15011	12654	13892	12747	13139	8981
Quinto	-0,5186373	41,388348	14728	13688	14933	15791	13227	12839	15357	16792	14250	14849	13229	14517	9761
Fabara	0,1540344	41,167877	14707	13336	14621	16154	13061	12731	15228	16718	14174	14822	13269	14438	9435
Epila	-1,2820466	41,583234	12144	11864	12864	16071	12376	12317	14033	15247	13186	14574	12675	13395	9369
Ejea de los Caballeros	-1,1961298	42,097715	9522	9447	10213	13042	10001	9735	11519	12734	10315	11646	10566	10795	7350
Sabada	-1,309387	42,267312	9338	8496	9134	12720	8682	9515	11443	11397	9247	10165	9734	9988,4	6618
Luna	-0,9359498	42,095761	8820	8641	9621	12524	9262	9069	10759	11830	9623	10959	9789	10082	6204
Santa Engracia	-1,3305062	41,921246	11657	11307	12392	15069	11674	11901	13405	14640	12362	13498	12452	12760	8541
Pastriz	-0,7461599	41,59514	12114	11506	12794	15650	12360	12226	14092	15151	12977	14351	12584	13255	9507

Zaragoza	-0,823819	41,71363	10975	10650	11772	14529	11387	11124	13140	14095	11908	13214	11727	12229	9114
Calatayud	-1,6583593	41,332167	7988	7570	8440	11532	8201	7990	9586	10624	8762	9831	9007	9048,3	7377
Borja	-1,5076903	41,855146	11023	10444	11414	14506	11016	10939	12780	13778	11514	12753	11823	11999	8278
Tarazona	-1,7458841	41,916177	7745	7281	8286	13068	7702	8084	9010	9793	8224	9384	9120	8881,7	7465
Caspe	-0,0710973	41,303968	15611	14174	15218	16687	13536	13582	15907	17578	14986	15722	13968	15179	9995
Osera de Ebro	-0,5363755	41,545081	13676	12743	13657	14908	12258	11974	14359	16000	13381	14185	12633	13616	9589
Daroca	-1,4247095	41,108073	6119	6008	6549	9201	6359	6327	7504	8481	6871	7786	7363	7142,6	6089
Zuera	-0,7511391	41,869389	10216	10027	10906	13839	10723	10595	12267	13319	11058	12476	11177	11509	9049
El Bayo	-1,2487734	42,175713	9282	9103	10069	13137	9742	9365	11137	12427	10246	11438	10177	10557	7044
Tauste	-1,1428386	42,00023	11203	10979	11864	14831	11639	11231	13158	14316	11931	13346	11732	12385	8384
Boquiñeni	-1,2496818	41,843217	11316	11146	12185	15099	11873	11722	13628	14730	12463	13819	12373	12759	9069
