# Supplementary material for: Agroclimatic Metrics for the Main Stone Fruit Producing Areas in Spain in Current and Future Climate Change Scenarios: Implications From an Adaptive Point of View
Source: Front Plant Sci. 2022 Jun 8;13:842628. doi: 10.3389/fpls.2022.842628 (PMC9213681; doi:10.3389/fpls.2022.842628)
Supplement: Supplementary file 8 [file Data_Sheet_8.PDF]

Supplementary Table 8. Mean accumulated GDHs (1st January - Beginning April) for 2045-2065, RCP 8.5 at each location.
The last column shows the heat accumulation for the current situation, for comparison purposes

M1: bcc-csm1-1-m; **M2:** BNU-ESM; **M3:** CanESM2; **M4:** CMCC-CM; **M5:** GFDL-ESM2G; **M6:** inmcm4
M7: IPSL-CM5A-LR; **M8:** MIROC-ESM; **M9:** MPI-ESM-LR; **M10:** MPI-ESM-MR; **M11:** MRI-CGCM3

Municipality	Longitude	Latitude	M1	M2	M3	M4	M5	M6	M7	M8	M9	M10	M11	MEAN	CURRENT
Campo de Mirra	-0,7729762	38,679366	13451	13244	12973	16447	11439	12621	13650	15920	13231	13226	11990	13472	9222
Villajoyosa	-0,2561866	38,527917	25476	25326	24523	29202	22380	23642	25648	27807	24717	24053	23071	25077	19022
Ondara	0,0065631	38,818581	24810	24316	24053	28697	21783	22673	24916	26882	24020	23574	22246	24361	17381
Denia Gata	0,082579	38,792724	23901	23570	22927	27882	21005	22095	24036	25899	23190	22567	21539	23510	16297
Pinoso	-1,060721	38,427413	14795	14575	14248	18125	12620	14034	15040	17110	14614	14576	13086	14802	10446
Monforte del Cid	-0,7303963	38,398862	22579	22725	22134	26935	20374	21516	23049	25119	22480	22280	20813	22728	15732
Crevillente	-0,7831581	38,240831	25015	25229	24504	29307	22839	23748	25556	27145	24935	24617	23142	25094	20193
Almoradi	-0,7745396	38,031431	25421	25641	24545	29627	23004	24089	25858	27360	25118	24765	23490	25356	19077
Callosa de Sarria	-0,1044988	38,650249	21078	20614	20104	24732	18034	19305	21005	23030	20357	19850	18695	20619	19540
Pilar de la Horadada	-0,8125284	37,868588	26344	26654	25578	30514	24028	24954	26812	28594	26222	25792	24366	26351	19235
Catral	-0,8055704	38,153124	25373	25648	24761	29790	23195	24176	25962	27571	25257	24899	23574	25473	17734
Altea	-0,0795078	38,603643	23556	23086	22452	27392	20385	21692	23615	25223	22823	22169	21267	23060	18704
Planes	-0,3529076	38,78476	16982	16573	16117	20542	14195	15566	16953	19251	16285	15929	14932	16666	12544
Villena	-0,8753684	38,595491	17383	17298	16860	21024	15191	16383	17849	19712	17173	17078	15552	17409	10401
Agost	-0,6498214	38,421512	21516	21586	20889	25646	19250	20308	21972	23946	21323	21087	19607	21557	14888
Almansa	-1,1075837	38,903228	10760	10822	10801	14094	9248	10426	10825	13541	10821	10961	9711	11092	8608
Ontur	-1,4957688	38,622866	15851	15851	15328	19682	13710	15248	16371	18281	15594	15507	14159	15962	9229
Caudete	-0,9798818	38,734665	15128	15139	14518	18610	13110	14445	15470	17627	14985	14900	13502	15221	10377
La Mojonera	-2,7043824	36,787318	25126	24581	24547	28809	22675	23988	25915	26203	25095	24690	23512	25013	20088
Almeria	-2,4024534	36,835404	29462	29067	28658	32660	27110	28123	30144	30701	29278	28825	27927	29269	22117
Nijar	-2,1580794	36,95057	26452	26046	25826	30147	24209	25075	27363	27628	26383	25956	24825	26355	17941
Tabernas	-2,3023755	37,091315	20534	20080	20211	24105	18494	19671	21587	22058	20717	20505	18971	20630	13428
Fiñana	-2,8388277	37,156718	12598	12846	12843	14401	11169	12083	13011	15045	12816	12748	11442	12818	9292
Cuevas de Almanzora	-1,7704017	37,389125	24735	23975	24388	28329	22483	23366	25490	25800	24777	24435	23036	24619	17179
Huercal-overa	-1,8842832	37,412428	22227	21716	22048	25914	20317	21086	23027	23601	22371	22118	20615	22276	14947
Cuevas de Almanzora	-1,800522	37,256757	27997	27405	27380	31450	25630	26449	28699	29000	27877	27567	26115	27779	18782
Adra	-2,9923491	36,746758	27290	27320	27376	29739	25413	26020	27930	29258	27551	27096	25679	27334	9714
Tijola	-2,457021	37,371918	17261	16807	16993	20818	15147	16482	18177	18663	17384	17253	15775	17342	21485

Totana	-1,5130934	37,732459	22876	23227	22426	27039	20919	22032	23451	25211	22809	22613	21172	23070	15888
Alhama	-1,4167602	37,7922	23062	23307	22422	27389	21016	22022	23764	25365	22924	22659	21231	23196	15937
Librilla	-1,3382889	37,899373	22028	22095	21545	26198	19736	21094	22560	24281	21905	21621	20216	22116	16941
Mazarron	-1,4009916	37,56215	25721	25130	24599	29743	22906	24392	26188	27052	25020	24554	23905	25383	19904
Mazarron	-1,3788416	37,614572	24626	24284	23744	28635	21947	23479	25165	26218	24231	23695	23031	24460	17834
Zalamea de la Serena	-5,6910276	38,678704	16980	18525	17033	20839	15343	16978	17667	20345	17039	17422	15622	17618	11553
Monterrubio de la Serena	-5,3836134	38,591582	15563	16950	15625	19455	14079	15713	16579	18897	15744	16150	14358	16283	10402
Don Benito	-5,9062469	38,930491	19285	20063	19057	22802	17353	18917	19771	22053	18602	19263	17460	19511	11831
Villagonzalo	-6,1858738	38,837247	18422	20146	18501	21390	17049	18419	18934	21703	18274	19176	16665	18971	12917
Jerez de los Caballeros	-6,7369024	38,281336	18681	20479	18739	22888	17305	18486	19078	21769	19071	19565	17354	19401	12068
Olivenza	-7,0578251	38,720921	18761	20543	18857	22957	17351	18442	19323	21899	19283	19821	17126	19488	12264
Villafranca de los Barros	-6,3485695	38,575591	17656	19484	17771	21832	16342	17650	18100	21037	17995	18560	16451	18443	11971
Merida	-6,3192869	38,845149	18604	20020	18592	21569	17155	18432	18972	21705	18339	19180	16814	19035	12588
Azuaga	-5,7077922	38,391445	15462	16943	15634	19206	14027	15610	16193	18902	15567	16062	14168	16161	8697
Puebla de alcocer	-5,0955855	39,074643	16209	16815	16150	19531	14421	16013	16586	19220	15737	16357	14781	16529	11005
Don Benito	-5,858992	38,984723	19372	20375	19025	22836	17478	18922	19869	22353	18696	19251	17435	19601	11755
Badajoz	-6,827838	38,877039	19086	20486	19049	21989	17611	18754	19446	22198	18730	19643	17164	19469	12939
Pueblonuevo del Guadiana	-6,7328012	38,912998	18989	20455	19150	21954	17523	18797	19355	22094	18748	19592	17125	19435	13133
Lobon	-6,6655535	38,860185	18825	20373	19191	21959	17576	18979	19286	22032	18880	19708	17053	19442	13035
Arroyo de San Servan	-6,4728164	38,858246	18348	19822	18574	21362	16941	18300	18716	21540	18248	19010	16680	18867	13290
Villar de Reina	-5,742601	39,102295	18016	18645	17865	21390	16247	17682	18430	21127	17441	18118	16389	18305	11883
Cartagena	-0,9508754	37,688833	25793	25109	24885	30080	23046	24481	26029	27088	25394	24696	24264	25533	17708
Murcia	-1,1227711	37,831265	23309	23302	22622	27434	20744	22112	23683	25230	22904	22577	21317	23203	17019
Fuente alamo	-1,1292626	37,748269	25833	26123	24988	29928	23461	24540	26350	27950	25520	25125	23880	25791	17111
Cartagena	-1,070786	37,676671	25947	26084	25303	30218	23633	24649	26390	27931	25842	25359	23962	25938	18270
Cartagena	-0,8037931	37,611152	25780	25030	24751	29690	22779	24305	25850	27185	25030	24354	24015	25343	18375
Fuente alamo	-1,2380371	37,699008	23726	23991	23143	28002	21475	22740	24392	26071	23623	23307	21962	23857	16850
Casatejada	-5,6781	39,867824	17438	18280	17462	20695	15845	17280	18061	20488	16957	17642	15771	17811	10709
Aldehuela del Jerte	-6,2302346	40,008316	17801	18573	18053	20322	17212	17899	18073	20791	17287	18427	15931	18215	11790
Moraleja	-6,6759606	40,046357	17964	18940	17903	20836	16936	17686	17881	21719	17380	18443	16045	18339	11406
Coria	-6,5458096	39,978094	17994	18914	18121	20492	17117	17818	18119	21114	17285	18526	15960	18314	11523
Madrigalejo	-5,5954391	39,135847	17688	18719	17710	21276	16060	17522	18271	20886	17268	17941	16249	18145	11419
Valdesalor	-6,4785825	39,377191	17473	18906	17518	20781	16204	17273	17699	20924	17238	18082	16007	18010	9862

Peraleda de la Mata	-5,4639595	39,861132	16779	17612	16719	19949	15228	16571	17206	19876	16269	16903	15273	17126	10370
Tejeda de tietar	-5,8600359	39,960042	16592	15837	16416	19484	14976	16367	16466	18297	15759	16600	14528	16484	10910
Casar de Palomero	-6,3056933	40,298554	13012	13795	13322	15715	12328	13032	13170	17086	12709	13779	11771	13611	8771
Madroñera	-5,7623097	39,464885	13848	14693	14185	17233	12490	13677	14136	17598	13583	14307	12896	14422	9024
Guadalupe	-5,3482094	39,387141	14211	15152	14280	17418	12588	14139	14600	17730	13894	14550	12847	14673	8835
Alcantara	-6,8981123	39,746563	18503	20011	18699	21426	17270	18270	18775	21840	18154	19111	16818	18989	11894
Jarandilla de la Vega	-5,6463392	40,101413	12406	12283	12815	15197	11537	12561	12541	14967	12244	13124	11305	12816	9619
Gargantilla	-5,9414268	40,239041	10692	10502	10898	13467	9685	10626	10695	13432	10519	11456	9643	11056	10237
Talayuela	-5,5642306	40,011198	16024	15431	15944	19170	14633	15886	16072	17945	15389	16159	14178	16075	11239
Valdastillas	-5,8687982	40,141215	9048	9653	9491	11778	8497	9410	9263	12905	9222	9951	8798	9819,5	10911
Cieza	-1,3097249	38,235442	18480	18388	18015	22515	16298	17607	18984	20864	18362	18248	16609	18579	16973
Ulea	-1,2578423	38,191392	21154	21015	20619	25433	18735	20110	21661	23277	20936	20657	19183	21162	16196
Cieza	-1,4963438	38,283888	20338	20530	19940	24603	18263	19631	21137	22590	20353	20166	18588	20558	15774
Calasparra	-1,6947638	38,253487	19872	20178	19459	24186	17985	19171	20580	22408	19969	19854	18248	20174	14071
Calasparra	-1,5850018	38,269499	20637	20802	20169	24805	18402	19762	21285	22751	20536	20347	18822	20756	15712
Caravaca	-1,980057	38,043911	13691	14063	13082	18501	11480	13537	14085	16197	13402	13348	12975	14033	7108
Cehegin	-1,6828994	38,110901	17560	17644	17265	21533	15561	16881	18148	20066	17615	17454	15868	17781	11849
Moratalla	-1,813186	38,196653	16179	16469	15434	21920	13742	15864	16597	18730	15909	15889	15387	16556	12201
Cehegin	-1,7798922	38,104477	15562	15893	14991	21119	13223	15231	16019	18178	15403	15285	14825	15975	12661
Moratalla	-2,0961142	38,1145	8448	8605	8007	11637	6666	8234	8539	10916	8259	8294	7975	8689	6230
Vall de Uxo	-0,2304536	39,795861	22436	21944	21702	25966	19693	20381	22447	24783	21563	21336	19895	22013	16062
Onda	-0,2444114	39,954016	20445	19930	20256	24241	18126	18908	20923	23554	19909	20146	18742	20471	15457
San Rafael del Rio	0,3675272	40,594077	20623	19976	20212	24046	18325	18714	21073	23803	20252	20462	18616	20555	12433
Benicarlo	0,4014538	40,411511	22863	22273	22601	26193	20331	20833	23155	25950	22359	22452	20768	22707	15350
Castellon	-0,1191495	39,989342	21121	20622	21106	24723	18879	19291	21637	24185	20985	21067	19238	21168	14403
Burriana	-0,1057138	39,887849	23634	23139	22918	27137	20871	21525	23640	25928	22855	22615	21174	23221	14492
Ribera de Cabanes	0,1464314	40,133934	21139	20608	20986	24524	18661	19166	21556	24485	20776	20845	19228	21089	15515
Nules	-0,1683946	39,877237	23081	22361	22125	26374	19899	20829	23000	25043	21929	21642	20486	22433	15342
Segorbe	-0,4830876	39,817295	17679	16967	16730	21006	14933	15873	17517	19891	16693	16557	15472	17211	12538
Baza	-2,7677154	37,564477	17113	16436	16810	21214	15086	16551	18300	18442	17481	17407	15967	17346	8746
Puebla de Don Fadrique	-2,3817176	37,876115	10375	10640	9967	14608	8487	10460	10678	12907	10091	10160	9922	10754	6059
Loja	-4,138128	37,1693	18057	19084	18062	20843	16092	17596	18692	20927	18206	18052	16351	18360	11528
Iznalloz	-3,5514591	37,416406	16424	16989	17056	19168	15194	15977	17417	19604	17184	17056	15196	17024	7813

Jerez del Marquesado	-3,1498644	37,190536	12783	13058	13011	14809	11480	12411	13342	15419	13068	12999	11666	13095	6034
Cadiar	-3,183988	36,923123	16165	16543	16626	18501	14817	15715	16824	18848	16628	16455	14874	16545	8834
Zafarraya	-4,1538389	36,990314	12537	13056	12523	14417	10771	12263	12826	15151	12491	12420	11353	12710	6981
Padul	-3,600317	37,018743	16914	17417	17579	19455	15726	16456	17828	19905	17595	17452	15539	17442	10682
Granada	-3,6385645	37,172054	17678	18135	18411	20767	16459	17014	18650	20647	18476	18294	16383	18265	9930
Almuñecar	-3,6790578	36,751942	23040	23308	23208	25509	21240	22017	23685	25227	23289	22944	21481	23177	19144
Gibraleon	-7,0278022	37,318328	25345	26304	24983	28964	23873	24319	25319	27053	25652	25674	23811	25572	17580
Lepe	-7,2430825	37,302685	25185	25956	24887	28535	23448	23869	25453	27043	25415	25334	23836	25360	18243
Gibraleon	-7,059841	37,412354	23859	24665	23717	27298	22356	22832	23991	25978	24154	24125	22530	24137	16505
Moguer	-6,7925285	37,14648	24823	25641	24562	28523	23147	23779	25098	26848	25138	25148	23446	25105	16529
Niebla	-6,7353478	37,347125	24373	25381	23936	27926	22592	23261	24307	26266	24527	24583	22766	24538	15475
Aroche	-6,9449915	37,958077	18420	20119	18763	22508	17125	18461	18775	21785	18879	19351	17296	19226	13062
La puebla de Guzman	-7,2483655	37,552176	21916	22811	21899	25474	20549	21011	22186	24459	22375	22304	20686	22334	14447
El Campillo	-6,5992719	37,660989	21315	22283	21130	24768	19762	20500	21279	23789	21573	21616	20003	21638	14634
La Palma del Condado	-6,5415566	37,366968	22829	23839	22619	26514	21124	21830	22925	25020	23048	23062	21523	23121	16323
Almonte	-6,4765444	37,148345	25025	26076	24806	28575	23542	23849	25262	26861	25343	25301	23616	25296	17632
Valfarta	-0,1478858	41,531503	14131	13424	13725	15641	12441	12306	14812	16554	14032	14523	12165	13978	7854
Zaidin	0,2890014	41,637169	14644	13954	14164	16040	12992	12709	15291	16802	14488	14967	12590	14422	8991
Alcolea de Cinca	0,0731411	41,74095	13937	13292	13671	15411	12624	12354	14770	16163	13887	14344	12114	13870	9264
Tanarite de Litera	0,3771357	41,780947	13460	12956	13221	15053	12201	11998	14506	16027	13748	14145	12060	13579	8032
Lanaja	-0,337846	41,786429	13163	12671	12991	14662	11891	11662	13804	15963	12996	13504	11307	13147	7542
Monzon	0,1273494	41,957687	12352	11739	11984	13963	11091	10938	13219	14667	12713	13121	10930	12429	7986
Barbastro	0,1126102	42,013471	12229	11539	12131	13903	11171	10932	12928	14849	12363	12905	10782	12339	7606
Sariñena	-0,1766614	41,771411	13646	12974	13337	15171	12482	12225	14630	16207	13896	14272	11980	13711	8429
Huesca	-0,3777068	42,105429	12064	11595	11644	13328	10829	10598	12733	14541	12176	12594	10610	12065	7354
Candasnos	0,094436	41,45994	15231	14570	14593	16751	13554	13310	15773	17493	15094	15455	13254	15007	8357
Grañen	-0,3560041	41,942469	11984	11420	12038	13886	11021	10708	12987	14827	12370	12832	10696	12252	7471
Huerto	-0,1365362	41,966019	11859	11275	11863	13713	10824	10637	12830	14765	12321	12827	10494	12128	7532
Gurrea de Gallego	-0,7311994	41,992829	11368	11049	11479	14472	11645	10961	12567	14905	12717	13022	11390	12325	7380
Alfantega	0,1477817	41,821958	12328	11692	12390	14241	11258	11104	13309	15015	12767	13182	11028	12574	8660
Fraga	0,3539314	41,495165	15922	15006	15455	17161	14331	14080	16497	17982	15686	16031	13847	15636	10630
Tardienta	-0,5075831	41,969367	13028	12365	12591	14182	11819	11415	13845	15637	13021	13469	11220	12963	7498
San Esteban de Litera	0,3042037	41,882938	12134	11696	11972	13480	10924	10795	13054	14605	12388	12810	10776	12239	7885

Belver de Cinca	0,2318291	41,742536	13564	12773	13381	14707	12290	11917	14566	15759	13611	14099	11848	13501	8268
Alberuela de Tubo	-0,2573084	41,883957	13363	12761	13161	14985	12077	11790	14083	15919	13482	14001	11696	13392	8239
Jumilla	-1,4232837	38,394834	17390	17186	17051	21197	15161	16686	17905	19656	17290	17203	15626	17486	12831
Yecla	-1,1859032	38,658948	14869	14748	14517	18475	12865	14244	15324	17340	14729	14689	13401	15018	9356
Yecla	-1,1125211	38,562731	16437	16269	15978	20081	14276	15601	16807	18712	16298	16196	14721	16489	10386
Jumilla	-1,2407841	38,392588	16647	16509	16163	20506	14463	15919	17235	19088	16481	16378	14861	16750	14005
Jumilla	-1,3242866	38,31972	19307	19355	18759	23520	17151	18410	19966	21688	19272	19118	17545	19463	14101
Aitona	0,4609093	41,486913	16194	15359	15911	17514	14732	14300	17061	18544	16242	16606	14040	16046	10472
Albesa	0,6705502	41,760356	14458	13147	13513	15625	12883	12093	14139	15896	13241	13907	11014	13629	7850
Alcarras	0,5506119	41,56508	14926	13918	14599	16713	13162	13095	15838	17250	15054	15457	13292	14846	9669
Alfarras	0,5780224	41,819488	13396	12336	12526	14542	12028	11257	13288	14984	12369	12922	10306	12723	7900
Algerri	0,6483717	41,801036	13279	12068	12392	14251	11696	11164	13156	14603	12164	12775	10188	12521	7835
Alguaire	0,5361346	41,742812	12437	11845	12083	13795	11082	10952	13235	14681	12670	13071	11036	12444	7300
Castellnou de Seana	0,9520619	41,65659	14697	13465	13683	15918	12820	12212	14398	16010	13344	13988	11254	13799	8101
Cervera	1,2967772	41,662217	10845	9869	9959	11776	9398	8980	10746	12333	9737	10217	8163	10184	5778
Gimenells i el Pla de la Font	0,3933398	41,658132	13710	13029	13268	15254	12172	12095	14627	16156	13865	14183	11980	13667	8217
Golmes	0,9248038	41,63641	15011	13638	13950	16408	13386	12543	14603	16467	13620	14189	11447	14115	7850
Raimat	0,4490319	41,683272	12926	12258	12810	14621	11819	11518	14040	15592	13179	13677	11398	13076	7949
Balaguer- Monasterio Avellanas	0,7613663	41,879114	12365	11234	11518	13579	10994	10225	12105	13895	11350	11864	9457	11690	6546
El Canos	1,2041447	41,689385	11740	10655	10900	12724	10259	9646	11592	13294	10624	11172	8875	11044	6424
El Poal	0,8777387	41,672786	15010	13637	13950	16407	13385	12543	14603	16466	13619	14188	11446	14114	7942
Sant Marti de Riucorb	1,0885432	41,572353	13142	11967	11974	14281	11650	10751	12788	14692	11840	12343	9927	12305	7173
Seros	0,4279758	41,463784	16129	15307	15584	17669	14443	14306	17261	18339	16226	16657	14127	16004	10702
Tarrega	1,1626814	41,666945	12859	11788	11819	13819	11179	10611	12834	14264	11559	12141	9700	12052	7631
Tornabous	1,0451011	41,68834	13950	12787	13047	15228	12468	11497	13777	15531	12645	13266	10514	13156	7694
Vallfogona de Balaguer	0,8293888	41,784868	14024	12732	13030	15098	12345	11501	13814	15254	12747	13376	10646	13142	8188
Vilanova de Segria	0,628392	41,714499	14921	13808	13971	16148	13276	12602	14761	16292	13777	14348	11578	14135	8126
Lorca	-1,6294551	37,601733	20736	20221	19921	24837	18199	19852	21392	21866	20226	19939	19475	20606	14349
Lorca	-1,6938893	37,50379	22012	21534	21831	25707	20076	20842	23050	23392	22274	21914	20451	22098	14445
Agoncillo	-2,2904337	42,468182	9987	9195	9572	13183	8862	9356	11112	12534	10427	10792	8705	10339	7321
Albelda de Iregua	-2,4718558	42,380733	8846	8181	8746	11902	8169	8359	9540	11579	9336	9686	7929	9297,7	6251
Asenjo	-2,1533164	42,340952	9325	8907	9188	12620	8567	8922	10525	12299	9922	10252	8145	9879,4	6115
Logroño	-2,5136369	42,43969	9228	8683	8970	12484	8441	8789	10437	11976	10003	10370	8284	9787,8	6630

Santa Engracia del Juvera	-2,2629377	42,368971	9638	8887	9320	12822	8798	9010	10588	12298	10140	10531	8408	10040	5469
Aldea Nueva de Ebro	-1,9048679	42,222598	11604	10695	11091	15130	10501	10780	13021	14211	12191	12511	10103	11985	7993
Alfaro	-1,7776916	42,152119	10687	9744	10817	16022	10540	10638	11601	13066	11738	12262	10533	11604	7539
Calahorra	-2,001826	42,334834	11328	10534	10814	14846	10376	10618	12681	13996	11868	12322	9808	11745	7768
Corvera (Cabreton)	-1,8924542	42,006904	9672	8771	9452	14166	9194	9301	10362	12121	10402	10933	9194	10324	7174
Igea	-1,9937535	42,05775	8262	7730	8119	12176	7759	7964	8770	10852	8824	9295	7881	8875,5	6635
Quel	-2,037178	42,252488	10316	9753	10131	13678	9578	9868	11532	13385	10919	11356	9181	10882	6982
Rincon de Soto	-1,8508464	42,251583	11604	10695	11090	15130	10501	10780	13020	14211	12191	12510	10103	11985	7998
Aguilas	-1,5921627	37,418665	26485	25906	25507	30076	23697	25206	26853	28128	25855	25238	24511	26133	20895
Lorca	-1,8177885	37,855634	14965	15118	14262	19940	12435	14590	15223	17283	14611	14453	14049	15175	10245
Lorca	-1,623984	37,4878	22493	21759	21582	26672	19769	21197	22724	23727	22020	21459	21166	22233	17680
Puerto Lumbreras	-1,7255508	37,590472	21968	21429	21676	25608	20043	20874	23021	23278	22130	21855	20407	22026	14926
Mula	-1,4294482	38,065871	20925	20997	20573	25241	18748	19998	21595	23420	20928	20680	19220	21120	15868
Mula	-1,46674	38,041031	20485	20524	19900	24385	18147	19418	21001	22733	20227	20024	18390	20476	17461
Torres de Cotillas	-1,3025362	38,006971	23640	24082	23191	28176	21551	22646	24440	26053	23620	23406	21891	23881	18136
Molina del Segura	-1,2206884	38,127483	23198	23789	22526	27559	21111	22228	23884	25747	22947	22697	21353	23367	17740
Molina del Segura	-1,2336707	38,071139	23775	24082	23181	28346	21696	22811	24371	25979	23666	23367	21976	23932	16562
Abanilla	-1,0655079	38,170041	23783	23986	23056	28132	21458	22533	24237	25841	23576	23259	21816	23789	16357
Fortuna	-1,1526819	38,161028	23592	23763	23081	27884	21341	22466	24299	25663	23573	23293	21618	23688	18035
Ojos	-1,3394287	38,113316	21798	21978	21186	25973	19619	20811	22391	24279	21591	21385	19898	21901	20152
Beniel	-0,9997837	38,034507	24490	24637	23787	28702	22045	23282	24941	26450	24243	23919	22382	24443	17121
Murcia	-1,2682702	37,898166	21168	21071	20654	25221	18858	20079	21672	23425	21027	20720	19282	21198	20591
Murcia	-0,9840042	37,977528	23715	23791	23080	28117	21414	22475	24243	25816	23562	23303	21831	23759	19020
Murcia	-1,1347189	37,940075	22650	22688	22148	26911	20336	21515	23196	24865	22536	22177	20760	22707	18376
Fitero	-1,8426437	42,046077	9549	8619	9600	14434	9116	9329	10395	11864	10366	10892	9231	10309	7523
Cascante	-1,7239555	42,034371	11136	9970	10936	16277	10614	10801	11835	13396	11924	12364	10738	11817	8404
Ablitas	-1,6447131	41,996446	12843	12455	12932	15991	12957	12034	14144	16275	13953	14352	12557	13681	8014
Murillo el fruto	-1,4871859	42,38498	11173	10538	10593	14097	9828	10469	12396	13644	11276	11559	9943	11411	7240
Miranda de Arga	-1,8087315	42,511252	10362	9717	10199	13711	9558	9766	11623	13144	10861	11268	9236	10859	6931
Falces	-1,7925482	42,409669	10461	9709	10296	14186	9677	10006	11756	13266	11121	11511	9214	11019	7716
Bargota	-2,2992201	42,477657	9986	9195	9572	13183	8862	9356	11112	12533	10427	10792	8705	10339	7798
Bardenas Reales	-1,5187546	42,295154	10908	10046	10286	13646	9768	10365	12482	13108	11032	11391	9748	11162	7954
Los Arcos	-2,1845206	42,539308	9129	8658	8965	12387	8288	8654	10273	12030	9661	10122	8062	9657,1	7131

Sesma	-2,126631	42,473409	9665	8871	9462	13108	8885	9185	10910	12405	10149	10609	8455	10155	7237
Cadreita	-1,6556731	42,26433	11919	10957	10811	14819	10530	11126	13415	13889	12041	12337	10365	12019	7244
Bardenas Reales	-1,7183027	42,207768	11607	10922	11270	15124	10487	10895	13074	14242	12181	12499	10210	12046	7246
Sartaguda	-2,0512344	42,361948	10933	10197	10681	14455	10108	10492	12102	13594	11493	11912	9683	11423	6674
Olite	-1,662579	42,423779	11000	10245	10265	13817	9853	10456	12475	13225	11035	11338	9778	11226	6528
Murillo el Cuende	-1,6153521	42,361474	11115	10235	10215	13902	9786	10389	12343	13236	11120	11513	9908	11251	7035
Corella	-1,8398436	42,115577	10565	9450	10654	15752	10193	10378	11444	12605	11355	11793	10189	11307	6379
Funes	-1,8066789	42,287885	11573	10946	11312	15039	10672	10959	12973	14357	12180	12572	10305	12081	6172
Lerin	-1,9763006	42,503595	10331	9641	9961	13423	9366	9693	11538	12869	10718	11049	9048	10694	6293
Los Palacios y Villafranca	-5,9390554	37,179127	24576	25460	23955	27986	22613	23607	25101	26491	24586	24552	22624	24686	16993
Las cabezas de San Juan	-5,884722	37,01556	24885	25760	24409	28235	23012	24043	25575	26792	25030	24912	23163	25074	17551
Lebrija	-6,1261602	36,976641	25308	26284	24724	28612	23356	24322	25754	27002	25296	25216	23444	25393	16160
Aznalcazar	-6,2733503	37,151795	24375	25165	23873	27936	22489	22921	24292	26016	24462	24439	22647	24420	16078
Isla Mayor	-6,1512787	37,098521	24614	25508	24343	28059	22823	23616	25380	26404	24933	24785	22930	24854	17349
La puebla del Rio	-6,1338321	37,226032	24614	25729	24244	27916	22807	23632	25135	26549	24742	24593	22674	24785	16764
La puebla del Rio II	-6,0465691	37,080174	24684	25617	24400	28179	22872	23454	25437	26552	25001	24754	23054	24910	16884
Ecija	-5,0770704	37,592934	21881	23048	22324	24887	20505	21273	23256	24577	22643	22298	20575	22479	14839
La Luisiana	-5,2281407	37,525293	22032	23024	21538	25544	20311	21243	22768	24369	22175	22177	20320	22318	14321
Carmona	-5,587615	37,400903	23703	24651	23248	27257	21898	22713	24299	25848	23969	23849	22031	23952	15593
Osuna	-5,1348377	37,25503	22513	23419	22106	25856	20668	21544	23119	24610	22620	22510	20730	22700	15003
La Rinconada	-5,924839	37,456832	24406	25567	23965	27477	22619	23524	25123	26316	24546	24411	22369	24575	16711
Sanlucar La Mayor	-6,2550749	37,42179	23302	24437	22960	27134	21660	22329	23225	25347	23518	23611	21811	23576	16128
Villanueva del Rio y Minas	-5,6840093	37,613036	23743	24731	23327	27301	21921	22842	24401	25705	23914	23820	21829	23958	16285
Lora del Rio	-5,5407037	37,660906	22508	23632	22117	26272	20815	21641	23138	24682	22782	22783	20541	22810	15889
Los Molares	-5,6729697	37,176152	23963	24919	23635	27471	22292	23173	24734	26031	24208	24116	22399	24267	15547
Guillena	-6,06419	37,514568	24023	24903	23714	27405	22204	23253	24659	26063	24249	24191	22334	24273	17550
Puebla Cazalla	-5,3509152	37,218131	22316	23253	21886	26055	20448	21684	22943	24659	22385	22396	20650	22607	16263
Alcala del Rio	-5,9641033	37,512529	24522	25593	24152	27901	22941	23601	25351	26406	24774	24695	23023	24814	16502
San Javier	-0,819705	37,791664	26673	26809	25721	30718	24010	25154	26948	28870	26306	25748	24704	26515	18431
Torre Pacheco	-0,8985888	37,773803	26862	26847	26137	30962	24386	25339	27272	28832	26658	26127	24820	26749	16515
San Javier	-0,8836862	37,848045	26136	26374	25282	30304	23627	24864	26572	28465	25772	25398	24188	26089	17803
Torre Pacheco	-0,9316281	37,823827	25703	25885	24940	30027	23339	24416	26117	27819	25409	25022	23719	25672	17435
Torre Pacheco	-0,9867861	37,74765	26403	26769	25609	30813	24117	25028	26868	28705	26190	25817	24392	26428	17124

Pedralba	-0,7175861	39,567014	19820	20040	19801	23903	18106	18775	20391	22750	19990	19981	18127	20153	15377
Liria	-0,627062	39,691055	19797	20128	20170	23625	18320	18860	20232	22632	20056	19967	18183	20179	13811
Benifayo	-0,4618662	39,280627	24323	23761	23449	27689	21323	22046	24277	26023	23437	23133	21650	23738	17141
Cheste	-0,7444395	39,518889	18391	18625	18320	22544	16510	17357	18807	21299	18401	18410	16656	18665	14095
Tabernes de Valldigna	-0,2380292	39,095261	23504	22913	22599	27297	20536	21432	23661	25655	22790	22395	20969	23068	18680
Villanueva de Castellon	-0,5242892	39,065567	23367	22865	22635	27050	20473	21236	23337	25249	22544	22261	20889	22900	16526
Sagunto	-0,2663216	39,647534	23327	22800	22582	26730	20334	21183	23253	25431	22408	22075	20734	22805	17992
Benavites	-0,2162186	39,730391	22456	21847	21704	26144	19693	20374	22453	24839	21558	21386	20139	22054	15340
Moncada	-0,3989602	39,587729	22911	22314	22068	26571	19978	20833	22989	25131	22047	21715	20515	22461	16163
Carcagente	-0,4461657	39,113604	23729	23162	22989	27335	20806	21756	23759	25490	23005	22638	21230	23264	15405
Carlet	-0,5459462	39,22642	21539	21906	21561	26151	19788	20493	22179	24451	21868	21795	19720	21950	16337
Luchente	-0,3600825	38,938508	19264	18389	18202	22830	16070	17104	19119	21057	18292	18052	16593	18634	12858
Requena	-1,2323883	39,504667	11144	11466	11468	14602	9920	11001	11420	14162	11421	11609	10145	11669	6847
Algemesi	-0,4353656	39,216442	24255	23683	23326	27823	21291	22138	24216	26002	23321	22968	21755	23707	16576
Campo Arcis	-1,1622154	39,433357	11829	12154	11965	15378	10475	11266	12085	14878	12121	12264	10680	12281	8411
Betera	-0,4685258	39,597708	22137	21577	21360	25693	19344	20129	22124	24258	21342	21031	19552	21686	15132
Picasent	-0,4976324	39,362484	22871	22189	21927	26525	19790	20716	22780	24845	21838	21554	20211	22295	15768
Montesa	-0,6383798	38,954502	18846	18971	18692	23211	16982	18023	19092	21696	18917	18919	17102	19132	15324
Jativa	-0,5497109	38,998803	22503	21911	21529	26162	19286	20482	22504	24286	21405	21141	19753	21906	15352
Villalonga	-0,2042579	38,892111	22902	22360	21931	26399	19643	20748	22917	24850	21845	21508	20199	22300	17148
Gandia	-0,2506841	38,964297	21441	20986	20678	25229	18471	19471	21531	23765	20627	20426	18923	21050	16252
Bolbaite	-0,6901658	39,069153	18555	18741	18696	22913	16773	17729	18856	21630	18723	18778	17009	18946	14060
Chulilla	-0,8322414	39,67678	17824	18307	18024	21901	16288	16977	18293	20773	18080	18127	16318	18265	12949
Almoacid de la Sierra	-1,3299642	41,452078	13129	12878	13182	16102	13114	12545	13985	16611	14259	14571	12940	13938	9393
Belchite	-0,7216155	41,350306	14134	13506	13950	17131	13737	13303	15176	17413	15014	15210	13521	14736	8981
Quinto	-0,5186373	41,388348	16036	15611	15688	17135	14716	14304	17118	18497	16083	16443	13865	15954	9761
Fabara	0,1540344	41,167877	16172	15489	15597	17686	14410	14261	17174	18515	16026	16304	13976	15965	9435
Epila	-1,2820466	41,583234	13946	13671	14090	17535	14094	13303	15173	17569	15358	15700	13485	14902	9369
Ejea de los Caballeros	-1,1961298	42,097715	11367	11242	11477	14341	11540	10728	12548	15004	12443	12787	11165	12240	7350
Sabada	-1,309387	42,267312	11290	10286	10385	14058	9922	10591	12740	13559	11271	11586	10034	11429	6618
Luna	-0,9359498	42,095761	10630	10397	10911	13783	10696	10059	11768	14028	11655	12043	10344	11483	6204
Santa Engracia	-1,3305062	41,921246	13464	13210	13625	16404	13142	12861	14574	16797	14470	14729	13148	14220	8541
Pastriz	-0,7461599	41,59514	13952	13378	14090	17116	14048	13158	15273	17451	15247	15561	13483	14796	9507

Zaragoza	-0,823819	41,71363	12663	12456	13035	15880	13088	12134	14207	16300	14123	14405	12533	13711	9114
Calatayud	-1,6583593	41,332167	9543	9125	9659	12727	9475	9065	10506	12815	10704	11089	9493	10382	7377
Borja	-1,5076903	41,855146	13001	12459	12788	15862	12524	12019	13878	16203	13796	14115	12498	13558	8278
Tarazona	-1,7458841	41,916177	9664	8753	9588	14200	9043	9278	10005	12172	10339	10778	9238	10278	7465
Caspe	-0,0710973	41,303968	17002	16301	16211	18219	15035	14868	17701	19175	16921	17183	14837	16678	9995
Osera de Ebro	-0,5363755	41,545081	15059	14797	14700	16312	13662	13263	16143	17754	15278	15550	13312	15075	9589
Daroca	-1,4247095	41,108073	7602	7448	7696	10228	7401	7409	8101	10626	8623	9006	7542	8334,7	6089
Zuera	-0,7511391	41,869389	12032	11756	12131	15187	12206	11498	13349	15560	13271	13545	11822	12941	9049
El Bayo	-1,2487734	42,175713	11050	10940	11271	14460	11100	10382	12095	14680	12300	12692	10825	11981	7044
Tauste	-1,1428386	42,00023	13007	12905	13087	16182	13169	12296	14351	16568	14146	14506	12480	13881	8384
Boquiñeni	-1,2496818	41,843217	13165	13066	13426	16457	13536	12775	14653	16997	14764	15117	13257	14292	9069
