# Supplementary figures and images for: Agroclimatic Metrics for the Main Stone Fruit Producing Areas in Spain in Current and Future Climate Change Scenarios: Implications From an Adaptive Point of View
Source: Front Plant Sci. 2022 Jun 8;13:842628. doi: 10.3389/fpls.2022.842628 (PMC9213681; doi:10.3389/fpls.2022.842628)

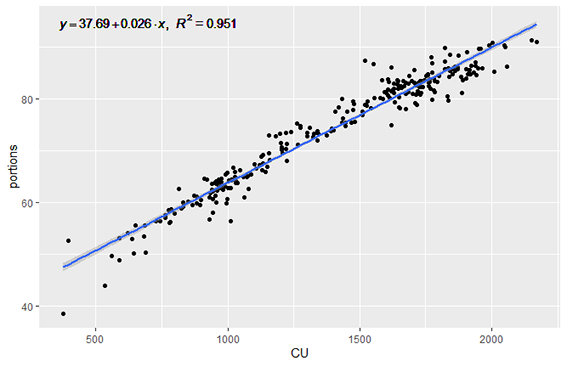

Supplement: Supplementary Figure 1 — Correlation between mean accumulated portions and chill units for the current scenario in all the weather stations. [file Image_1.tiff]

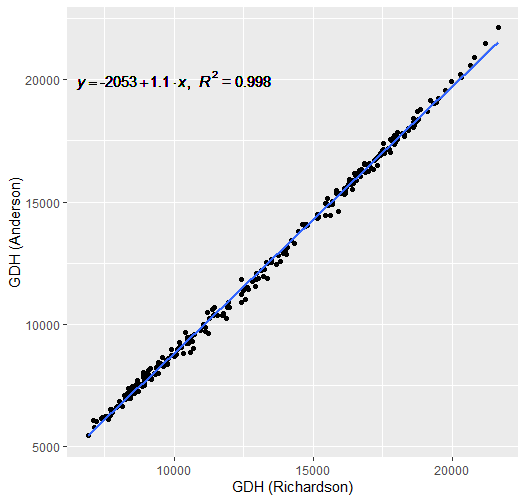

Supplement: Supplementary Figure 2 — Correlation between mean accumulated GDH for Anderson and Richardson models for the current scenario in all the weather stations. [file Image_2.tiff]
